# Short- and long-term comparative effectiveness of nirmatrelvir/ritonavir and molnupiravir in asthma patients: a cohort study

**DOI:** 10.1186/s12931-025-03156-2

**Published:** 2025-02-28

**Authors:** Guozhang Lin, Yuchen Wei, Zihao Guo, Huwen Wang, Kate Ching Ching Chan, Renee Wan Yi Chan, Chi Tim Hung, Xiaoting Jiang, Conglu Li, Carrie Ho Kwan Yam, Tsz Yu Chow, Yawen Wang, Shi Zhao, Kehang Li, Aimin Yang, Chris Ka Pun Mok, David S. C. Hui, Eng Kiong Yeoh, Ka Chun Chong

**Affiliations:** 1 School of Public Health and Primary Care, The Chinese University of Hong Kong, Hong Kong, Hong Kong Special Administrative Region China; 2 Department of Paediatrics, Prince of Wales Hospital, The Chinese University of Hong Kong, Shatin, Hong Kong Special Administrative Region China; 3 Division of Landscape Architecture, Department of Architecture, Faculty of Architecture, The University of Hong Kong, Pokfulam, Hong Kong Special Administrative Region China; 4 School of Public Health, Tianjin Medical University, Tianjin, China; 5 Department of Medicine & Therapeutics, Faculty of Medicine, The Chinese University of Hong Kong, Hong Kong, Hong Kong Special Administrative Region China; 6 Li Ka Shing Institute of Health Sciences, Chinese University of Hong Kong, Hong Kong, Hong Kong Special Administrative Region China; 7 S.H. Ho Research Centre for Infectious Diseases, Chinese University of Hong Kong, Hong Kong, Hong Kong Special Administrative Region China; 8 Centre for Health Systems and Policy Research, The Chinese University of Hong Kong, Hong Kong Special Administrative Region, China

**Keywords:** Asthma, COVID-19, Effectiveness, Molnupiravir, Nirmatrelvir/ritonavir

## Abstract

**Background:**

Few studies evaluated the effectiveness of COVID-19 antivirals specifically in the asthma population This study assessed short- and long-term effects of nirmatrelvir/ritonavir versus molnupiravir in asthma population.

**Methods:**

This is a retrospective cohort study on adult asthma patients infected with COVID-19, using real-world data obtained from the health officials in Hong Kong. Key inclusion criteria were infection with COVID-19 between March 16, 2022, and Oct 30, 2023, age ≥ 18 years, previous asthma diagnosis, and prescription history of an asthma medication. Outcomes included acute and post-acute mortality, post-acute all-cause hospitalization, and cause-specific hospitalization.

**Results:**

1,745 patients were eligible for this study, with a median follow-up time of 365 days (IQR: 335–365). Patients in the nirmatrelvir/ritonavir group had significantly lower risks of acute inpatient death (HR, 0·27 [95% CI, 0·12 to 0·59]; *p* = 0·0011), post-acute inpatient death (HR, 0·49 [95% CI, 0·28 to 0·85]; *p* = 0·011), all-cause hospitalization (HR, 0·72 [95% CI, 0·58 to 0·89]; *p* = 0·0020), and myocardial infarction (HR, 0·10 [95% CI, 0·01 to 0·92]; *p* = 0·042) than patients in the control group. The risk of all-cause hospitalization was significantly lower in the nirmatrelvir/ritonavir group compared to the molnupiravir group (HR, 0·65 [95% CI, 0·52 to 0·81]; *p* = 0·00012). Among patients who were prescribed medium-/ high-dose inhaled corticosteroids, the nirmatrelvir/ritonavir group had a lower hazard of asthma exacerbation than the molnupiravir group (HR, 0·58 [95% CI, 0·35 to 0·95]; *p* = 0.030).

**Conclusion:**

Compared with molnupiravir, nirmatrelvir/ritonavir may offer more benefits in reducing the risk of post-acute sequelae of COVID-19 among asthma patients. In addition, the post-acute benefits of the antivirals were also demonstrated in patients with mild asthma, which have not been generally recommended in existing clinical management guidelines.

**Supplementary Information:**

The online version contains supplementary material available at 10.1186/s12931-025-03156-2.

## Introduction

The global outbreak of COVID-19 has led to millions of related deaths, raising a significant subsequent public health concern about the persistent or newly onset symptoms following the acute phase of SARS-CoV-2 infection, known as post-COVID-19 condition (PCC), as well as post-acute sequelae of COVID-19. According to the scoping review by Hayes et al. [1], more than 100 persistent symptoms of SARS-CoV-2 infection were identified, affecting multiple systems such as the respiratory, neurological, cardiovascular, and musculoskeletal systems. Another study, employing study populations from the United Kingdom (UK) and Hong Kong, identified 13 major post-acute sequelae of COVID-19 infection [2]. A systematic review [3] reported that at least 45% of COVID-19 cases experienced at least one PCC.

In December 2021, two COVID-19 antivirals, namely nirmatrelvir combined with the boosting agent ritonavir (nirmatrelvir/ritonavir) and molnupiravir, were granted emergency use authorization by the United States Food and Drug Administration (FDA) for treating non-hospitalized patients at high risk of progressing to severe COVID-19 [4, 5]. According to the EPIC-HR trial, nirmatrelvir/ritonavir was effective in reducing the risk of severe COVID-19 outcomes by day 28 after an acute infection [6]. A similar effectiveness finding was also reported for the molnupiravir treatment [7]. In addition, the prolonged benefit of early treatments of nirmatrelvir/ritonavir and molnupiravir on PCCs in hospitalized and non-hospitalized patients were reported by several studies [8–10]. The choice between nirmatrelvir/ritonavir and molnupiravir for COVID-19 treatment mainly depends on patient risk factors and timing [11]. Nirmatrelvir/ritonavir is preferred for high-risk individuals within five days of symptom onset. Molnupiravir can be used when nirmatrelvir/ritonavir is contraindicated (e.g., drug interactions, severe renal or liver impairment). Low-risk patients or those beyond the five-day window may be prescribed neither of the antivirals due to the potentially reduced benefits.

According to the existing evidence, asthma is unlikely to be associated with an increased risk of severe COVID-19 in general [12, 13]. Nevertheless, severe asthma was found to be a risk factor of COVID-19 related death during the early phase of pandemic [14]. In addition, a large UK cohort study indicated that patients using three or more asthma medications had an increased risk of hospitalization and death due to COVID-19 [15]. Supported by the findings, moderate-to-severe asthma is considered a risk factor for antiviral prescription for SARS-CoV-2 infection [16]. In relation to PCCs, conflicting results have been reported. Garcia-Pachon et al. demonstrated that patients with asthma had a lower prevalence of post-COVID syndrome than the general COVID-19 patient population [17], whereas Wang et al. indicated that patients with asthma faced a higher risk of post-acute respiratory symptoms such as shortness of breath and cough [18].

Patients with asthma are typically understudied in randomized controlled trials (RCTs) for COVID-19, especially in studies focusing on the PCC outcomes necessitating a longer follow-up period. While studies have assessed the effectiveness of the antivirals among patients with chronic respiratory diseases [19–21], none have been specifically designed for patients with asthma and studied with sufficiently long follow-up for post-COVID-19 outcomes. To bridge this research gap, this study investigated the long-term benefits of the antivirals specifically on the asthma population, adjusting for covariates relevant to asthma (e.g., chronic obstructive pulmonary disease, inhaled corticosteroid dose level, and use of oral glucocorticoid) and conducting analysis specific to asthma (e.g., outcome of asthma exacerbation and subgroup analysis by inhaled corticosteroid dose level). In this study, we examined the association of the COVID-19 antivirals with the acute and post-acute COVID-19 outcomes, including mortality, all-cause hospitalization, and hospitalizations due to several major sequelae among patients with asthma in Hong Kong. Our study is expected to profile the short- and long-term benefits of antivirals in this underrepresented population, with the goal of supporting the suggested therapeutic approach for patients with asthma who contract COVID-19 [22].

## Methods

### Study design

This is a retrospective cohort study that utilized territory-wide data obtained from Hong Kong Hospital Authority (HA) and Department of Health (DH). The HA is a statutory body that manages all public hospitals in Hong Kong. It provides public inpatient and outpatient services to over 7.3 million residents, covering approximately 80% of hospital admissions as well as all COVID-19 patients in the territory [2]. The centralized database maintained by the HA encompasses individual-level information on patient demographics, diagnoses, and prescribed medications. The comprehensive electronic health records from the HA were linked to the COVID-19 vaccination records from the DH using anonymized pseudo numbers. These datasets have been employed in numerous studies for analyzing COVID-19-related outcomes and the effectiveness of treatments [10].

### Patients

This study identified patients infected with SARS-CoV-2 by a positive reverse transcription-polymerase chain reaction (RT-PCR) test result or an attendance at a COVID-19 designated clinic. A patient’s attendance at a designated clinic implied a positive result from either a rapid antigen test or an RT-PCR test, because such a result was required for the attendance. For a patient identified by a positive RT-PCR test result, the index date was defined as the date of the test result. For a patient identified by an attendance at a designated clinic, the index date was defined as the date of attendance.

The inclusion criteria were as follows:

1. an index date of SARS-CoV-2 infection between March 16, 2022 (the date when nirmatrelvir/ritonavir became available in Hong Kong) and Oct 30, 2023 (31 days before the end of data availability, which was Nov 30, 2023), during which Omicron variant was the predominant strain;
2. age ≥ 18 years;
3. a previous asthma diagnosis (ICD-9-CM code of “493.xx” in the inpatient records or ICPC-2 code of “R96” in the outpatient records) AND prescription history of any asthma medication within 12 months before or on the index date (Supplement Table 1) [23].

**Table 1:** Baseline characteristics of eligible patients before weighting

Characteristic	Nirmatrelvir/ritonavir group	Molnupiravir Group	Control Group
N	621	378	746
Age (median [IQR])	70.00 [55.00, 80.00]	77.00 [67.00, 86.00]	72.00 [50.00, 85.00]
Sex			
Female (%)	384 (61.8)	227 (60.1)	431 (57.8)
Male (%)	237 (38.2)	151 (39.9)	315 (42.2)
Vaccination status (%)			
Unvaccinated	73 (11.8)	71 (18.8)	165 (22.1)
1–2 doses	118 (19.0)	82 (21.7)	222 (29.8)
≥3 doses	430 (69.2)	225 (59.5)	359 (48.1)
Hospital admission (%)	241 (38.8)	156 (41.3)	336 (45.0)
Concomitant pharmacological treatments			
Dexamethasone (%)	28 (4.5)	11 (2.9)	147 (19.7)
Prednisolone (%)	36 (5.8)	28 (7.4)	49 (6.6)
Remdesivir (%)	12 (1.9)	5 (1.3)	134 (18.0)
Comorbidity			
Arrhythmia (%)	29 (4.7)	75 (19.8)	71 (9.5)
Cerebrovascular disease (%)	25 (4.0)	46 (12.2)	57 (7.6)
Chronic obstructive pulmonary disease (%)	55 (8.9)	40 (10.6)	106 (14.2)
Congestive heart failure (%)	31 (5.0)	58 (15.3)	104 (13.9)
Coronary artery disease (%)	36 (5.8)	46 (12.2)	86 (11.5)
Diabetes (%)	47 (7.6)	57 (15.1)	94 (12.6)
Hypertension (%)	109 (17.6)	114 (30.2)	179 (24.0)
Liver disease (%)	15 (2.4)	23 (6.1)	31 (4.2)
Malignancy (%)	26 (4.2)	33 (8.7)	40 (5.4)
Renal disease (%)	5 (0.8)	22 (5.8)	12 (1.6)
Recent use of an oral glucocorticoid (%)	108 (17.4)	106 (28.0)	223 (29.9)
ICS dose (%)			
None	239 (38.5)	109 (28.8)	258 (34.6)
Low	133 (21.4)	71 (18.8)	147 (19.7)
Medium	186 (30.0)	127 (33.6)	252 (33.8)
High	63 (10.1)	71 (18.8)	89 (11.9)

The exclusion criteria were as follows [10, 24]:

1. a contraindication to nirmatrelvir/ritonavir due to drug interaction (that is, prescription history of amiodarone, apalutamide, carbamazepine, ivosidenib, lumacaftor-ivacaftor, phenobarbital, phenytoin, primidone, rifampicin, rifapentine, or St John’s Wort within 90 days before the index date);
2. severe renal impairment (that is, estimated glomerular filtration rate < 30 mL/min per 1·73 m², dialysis, or renal transplantation);
3. severe liver impairment (that is, cirrhosis, hepatocellular carcinoma, or liver transplantation);
4. prescription history of both nirmatrelvir/ritonavir and molnupiravir within 30 days of the index date;
5. prescription history of nirmatrelvir/ritonavir or molnupiravir, but not within 5 days of the index date.

The eligible patients were categorized into three groups. The nirmatrelvir/ritonavir group consisted of patients who were prescribed nirmatrelvir/ritonavir within five days of the index date, while the molnupiravir group comprised patients who were prescribed molnupiravir within five days of the index date. The control group consisted of patients who were not prescribed nirmatrelvir/ritonavir or molnupiravir within 30 days of the index date. Three comparisons were made among the three groups, which were the nirmatrelvir/ritonavir group vs. molnupiravir group, molnupiravir group vs. control group, and nirmatrelvir/ritonavir group vs. molnupiravir group. The first two comparisons were to assess the effectiveness of each antiviral separately, while the third comparison was to compare benefits of the two antivirals.

### Outcomes

To examine the effectiveness of the antivirals across different phases, we studied both acute outcomes (from day 0 to day 30 following the index date) and post-acute outcomes (from day 31 to day 365). The acute outcomes were: (1) inpatient death (More than 90% of deaths in Hong Kong occurred in hospitals, especially public hospitals [25]); (2) ICU admission or respiratory support (ICD-9-CM codes in Supplement Table 2). The post-acute outcomes were: (1) inpatient death; (2) ICU admission or respiratory support; (3) all-cause hospitalization; (4)–(7) cause-specific hospitalization due to incident conditions including chronic pulmonary disease excluding asthma, acute respiratory distress syndrome, myocardial infarction, and stroke (pulmonary and cardiovascular conditions commonly studied in post-COVID-19 literature [2]); (8) asthma exacerbation, which was defined as hospital admission with asthma as the principal diagnosis OR prescription history of an oral glucocorticoid during an outpatient attendance for asthma or during an accident and emergency (A&E) attendance with a dose < 300 mg (to ensure a normal dose for treating asthma exacerbation) [26]. In summary, inpatient death and ICU admission or respiratory support were ascertained from Day 0 to Day 30 and from Day 31 to Day 365 following the index date to investigate the short- and long-term effectiveness of the antivirals in reducing severe outcomes. The other outcomes were ascertained from Day 31 to Day 365 in line with existing literature on post-acute sequelae of COVID-19 [2, 27].

In the analyses of the cause-specific hospitalization outcomes, individuals with a history of the corresponding condition before the index date were excluded. The ICD-9-CM codes for the conditions can be found in Supplement Table 2.

Every patient was followed up until inpatient death, the occurrence of the outcome in the corresponding analysis, 365 days after the index date, or the end of data availability (Nov 30, 2023), whichever came first.

### Covariates

The covariates adjusted for in this study covered demographics, information relevant to the SARS-CoV-2 infection, comorbidities, and prescription history [24, 28, 29]. They were:

1. age;
2. sex;
3. COVID-19 vaccination status (unvaccinated, 1–2 doses, or ≥ 3 doses);
4. week of the index date;
5. hospital admission on the index date;
6. initiation of concomitant pharmacological treatments on the index date, including dexamethasone, prednisolone, and remdesivir;
7. comorbidities, including arrhythmia, cerebrovascular disease, chronic obstructive pulmonary disease, congestive heart failure, coronary artery disease, diabetes, hypertension. liver disease, malignancy, and renal disease (ICD-9-CM codes provided in Supplement Table 3);
8. oral glucocorticoid prescription history with a dose < 300 mg within one year before the index date;
9. the highest inhaled corticosteroid (ICS) dose level prescribed within one year before or on the index date, where the levels were defined according to Global Strategy for Asthma Management and Prevention (2024 update) [29].

### Statistical analysis

Before adjusting for the covariates, we plotted cumulative incidence curves for outcomes of inpatient death, ICU admission or respiratory support, all-cause hospitalization, and asthma exacerbation stratified by the use of antivirals. Cumulative incidence curves were calculated by summing, at each event time, the product of the probability of being event-free just before the event time (estimated by the Kaplan-Meier estimator) and the hazard of the event at that time (estimated by the number of patients having the event at that time divided by the number of patients at risk at that time) [30].

We employed standardized mortality ratio (SMR) weighting to adjust for the covariates [31], with the propensity scores derived from logistic regression models. The balance of each covariate before and after weighting was assessed according to the absolute value of the standardized mean difference (SMD). We included the imbalanced covariates after weighting (SMD ≥ 0·1) in the corresponding models for doubly robust adjustment [32].

To evaluate the effectiveness of the antivirals, Cox proportional hazards models were applied in the weighted samples. The assumption of proportional hazards was assessed based on plots of scaled Schoenfeld residuals.

For the outcomes of inpatient death, ICU admission or respiratory support, all-cause hospitalization, and asthma exacerbation, we did subgroup analyses by vaccination status (< 3 doses, or ≥ 3 doses), by ICS dose level (none or low, or medium or high), and by whether the patient was admitted to hospital on the index date. Three sensitivity analyses were conducted. The first sensitivity analysis was comparison between the molnupiravir group and control group with the start of the study period as the date when molnupiravir became available (Feb. 26, 2022) and without excluding individuals with contraindications to nirmatrelvir/ritonavir. In the second and third sensitivity analyses, we excluded patients who were prescribed salmeterol within 10 days of the index date from the comparisons involving nirmatrelvir/ritonavir (i.e., nirmatrelvir/ritonavir group vs. control group and nirmatrelvir/ritonavir group vs. molnupiravir group), due to potential drug interaction.

All analyses were performed using R (version 4.3.2) (R Program for Statistical Computing).

## Results

**Fig. 1:**
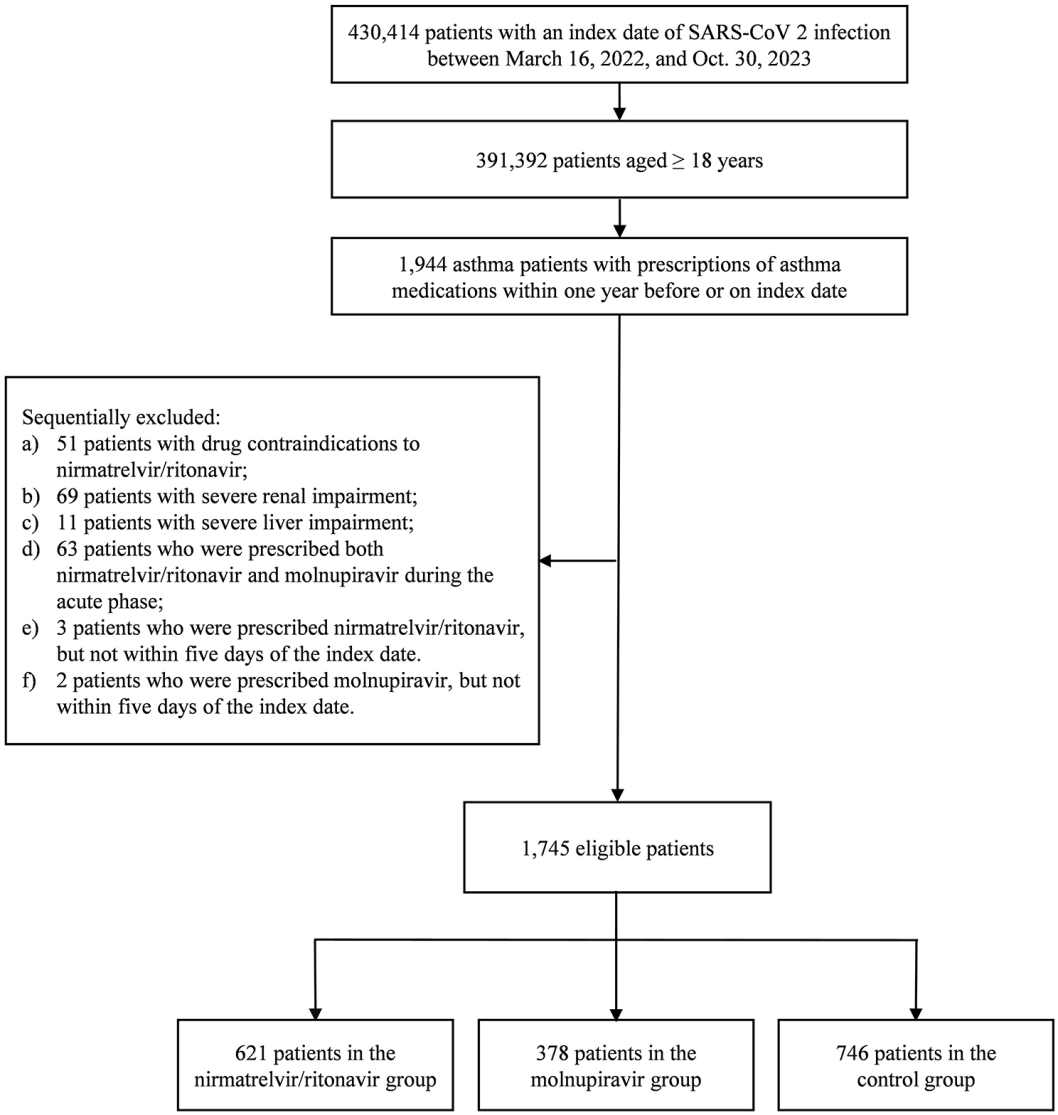
Flowchart of patient inclusion and exclusion

As Fig. 1 shows, of 430,414 patients with an index date of SARS-CoV-2 infection between March 16, 2022 (the date when nirmatrelvir/ritonavir became available in Hong Kong), and Oct. 30, 2023, 1,944 were adult asthma patients (aged ≥ 18 years) with prescriptions of asthma medications within one year before or on the index date. Among the 1,944 patients, 1,745 were eligible for the analysis, with 621 patients in the nirmatrelvir/ritonavir group, 378 patients in the molnupiravir group, and 746 patients in the control group, and a median follow-up time of 365 days (IQR: 335–365).

Before standardized mortality ratio (SMR) weighting, among the three groups, the molnupiravir group had the largest median age (77·0 years), followed by the control group (72·0 years) and the nirmatrelvir/ritonavir group (70·0 years). All three groups had higher proportions of females (61·8% [384/621] in the nirmatrelvir/ritonavir group, 60.1% [227/378] in the molnupiravir group, and 57·8% [431/746] in the control group) (Table 1). The standardized mean differences of patient characteristics between each pair of the three groups are provided in Supplement Table 4 A–4 C. The severity of patients at baseline defined based on prescription of concomitant pharmacological treatments is described in Supplement Table 6.

**Fig. 2:**
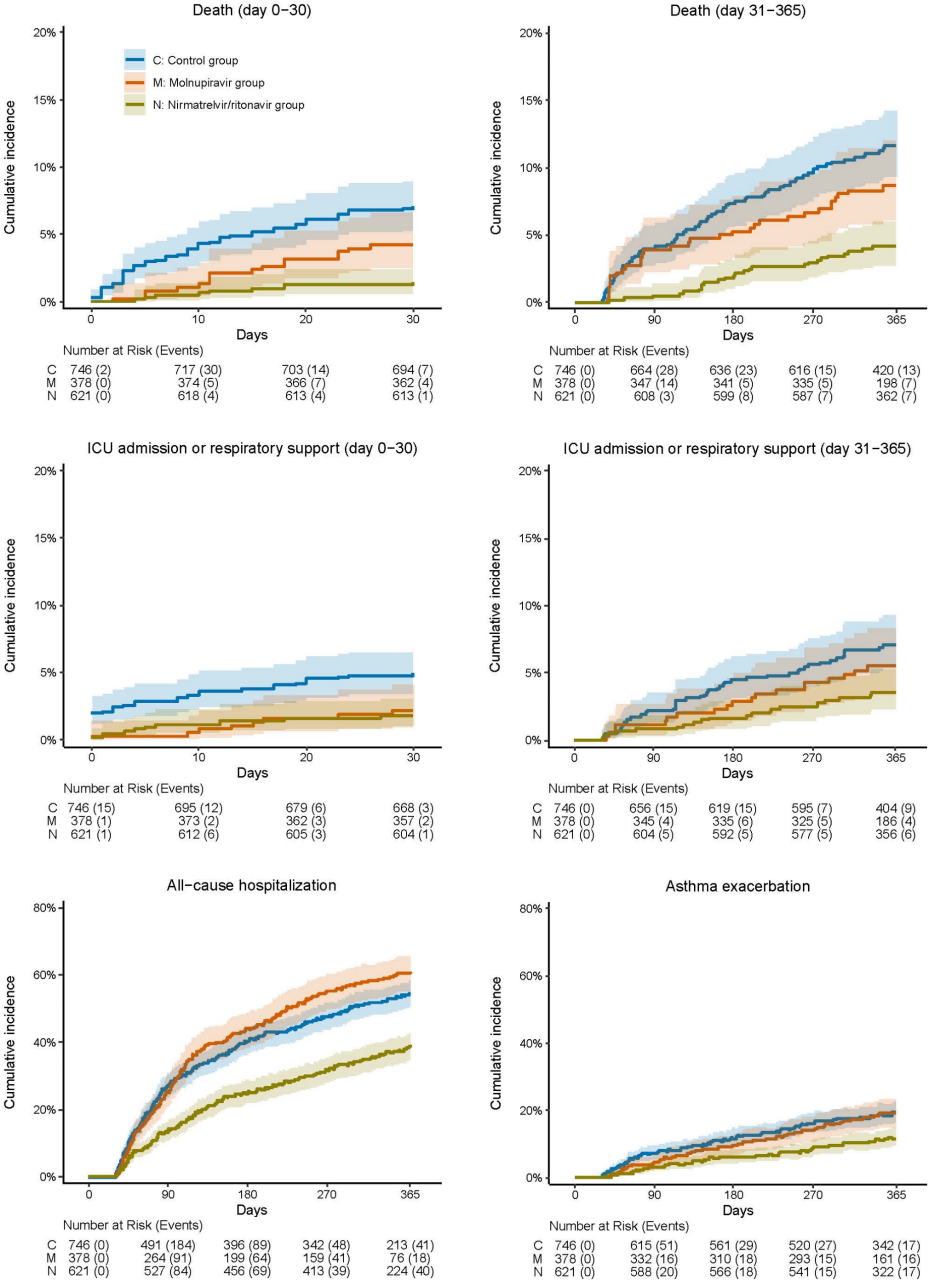
Unadjusted cumulative incidence curves with risk tables for selected outcomes (outcomes ascertained from day 31 to day 365, unless otherwise specified)

The unadjusted cumulative incidence curves with risk tables were plotted in Fig. 2. The patients in the control group had higher unadjusted cumulative incidences at most of the times than the patients in the antiviral groups for all selected outcomes except all-cause hospitalization, where the molnupiravir group had higher unadjusted cumulative incidences approximately from 100 days on.

Patient characteristics with standardized mean differences after SMR weighting are provided in Supplement Table 5 A–5 C. After SMR weighting, the majority of the covariates were well balanced with SMD less than 0·1 (Supplement Figs. 1A–10 C). The covariates with SMD larger than 0·1 were added in the corresponding models for doubly robust adjustment. The plots of scaled Schoenfeld residuals did not show clear evidence of violation of the proportional hazards assumption (Supplement Fig. 11A–11C).

**Fig. 3:**
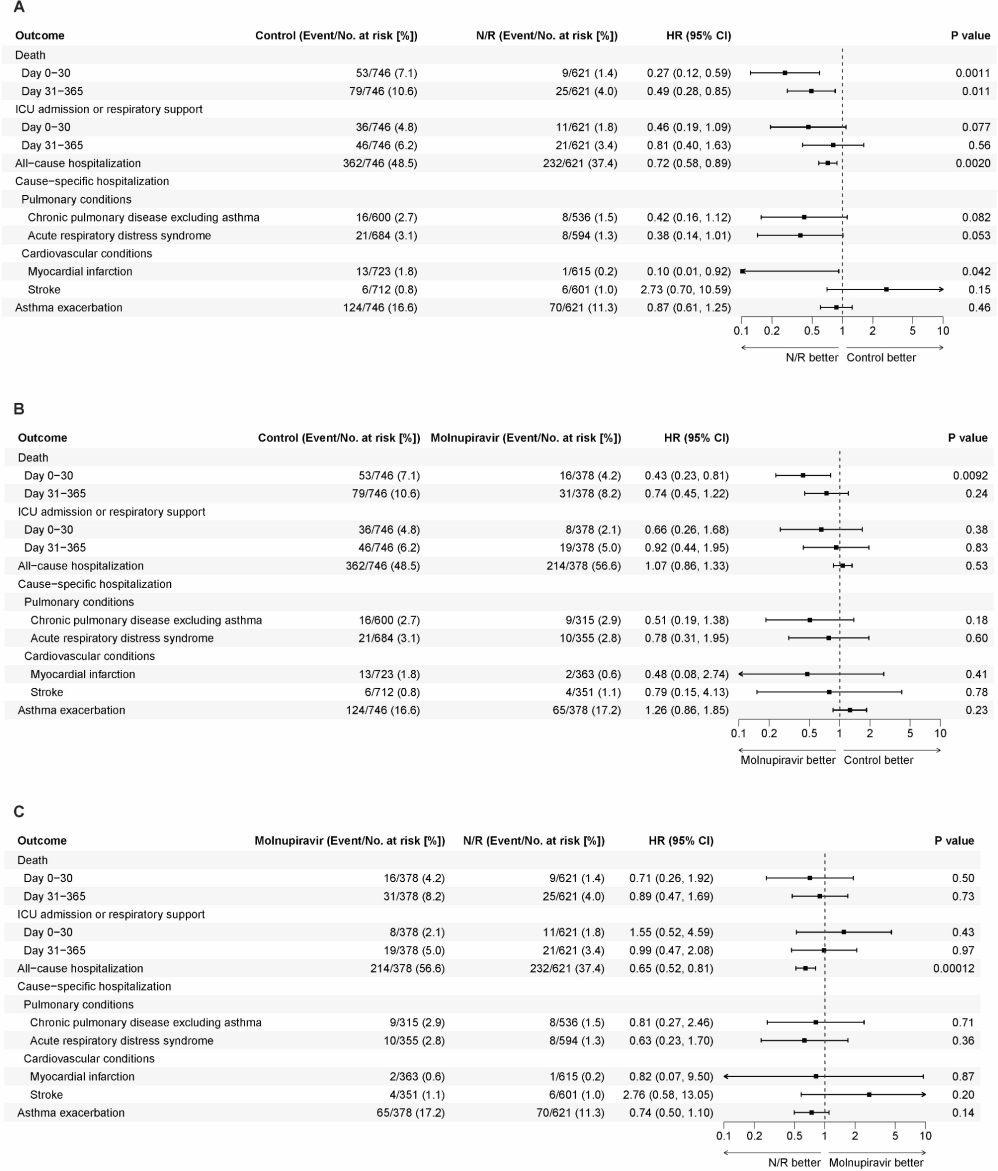
Effects of the antivirals on each outcome after weighting (the hazard ratios for the time intervals of Day 0–30 and Day 31–365 were derived from separate Cox proportional hazards models). Outcomes were ascertained from Day 31 to Day 365, unless otherwise specified. The axis is in logarithmic scale. (**A**): Comparison between the nirmatrelvir/ritonavir group and control group. (**B**): Comparison between the molnupiravir group and control group. (**C**): Comparison between the nirmatrelvir/ritonavir group and molnupiravir group. N/R: nirmatrelvir/ritonavir

In the comparison between the nirmatrelvir/ritonavir group and control group, the Cox models showed significantly lower risks of acute inpatient death (HR, 0·27 [95% CI, 0·12 to 0·59]; *p* = 0·0011), post-acute inpatient death (HR, 0·49 [95% CI, 0·28 to 0·85]; *p* = 0·011), all-cause hospitalization (HR, 0·72 [95% CI, 0·58 to 0·89]; *p* = 0·0020), and myocardial infarction (HR, 0·10 [95% CI, 0·01 to 0·92]; *p* = 0·042) in the nirmatrelvir/ritonavir group compared to the control group (Fig. 3A). The association between acute respiratory distress syndrome and nirmatrelvir/ritonavir was not significant (HR, 0·38 [95% CI, 0·14 to 1·01]; *p* = 0·053). In the comparison between the molnupiravir group and control group, patients in the molnupiravir group had a significantly lower hazard of acute inpatient death than those in the control group (HR, 0·43 [95% CI, 0·23 to 0·81]; *p* = 0·0092) (Fig. 3B). In the comparison between the nirmatrelvir/ritonavir group and molnupiravir group, the risk of post-acute all-cause hospitalization was significantly lower in the nirmatrelvir/ritonavir group than in the molnupiravir group (HR, 0·65 [95% CI, 0·52 to 0·81]; *p* = 0·00012) (Fig. 3C).

Some key findings of the subgroups analysis are noted (Supplement Figs. 12A–14C). In the comparisons between the nirmatrelvir/ritonavir group and control group as well as between the molnupiravir group and control group, patients who were prescribed no ICS or low-dose ICS, compared to patients who were prescribed medium- or high-dose ICS, had lower hazard ratios of post-acute inpatient death (nirmatrelvir/ritonavir: 0·33 vs. 0·80; molnupiravir: 0·67 vs. 0·90) and all-cause hospitalization (nirmatrelvir/ritonavir: 0·60 vs. 0·96; molnupiravir: 0·96 vs. 1·10). In the comparison between the nirmatrelvir group and molnupiravir group, among patients who were prescribed medium- or high-dose ICS, patients in the nirmatrelvir/ritonavir group had a lower hazard of asthma exacerbation than those in the molnupiravir group (HR, 0·58 [95% CI, 0·35 to 0·95]; *p* = 0.030). A similar finding on asthma exacerbation was observed among patients who were hospitalized on the index date (HR, 0·49 [95% CI, 0·29 to 0·84]; *p* = 0·0092). The sensitivity analysis showed similar results to the those from the primary analysis (Supplement Table 7, Supplement Figs. 15, 16 and 17).

## Discussion

There is limited research documenting the effectiveness of antivirals on the acute and post-acute clinical outcomes of SARS-CoV-2 infection specifically in patients with asthma. Our study examined the association of the COVID-19 antivirals with the acute and post-acute mortality, all-cause hospitalization, and hospitalizations due to several major diseases among patients with asthma in Hong Kong during the Omicron-dominant period. We primarily showed that asthma patients treated with nirmatrelvir/ritonavir were associated with lower risks of acute mortality, post-acute mortality, and all-cause hospitalization after SARS-CoV-2 infection, whereas those treated with molnupiravir only had a lower risk of acute mortality, compared to patients not receiving the corresponding antiviral treatment. Corroborated with a similar study, nirmatrelvir/ritonavir was more effective than molnupiravir in reducing the risk of 90-day mortality among patients with chronic respiratory diseases [19]. In addition, the effect size of decreased risk in acute mortality among nirmatrelvir/ritonavir recipients in our study is compatible to a study on patients with chronic respiratory diseases, on top of their demonstration of the acute benefit of molnupiravir [20]. Our result is different from Faust et al. [21], showing that there was no acute benefit of nirmatrelvir/ritonavir on the composite outcome of all-cause emergency department visits, hospitalization, and mortality, among patients with asthma or chronic obstructive pulmonary disease, likely due to a reduced sample size in their subgroup analysis [21].

In respect of post-acute outcomes, while no investigations were specifically designed for asthma patients, the reduced risks of nirmatrelvir/ritonavir in our study are compatible to that in studies using general outpatients and hospitalized patients [8–10]. Our study demonstrated the long-term benefit of nirmatrelvir/ritonavir on post-acute mortality in asthma patients, which is in line with Bajema et al. [8] showing a similar reduction in non-hospitalized patients. While they were unable to demonstrate the protective effect on post-acute hospitalization, we additionally showed that asthma patients treated with nirmatrelvir/ritonavir had significantly lower risks of post-acute hospitalization as well as hospitalization due to myocardial infarction, and a lower risk of hospitalization due to acute respiratory distress syndrome that was marginally significant. Given that nirmatrelvir/ritonavir is able to lower the risk of acute severity of COVID-19, it may thus mitigate the progression of myocardial infarction and acute respiratory distress syndrome, as two of the major complications of severe COVID-19 illness [33]. In contrast, we found that nirmatrelvir/ritonavir was associated with a lower risk of post-acute hospitalization compared to molnupiravir, and did not observe a statistically significant effect of molnupiravir on reducing post-acute outcomes, echoing that molnupiravir is generally regarded as less effective compared to nirmatrelvir/ritonavir. Nevertheless, Fung et al. [9] demonstrated a protective effect of molnupiravir on PCC, in spite of differences in study population and the definition of PCC. It should be noted that molnupiravir is suggested for prescription to patients with severe hepatic and renal impairment in Hong Kong due to the risk of hepatotoxicity and nephrotoxicity in patients receiving nirmatrelvir/ritonavir. Therefore, these patients may be more prone to experiencing adverse health conditions during the post-acute infection phase, potentially leading to an insignificant risk reduction in PCCs.

Our subgroup analysis additionally showed that both nirmatrelvir/ritonavir and molnupiravir were more effective in reducing post-acute mortality and hospitalization among mild asthma patients (prescribed no ICS or low-dose ICS) than among moderate-to-severe asthma patients (prescribed medium- or high-dose ICS). In many clinical management guidelines, patients with moderate-to-severe asthma are generally suggested to be prescribed nirmatrelvir/ritonavir or molnupiravir when infected with COVID-19. The long-term benefits demonstrated in our study thus suggest a potential consideration for including antiviral prescriptions among those with mild asthma. We also found that nirmatrelvir/ritonavir was associated with lower risks of asthma exacerbation compared to molnupiravir among patients with moderate-to-severe asthma (prescribed medium- or high-dose ICS) or relatively severe COVID-19 (hospitalized on the index date of COVID-19). Nevertheless, with smaller sample sizes in the subgroups, the findings warrant further investigation to validate.

The major strength of this investigation is that we used the territory-wide real-world data, covering all the COVID-19 cases with asthma from inpatient and outpatient settings to enhance the generalizability of the study findings. The healthcare records of all the COVID-19 cases, including medications, disease diagnoses, and vaccinations were digitally documented, and have been widely utilized in various investigations [2, 10, 13]. In addition, our study period has been restricted to cover almost all study participants infected by Omicron sub-lineages BA.2 and BA.5 [34], reducing potential variations from different SARS-CoV-2 variants such as Delta variant [35]. There are several limitations in this study. Firstly, the COVID-19 patients were mainly treated by nirmatrelvir/ritonavir and molnupiravir in Hong Kong during the Omicron epidemics. Other antivirals such as remdesivir were not studied due to a small number of samples. Secondly, several post-acute sequelae outcomes did not have enough number of cases, and this may introduce sparse data bias. Thirdly, we acknowledge the potential influence of residual confounding owing to the observational design. For example, the heterogeneity of healthcare-seeking behaviors between high and low economic groups likely affected their attendance at outpatient clinics for seeking antivirals.

In conclusion, our study demonstrated that asthma patients treated with nirmatrelvir/ritonavir were associated with lower risks of acute and post-acute outcomes after SARS-CoV-2 infection, while those treated with molnupiravir only had a lower risk of acute mortality. In addition, the post-acute benefits of the antivirals were also demonstrated in patients with mild asthma. Therefore, our findings suggested prescribing nirmatrelvir/ritonavir over molnupiravir in asthma patients who contract COVID-19, including those with mild asthma, if there is no feasibility concern.

## Electronic supplementary material

Below is the link to the electronic supplementary material.

Supplementary Material 1

Supplementary Material 2

## Data Availability

The cases’ surveillance data and medication records were extracted from electronic records in the system managed by the Hong Kong Hospital Authority. The vaccine history was extracted from the COVID-19 surveillance database provided by the Department of Health in Hong Kong. Restrictions apply to the availability of these data.
