# Supplementary material for: Short- and long-term comparative effectiveness of nirmatrelvir/ritonavir and molnupiravir in asthma patients: a cohort study
Source: Respir Res. 2025 Feb 28;26:75. doi: 10.1186/s12931-025-03156-2 (PMC11871785; doi:10.1186/s12931-025-03156-2)
Supplement: Supplementary file 1 — Supplementary Material 1 [file 12931_2025_3156_MOESM1_ESM.pdf]

Supplementary materials

Contents

Supplement Tables 5

Supplement Table 1. Asthma medications considered in this study 5

Supplement Table 2. ICD-9-CM codes relevant to outcomes 5

Supplement Table 3. ICD-9-CM codes for selected comorbidities 6

Supplement Table 4A. Patient characteristics before weighting with standardized mean differences between the nirmatrelvir-ritonavir group and the control group 7

Supplement Table 4B. Patient characteristics before weighting with standardized mean differences between the molnupiravir group and the control group 8

Supplement Table 4C. Patient characteristics before weighting with standardized mean differences between the nirmatrelvir-ritonavir group and the molnupiravir group 9

Supplement Table 5A. Patient characteristics after weighting with standardized mean differences between the nirmatrelvir-ritonavir group and the control group 10

Supplement Table 5B. Patient characteristics after weighting with standardized mean differences between the molnupiravir group and the control group 11

Supplement Table 5C. Patient characteristics after weighting with standardized mean differences between the nirmatrelvir-ritonavir group and the molnupiravir group 12

Supplement Table 6. Proportions of patients with moderate to severe COVID-19 at baseline based on prescription of concomitant pharmacological treatments 13

Supplement Table 7. Sensitivity analysis 1: Patient characteristics in the comparison between the molnupiravir group and control group with the start of the study period as the date when molnupiravir became available (Feb. 26, 2022) and without excluding individuals with contraindications to nirmatrelvir/ritonavir 14

Supplement Figures 15

Supplement Figure 1A. Standardized mean difference for each covariate in the comparison between the nirmatrelvir/ritonavir group and control group for the outcome of death (day 0–30) before and after weighting 15

Supplement Figure 1B. Standardized mean difference for each covariate in the comparison between the molnupiravir group and control group for the outcome of death (day 0–30) before and after weighting 16

Supplement Figure 1C. Standardized mean difference for each covariate in the comparison between the nirmatrelvir/ritonavir group and molnupiravir group for the outcome of death (day 0–30) before and after weighting 17

Supplement Figure 2A. Standardized mean difference for each covariate in the comparison between the nirmatrelvir/ritonavir group and control group for the outcome of death (day 31–365) before and after weighting 18

Supplement Figure 2B. Standardized mean difference for each covariate in the comparison between the molnupiravir group and control group for the outcome of death (day 31–365) before and after weighting 19

Supplement Figure 2C. Standardized mean difference for each covariate in the comparison between the nirmatrelvir/ritonavir group and molnupiravir group for the outcome of death (day 31–365) before and after weighting ... 20

Supplement Figure 3A. Standardized mean difference for each covariate in the comparison between the nirmatrelvir/ritonavir group and control group for the outcome of ICU admission or respiratory support (day 0–30) before and after weighting... 21

Supplement Figure 3B. Standardized mean difference for each covariate in the comparison between the molnupiravir group and control group for the outcome of ICU admission or respiratory support (day 0–30) before and after weighting ... 22

Supplement Figure 3C. Standardized mean difference for each covariate in the comparison between the nirmatrelvir/ritonavir group and molnupiravir group for the outcome of ICU admission or respiratory support (day 0–30) before and after weighting... 23

Supplement Figure 4A. Standardized mean difference for each covariate in the comparison between the nirmatrelvir/ritonavir group and control group for the outcome of ICU admission or respiratory support (day 31–365) before and after weighting... 24

Supplement Figure 4B. Standardized mean difference for each covariate in the comparison between the molnupiravir group and control group for the outcome of ICU admission or respiratory support (day 31–365) before and after weighting ... 25

Supplement Figure 4C. Standardized mean difference for each covariate in the comparison between the nirmatrelvir/ritonavir group and molnupiravir group for the outcome of ICU admission or respiratory support (day 31–365) before and after weighting... 26

Supplement Figure 5A. Standardized mean difference for each covariate in the comparison between the nirmatrelvir/ritonavir group and control group for the outcome of all-cause hospitalization before and after weighting ... 27

Supplement Figure 5B. Standardized mean difference for each covariate in the comparison between the molnupiravir group and control group for the outcome of all-cause hospitalization before and after weighting ... 28

Supplement Figure 5C. Standardized mean difference for each covariate in the comparison between the nirmatrelvir/ritonavir group and molnupiravir group for the outcome of all-cause hospitalization before and after weighting ... 29

Supplement Figure 6A. Standardized mean difference for each covariate in the comparison between the nirmatrelvir/ritonavir group and control group for the outcome of chronic pulmonary disease excluding asthma before and after weighting... 30

Supplement Figure 6B. Standardized mean difference for each covariate in the comparison between the molnupiravir group and control group for the outcome of chronic pulmonary disease excluding asthma before and after weighting ... 31

Supplement Figure 6C. Standardized mean difference for each covariate in the comparison between the nirmatrelvir/ritonavir group and molnupiravir group for the outcome of chronic pulmonary disease excluding asthma before and after weighting... 32

Supplement Figure 7A. Standardized mean difference for each covariate in the comparison between the nirmatrelvir/ritonavir group and control group for the outcome of acute respiratory distress syndrome before and after weighting... 33

Supplement Figure 7B. Standardized mean difference for each covariate in the comparison between the molnupiravir group and control group for the outcome of acute respiratory distress syndrome before and after weighting ... 34

Supplement Figure 7C. Standardized mean difference for each covariate in the comparison between the nirmatrelvir/ritonavir group and molnupiravir group for the outcome of acute respiratory distress syndrome before and after weighting 35

Supplement Figure 8A. Standardized mean difference for each covariate in the comparison between the nirmatrelvir/ritonavir group and control group for the outcome of myocardial infarction before and after weighting 36

Supplement Figure 8B. Standardized mean difference for each covariate in the comparison between the molnupiravir group and control group for the outcome of myocardial infarction before and after weighting 37

Supplement Figure 8C. Standardized mean difference for each covariate in the comparison between the nirmatrelvir/ritonavir group and molnupiravir group for the outcome of myocardial infarction before and after weighting 38

Supplement Figure 9A. Standardized mean difference for each covariate in the comparison between the nirmatrelvir/ritonavir group and control group for the outcome of stroke before and after weighting 39

Supplement Figure 9B. Standardized mean difference for each covariate in the comparison between the molnupiravir group and control group for the outcome of stroke before and after weighting 40

Supplement Figure 9C. Standardized mean difference for each covariate in the comparison between the nirmatrelvir/ritonavir group and molnupiravir group for the outcome of stroke before and after weighting 41

Supplement Figure 10A. Standardized mean difference for each covariate in the comparison between the nirmatrelvir/ritonavir group and control group for the outcome of asthma exacerbation before and after weighting 42

Supplement Figure 10B. Standardized mean difference for each covariate in the comparison between the molnupiravir group and control group for the outcome of asthma exacerbation before and after weighting 43

Supplement Figure 10C. Standardized mean difference for each covariate in the comparison between the nirmatrelvir/ritonavir group and molnupiravir group for the outcome of asthma exacerbation before and after weighting 44

Supplement Figure 11A. Scaled Schoenfeld residual plots for the comparison between the nirmatrelvir/ritonavir group and control group 45

Supplement Figure 11B. Scaled Schoenfeld residual plots for the comparison between the molnupiravir group and control group 46

Supplement Figure 11C. Scaled Schoenfeld residual plots for the comparison between the nirmatrelvir/ritonavir group and molnupiravir group 47

Supplement Figure 12A. Subgroup analysis: vaccination status in the comparison between the nirmatrelvir/ritonavir group and control group 48

Supplement Figure 12B. Subgroup analysis: vaccination status in the comparison between the molnupiravir group and control group 49

Supplement Figure 12C. Subgroup analysis: vaccination status in the comparison between the nirmatrelvir/ritonavir group and molnupiravir group 50

Supplement Figure 13A. Subgroup analysis: inhaled corticosteroid (ICS) dose level in the comparison between the nirmatrelvir/ritonavir group and control group 51

Supplement Figure 13B. Subgroup analysis: inhaled corticosteroid (ICS) dose level in the comparison between the molnupiravir group and control group 52

Supplement Figure 13C. Subgroup analysis: inhaled corticosteroid (ICS) dose level in the comparison between the nirmatrelvir/ritonavir group and molnupiravir group 53

Supplement Figure 14A. Subgroup analysis: hospital admission on index date in the comparison between the nirmatrelvir/ritonavir group and control group............................ 54

Supplement Figure 14B. Subgroup analysis: hospital admission on index date in the comparison between the molnupiravir group and control group.. 55

Supplement Figure 14C. Subgroup analysis: hospital admission on index date in the comparison between the nirmatrelvir/ritonavir group and molnupiravir group 56

Supplement Figure 15. Sensitivity analysis 1: Comparison between the molnupiravir group and control group with the start of the study period as the date when molnupiravir became available (Feb. 26, 2022) and without excluding individuals with contraindications to nirmatrelvir/ritonavir 57

Supplement Figure 16. Sensitivity analysis 2: Comparison between the nirmatrelvir/ritonavir group and control group excluding patients who were prescribed salmeterol within 10 days of the index date 58

Supplement Figure 17. Sensitivity analysis 3: Comparison between the nirmatrelvir/ritonavir group and molnupiravir group excluding patients who were prescribed salmeterol within 10 days of the index date.. 59

References 60

Supplement Tables

Supplement Table 1. Asthma medications considered in this study [1]

Short-acting $beta_2$ agonist (SABA)	salbutamol, terbutaline
Inhaled corticosteroid (ICS) alone or in combination with LABA or LAMA	atectura, beclomethasone, budesonide, budesonide + formoterol, ciclesonide, enerzair, fluticasone, flutiform, relvar ellipta, salmeterol xinafoate + fluticasone propionate, trelegy ellipta
Others	aminophylline, ipratropium, montelukast, salmeterol, tiotropium, theophylline

LABA: long-acting $beta_2$ agonist. LAMA: long-acting muscarinic antagonist.

Supplement Table 2. ICD-9-CM codes relevant to outcomes [2]

Conditions	ICD-9-CM codes
Respiratory support	39.65, 93.90, 93.95, 93.96, 96.04, 96.7x
Chronic pulmonary disease excluding asthma	416.8, 416.9, 490.x–492.x, 494–505.x, 506.4, 508.1, 508.8
Acute respiratory distress syndrome	518.51, 518.52, 518.53, 518.8
Myocardial infarction	410
Stroke	430–437.x

Supplement Table 3. ICD-9-CM codes for selected comorbidities [3]

Comorbidity	ICD-9-CM code
Arrhythmia	426.0, 426.13, 426.7, 426.9, 426.10, 426.12, 427.0, 427.1, 427.2, 427.3, 427.4, 427.6, 427.7, 427.8, 427.9, 785.0, 996.01, 996.04, V45.0, V53.3
Cerebrovascular disease	362.34, 430.x-438.x
Chronic obstructive pulmonary disease	496
Congestive heart failure	398.91, 402.01, 402.11, 402.91, 404.01, 404.03, 404.11, 404.13, 404.91, 404.93, 425.4-425.9, 428.x
Coronary artery disease	410.x-414.x, V45.81
Diabetes	250.x
Hypertension	401.x-405.x
Liver disease	070.22, 070.23, 070.32, 070.33, 070.44, 070.54, 070.6, 070.9, 456.0-456.2, 570.x, 571.x, 572.2-572.8, 573.3, 573.4, 573.8, 573.9, V42.7
Malignancy	140.x–172.x, 174.x–208.x, 238.6
Renal disease	403.01, 403.11, 403.91, 404.02, 404.03, 404.12, 404.13, 404.92, 404.93, 582.x, 583.0-583.7, 585.x, 586.x, 588.0, V42.0, V45.1, V56.x

Supplement Table 4A. Patient characteristics before weighting with standardized mean differences between the nirmatrelvir-ritonavir group and the control group

	Control	N/R	SMD
N	746	621	
Age (median [IQR])	72.00 [50.00, 85.00]	70.00 [55.00, 80.00]	0.01
Sex			
Female (%)	431 (57.8)	384 (61.8)	0.08
Male (%)	315 (42.2)	237 (38.2)	0.08
Vaccination status (%)			0.44
Unvaccinated	165 (22.1)	73 (11.8)	
1-2 doses	222 (29.8)	118 (19.0)	
3 or more doses	359 (48.1)	430 (69.2)	
Hospital admission (%)	336 (45.0)	241 (38.8)	0.13
Concomitant pharmacological treatments			
Dexamethasone (%)	147 (19.7)	28 (4.5)	0.48
Prednisolone (%)	49 (6.6)	36 (5.8)	0.03
Remdesivir (%)	134 (18.0)	12 (1.9)	0.56
Comorbidity			
Arrhythmia (%)	71 (9.5)	29 (4.7)	0.19
Cerebrovascular disease (%)	57 (7.6)	25 (4.0)	0.16
Chronic obstructive pulmonary disease (%)	106 (14.2)	55 (8.9)	0.17
Congestive heart failure (%)	104 (13.9)	31 (5.0)	0.31
Coronary artery disease (%)	86 (11.5)	36 (5.8)	0.21
Diabetes (%)	94 (12.6)	47 (7.6)	0.17
Hypertension (%)	179 (24.0)	109 (17.6)	0.16
Liver disease (%)	31 (4.2)	15 (2.4)	0.10
Malignancy (%)	40 (5.4)	26 (4.2)	0.06
Renal disease (%)	12 (1.6)	5 (0.8)	0.07
Recent use of an oral glucocorticoid (%)	223 (29.9)	108 (17.4)	0.30
ICS dose (%)			0.12
None	258 (34.6)	239 (38.5)	
Low	147 (19.7)	133 (21.4)	
Medium	252 (33.8)	186 (30.0)	
High	89 (11.9)	63 (10.1)	

N/R: nirmatrelvir-ritonavir. SMD: standardized mean difference.

Supplement Table 4B. Patient characteristics before weighting with standardized mean differences between the molnupiravir group and the control group

	Control	Molnupiravir	SMD
N	746	378	
Age (median [IQR])	72.00 [50.00, 85.00]	77.00 [67.00, 86.00]	0.42
Sex			
Female (%)	431 (57.8)	227 (60.1)	0.05
Male (%)	315 (42.2)	151 (39.9)	0.05
Vaccination status (%)			0.24
Unvaccinated	165 (22.1)	71 (18.8)	
1-2 doses	222 (29.8)	82 (21.7)	
3 or more doses	359 (48.1)	225 (59.5)	
Hospital admission (%)	336 (45.0)	156 (41.3)	0.08
Concomitant pharmacological treatments			
Dexamethasone (%)	147 (19.7)	11 (2.9)	0.55
Prednisolone (%)	49 (6.6)	28 (7.4)	0.03
Remdesivir (%)	134 (18.0)	5 (1.3)	0.59
Comorbidity			
Arrhythmia (%)	71 (9.5)	75 (19.8)	0.30
Cerebrovascular disease (%)	57 (7.6)	46 (12.2)	0.15
Chronic obstructive pulmonary disease (%)	106 (14.2)	40 (10.6)	0.11
Congestive heart failure (%)	104 (13.9)	58 (15.3)	0.04
Coronary artery disease (%)	86 (11.5)	46 (12.2)	0.02
Diabetes (%)	94 (12.6)	57 (15.1)	0.07
Hypertension (%)	179 (24.0)	114 (30.2)	0.14
Liver disease (%)	31 (4.2)	23 (6.1)	0.09
Malignancy (%)	40 (5.4)	33 (8.7)	0.13
Renal disease (%)	12 (1.6)	22 (5.8)	0.22
Recent use of an oral glucocorticoid (%)	223 (29.9)	106 (28.0)	0.04
ICS dose (%)			0.21
None	258 (34.6)	109 (28.8)	
Low	147 (19.7)	71 (18.8)	
Medium	252 (33.8)	127 (33.6)	
High	89 (11.9)	71 (18.8)	

SMD: standardized mean difference.

Supplement Table 4C. Patient characteristics before weighting with standardized mean differences between the nirmatrelvir-ritonavir group and the molnupiravir group

	Molnupiravir	N/R	SMD
N	378	621	
Age (median [IQR])	77.00 [67.00, 86.00]	70.00 [55.00, 80.00]	0.47
Sex			
Female (%)	227 (60.1)	384 (61.8)	0.04
Male (%)	151 (39.9)	237 (38.2)	0.04
Vaccination status (%)			0.23
Unvaccinated	71 (18.8)	73 (11.8)	
1-2 doses	82 (21.7)	118 (19.0)	
3 or more doses	225 (59.5)	430 (69.2)	
Hospital admission (%)	156 (41.3)	241 (38.8)	0.05
Concomitant pharmacological treatments			
Dexamethasone (%)	11 (2.9)	28 (4.5)	0.09
Prednisolone (%)	28 (7.4)	36 (5.8)	0.07
Remdesivir (%)	5 (1.3)	12 (1.9)	0.05
Comorbidity			
Arrhythmia (%)	75 (19.8)	29 (4.7)	0.48
Cerebrovascular disease (%)	46 (12.2)	25 (4.0)	0.30
Chronic obstructive pulmonary disease (%)	40 (10.6)	55 (8.9)	0.06
Congestive heart failure (%)	58 (15.3)	31 (5.0)	0.35
Coronary artery disease (%)	46 (12.2)	36 (5.8)	0.22
Diabetes (%)	57 (15.1)	47 (7.6)	0.24
Hypertension (%)	114 (30.2)	109 (17.6)	0.30
Liver disease (%)	23 (6.1)	15 (2.4)	0.18
Malignancy (%)	33 (8.7)	26 (4.2)	0.19
Renal disease (%)	22 (5.8)	5 (0.8)	0.28
Recent use of an oral glucocorticoid (%)	106 (28.0)	108 (17.4)	0.26
ICS dose (%)			0.30
None	109 (28.8)	239 (38.5)	
Low	71 (18.8)	133 (21.4)	
Medium	127 (33.6)	186 (30.0)	
High	71 (18.8)	63 (10.1)	

N/R: nirmatrelvir-ritonavir. SMD: standardized mean difference.

Supplement Table 5A. Patient characteristics after weighting with standardized mean differences between the nirmatrelvir-ritonavir group and the control group

	Control	N/R	SMD
N	681.1	621.0	
Age (median [IQR])	72.00 [51.00, 85.00]	70.00 [54.25, 80.00]	0.07
Sex			
Female (%)	432.6 (63.5)	384.0 (61.8)	0.04
Male (%)	248.5 (36.5)	237.0 (38.2)	0.04
Vaccination status (%)			0.07
Unvaccinated	67.6 (9.9)	73.0 (11.8)	
1-2 doses	123.4 (18.1)	118.0 (19.0)	
3 or more doses	490.0 (71.9)	430.0 (69.2)	
Hospital admission (%)	288.7 (42.4)	241.0 (38.8)	0.07
Concomitant pharmacological treatments			
Dexamethasone (%)	34.2 (5.0)	28.0 (4.5)	0.02
Prednisolone (%)	39.5 (5.8)	36.0 (5.8)	0.00
Remdesivir (%)	12.8 (1.9)	12.0 (1.9)	0.00
Comorbidity			
Arrhythmia (%)	26.4 (3.9)	29.0 (4.7)	0.04
Cerebrovascular disease (%)	29.3 (4.3)	25.0 (4.0)	0.01
Chronic obstructive pulmonary disease (%)	67.1 (9.8)	55.0 (8.9)	0.03
Congestive heart failure (%)	33.4 (4.9)	31.0 (5.0)	0.00
Coronary artery disease (%)	44.0 (6.5)	36.0 (5.8)	0.03
Diabetes (%)	55.5 (8.2)	47.0 (7.6)	0.02
Hypertension (%)	146.8 (21.5)	109.0 (17.6)	0.10
Liver disease (%)	14.1 (2.1)	15.0 (2.4)	0.02
Malignancy (%)	32.1 (4.7)	26.0 (4.2)	0.03
Renal disease (%)	6.1 (0.9)	5.0 (0.8)	0.01
Recent use of an oral glucocorticoid (%)	120.7 (17.7)	108.0 (17.4)	0.01
ICS dose (%)			0.07
None	240.6 (35.3)	239.0 (38.5)	
Low	152.0 (22.3)	133.0 (21.4)	
Medium	217.4 (31.9)	186.0 (30.0)	
High	71.1 (10.4)	63.0 (10.1)	

N/R: nirmatrelvir-ritonavir. SMD: standardized mean difference.

Supplement Table 5B. Patient characteristics after weighting with standardized mean differences between the molnupiravir group and the control group

	Control	Molnupiravir	SMD
N	390.4	378.0	
Age (median [IQR])	82.00 [66.00, 90.00]	77.00 [67.00, 86.00]	0.11
Sex			
Female (%)	247.4 (63.4)	227.0 (60.1)	0.07
Male (%)	143.0 (36.6)	151.0 (39.9)	0.07
Vaccination status (%)			0.02
Unvaccinated	69.9 (17.9)	71.0 (18.8)	
1-2 doses	85.6 (21.9)	82.0 (21.7)	
3 or more doses	234.9 (60.2)	225.0 (59.5)	
Hospital admission (%)	164.9 (42.2)	156.0 (41.3)	0.02
Concomitant pharmacological treatments			
Dexamethasone (%)	12.1 (3.1)	11.0 (2.9)	0.01
Prednisolone (%)	25.8 (6.6)	28.0 (7.4)	0.03
Remdesivir (%)	5.1 (1.3)	5.0 (1.3)	0.00
Comorbidity			
Arrhythmia (%)	65.2 (16.7)	75.0 (19.8)	0.08
Cerebrovascular disease (%)	58.2 (14.9)	46.0 (12.2)	0.08
Chronic obstructive pulmonary disease (%)	47.2 (12.1)	40.0 (10.6)	0.05
Congestive heart failure (%)	63.2 (16.2)	58.0 (15.3)	0.02
Coronary artery disease (%)	44.0 (11.3)	46.0 (12.2)	0.03
Diabetes (%)	69.5 (17.8)	57.0 (15.1)	0.07
Hypertension (%)	138.1 (35.4)	114.0 (30.2)	0.11
Liver disease (%)	29.6 (7.6)	23.0 (6.1)	0.06
Malignancy (%)	33.2 (8.5)	33.0 (8.7)	0.01
Renal disease (%)	34.7 (8.9)	22.0 (5.8)	0.12
Recent use of an oral glucocorticoid (%)	129.4 (33.1)	106.0 (28.0)	0.11
ICS dose (%)			0.08
None	101.7 (26.0)	109.0 (28.8)	
Low	71.8 (18.4)	71.0 (18.8)	
Medium	145.3 (37.2)	127.0 (33.6)	
High	71.6 (18.4)	71.0 (18.8)	

SMD: standardized mean difference.

Supplement Table 5C. Patient characteristics after weighting with standardized mean differences between the nirmatrelvir-ritonavir group and the molnupiravir group

	Molnupiravir	N/R	SMD
N	620.6	621.0	
Age (median [IQR])	70.00 [56.56, 80.00]	70.00 [54.25, 80.00]	0.01
Sex			
Female (%)	388.7 (62.6)	384.0 (61.8)	0.02
Male (%)	231.9 (37.4)	237.0 (38.2)	0.02
Vaccination status (%)			0.07
Unvaccinated	70.5 (11.4)	73.0 (11.8)	
1-2 doses	135.5 (21.8)	118.0 (19.0)	
3 or more doses	414.6 (66.8)	430.0 (69.2)	
Hospital admission (%)	260.6 (42.0)	241.0 (38.8)	0.07
Concomitant pharmacological treatments			
Dexamethasone (%)	24.9 (4.0)	28.0 (4.5)	0.02
Prednisolone (%)	31.9 (5.1)	36.0 (5.8)	0.03
Remdesivir (%)	10.3 (1.7)	12.0 (1.9)	0.02
Comorbidity			
Arrhythmia (%)	28.0 (4.5)	29.0 (4.7)	0.01
Cerebrovascular disease (%)	35.3 (5.7)	25.0 (4.0)	0.08
Chronic obstructive pulmonary disease (%)	51.3 (8.3)	55.0 (8.9)	0.02
Congestive heart failure (%)	29.0 (4.7)	31.0 (5.0)	0.02
Coronary artery disease (%)	31.8 (5.1)	36.0 (5.8)	0.03
Diabetes (%)	58.1 (9.4)	47.0 (7.6)	0.06
Hypertension (%)	122.7 (19.8)	109.0 (17.6)	0.06
Liver disease (%)	18.2 (2.9)	15.0 (2.4)	0.03
Malignancy (%)	28.8 (4.6)	26.0 (4.2)	0.02
Renal disease (%)	5.2 (0.8)	5.0 (0.8)	0.00
Recent use of an oral glucocorticoid (%)	113.4 (18.3)	108.0 (17.4)	0.02
ICS dose (%)			0.04
None	248.9 (40.1)	239.0 (38.5)	
Low	124.1 (20.0)	133.0 (21.4)	
Medium	187.9 (30.3)	186.0 (30.0)	
High	59.6 (9.6)	63.0 (10.1)	

N/R: nirmatrelvir-ritonavir. SMD: standardized mean difference.

Supplement Table 6. Proportions of patients with moderate to severe COVID-19 at baseline based on prescription of concomitant pharmacological treatments

Outcome	N/R	Molnupiravir	Control
Death (day 0-30)	62/621 (10.0)	40/378 (10.6)	218/746 (29.2)
Death (day 31-365)	62/621 (10.0)	40/378 (10.6)	218/746 (29.2)
ICU admission or respiratory support (day 0-30)	62/621 (10.0)	40/378 (10.6)	218/746 (29.2)
ICU admission or respiratory support (day 31-365)	62/621 (10.0)	40/378 (10.6)	218/746 (29.2)
All-cause hospitalization	62/621 (10.0)	40/378 (10.6)	218/746 (29.2)
Chronic pulmonary disease excluding asthma	40/536 (7.5)	29/315 (9.2)	147/600 (24.5)
Acute respiratory distress syndrome	55/594 (9.3)	34/355 (9.6)	188/684 (27.5)
Myocardial infarction	62/615 (10.1)	37/363 (10.2)	207/723 (28.6)
Stroke	59/601 (9.8)	39/351 (11.1)	208/712 (29.2)
Asthma exacerbation	62/621 (10.0)	40/378 (10.6)	218/746 (29.2)

Patients were categorized into mild cases or moderate to severe cases based on prescription of concomitant pharmacological treatments. Patients who were not prescribed concomitant pharmacological treatments (i.e., dexamethasone, prednisolone, and remdesivir) were categorized as mild cases, while patients who were prescribed any of the concomitant treatments were categorized as moderate to severe cases. N/R: nirmatrelvir-ritonavir.

Supplement Table 7. Sensitivity analysis 1: Patient characteristics in the comparison between the molnupiravir group and control group with the start of the study period as the date when molnupiravir became available (Feb. 26, 2022) and without excluding individuals with contraindications to nirmatrelvir/ritonavir

Characteristic	Molnupiravir Group	Control Group
n	460	1146
Age (median [IQR])	78.00 [68.75, 87.00]	74.00 [55.00, 86.00]
Sex		
Female (%)	272 (59.1)	649 (56.6)
Male (%)	188 (40.9)	497 (43.4)
Vaccination status (%)		
Unvaccinated	104 (22.6)	369 (32.2)
1-2 doses	101 (22.0)	385 (33.6)
≥3 doses	255 (55.4)	392 (34.2)
Hospital admission (%)	198 (43.0)	489 (42.7)
Concomitant pharmacological treatments		
Dexamethasone (%)	26 (5.7)	220 (19.2)
Prednisolone (%)	34 (7.4)	71 (6.2)
Remdesivir (%)	8 (1.7)	156 (13.6)
Comorbidity		
Arrhythmia (%)	94 (20.4)	143 (12.5)
Cerebrovascular disease (%)	59 (12.8)	98 (8.6)
Chronic obstructive pulmonary disease (%)	54 (11.7)	184 (16.1)
Congestive heart failure (%)	78 (17.0)	186 (16.2)
Coronary artery disease (%)	59 (12.8)	130 (11.3)
Diabetes (%)	72 (15.7)	171 (14.9)
Hypertension (%)	154 (33.5)	329 (28.7)
Liver disease (%)	30 (6.5)	62 (5.4)
Malignancy (%)	37 (8.0)	70 (6.1)
Renal disease (%)	32 (7.0)	44 (3.8)
Recent use of an oral glucocorticoid (%)	131 (28.5)	384 (33.5)
ICS dose (%)		
None	122 (26.5)	358 (31.2)
Low	95 (20.7)	226 (19.7)
Medium	166 (36.1)	408 (35.6)
High	77 (16.7)	154 (13.4)

Supplement Figures

Supplement Figure 1A. Standardized mean difference for each covariate in the comparison between the nirmatrelvir/ritonavir group and control group for the outcome of death (day 0–30) before and after weighting

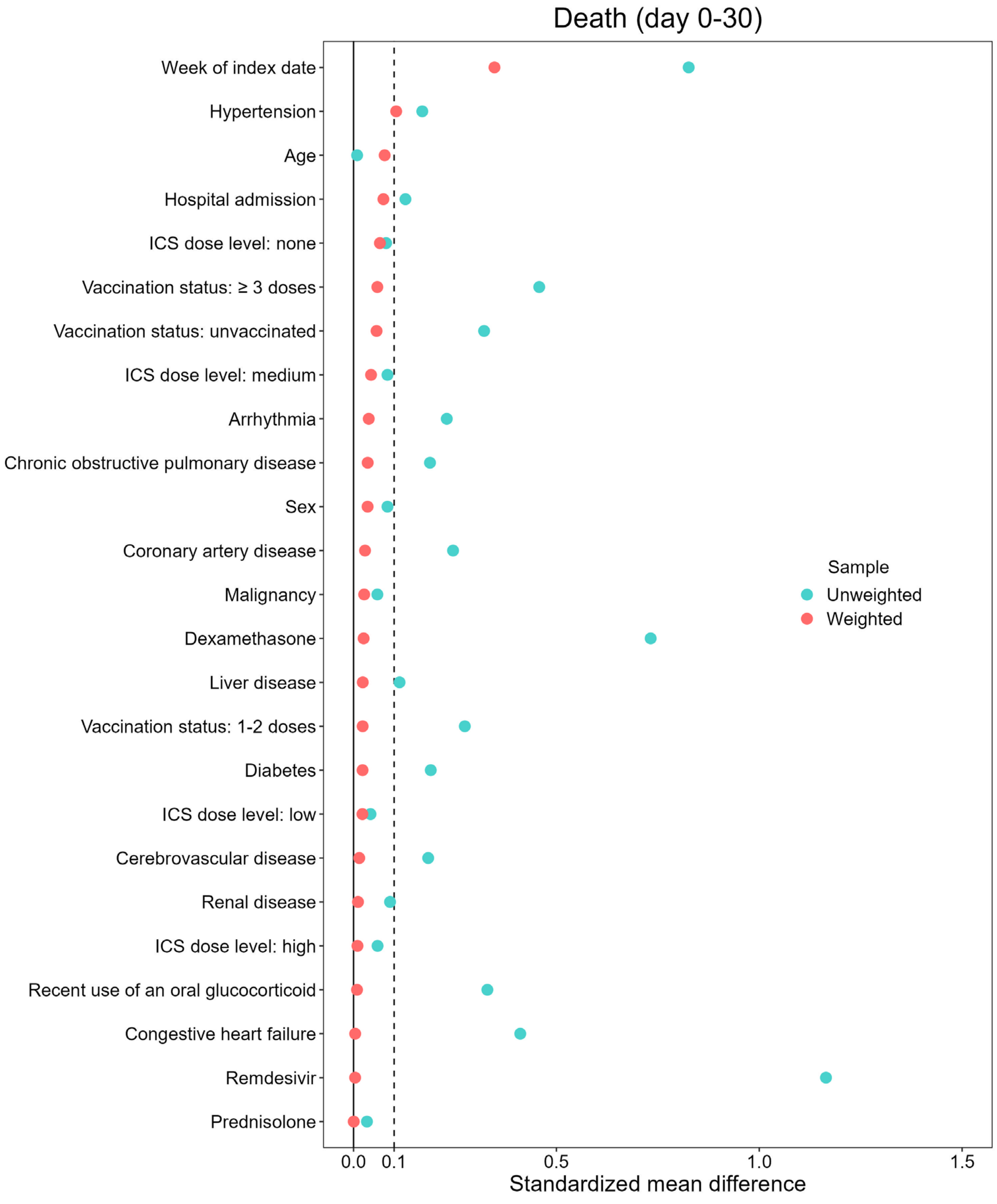

Supplement Figure 1B. Standardized mean difference for each covariate in the comparison between the molnupiravir group and control group for the outcome of death (day 0–30) before and after weighting

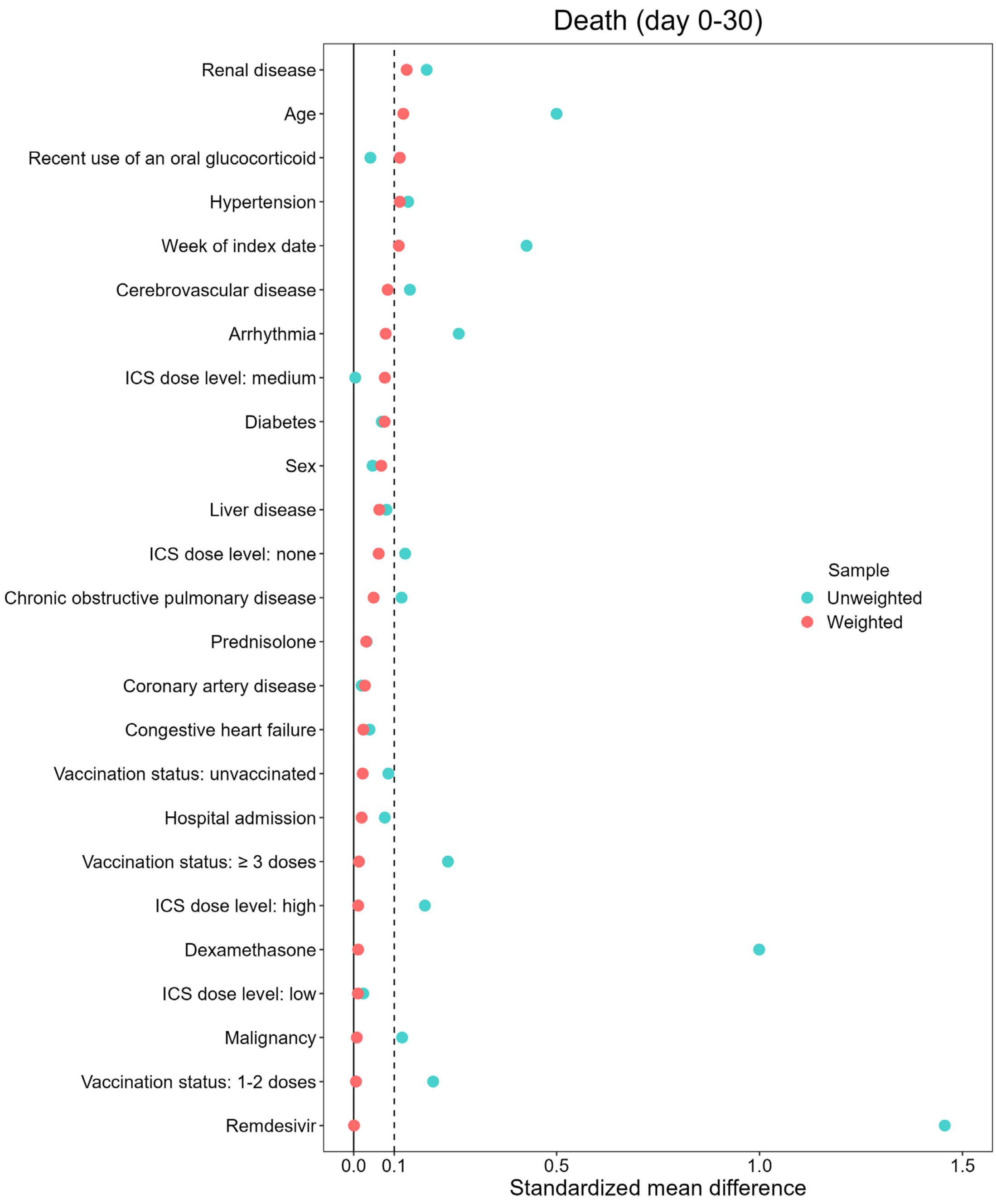

Supplement Figure 1C. Standardized mean difference for each covariate in the comparison between the nirmatrelvir/ritonavir group and molnupiravir group for the outcome of death (day 0–30) before and after weighting

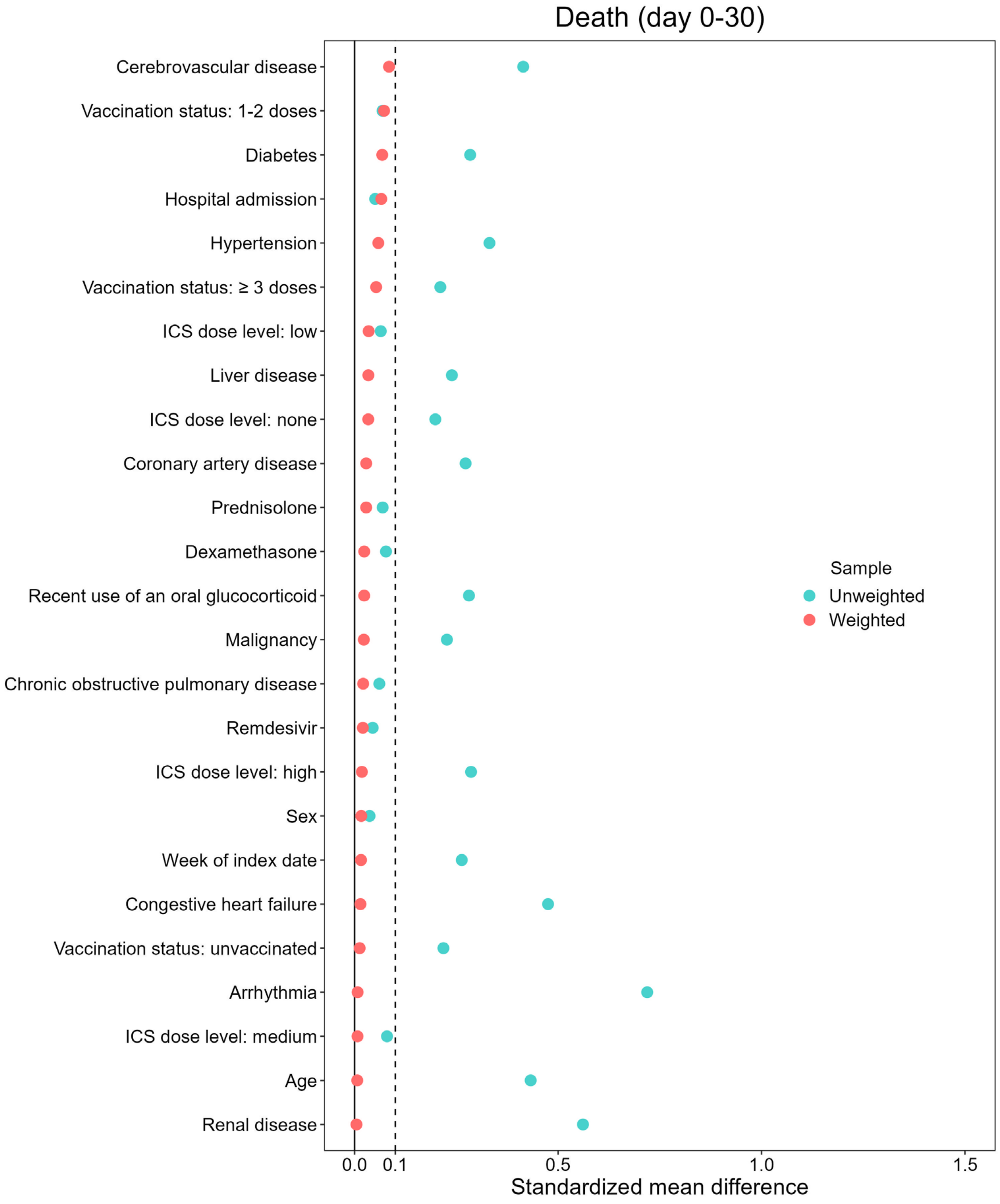

Supplement Figure 2A. Standardized mean difference for each covariate in the comparison between the nirmatrelvir/ritonavir group and control group for the outcome of death (day 31–365) before and after weighting

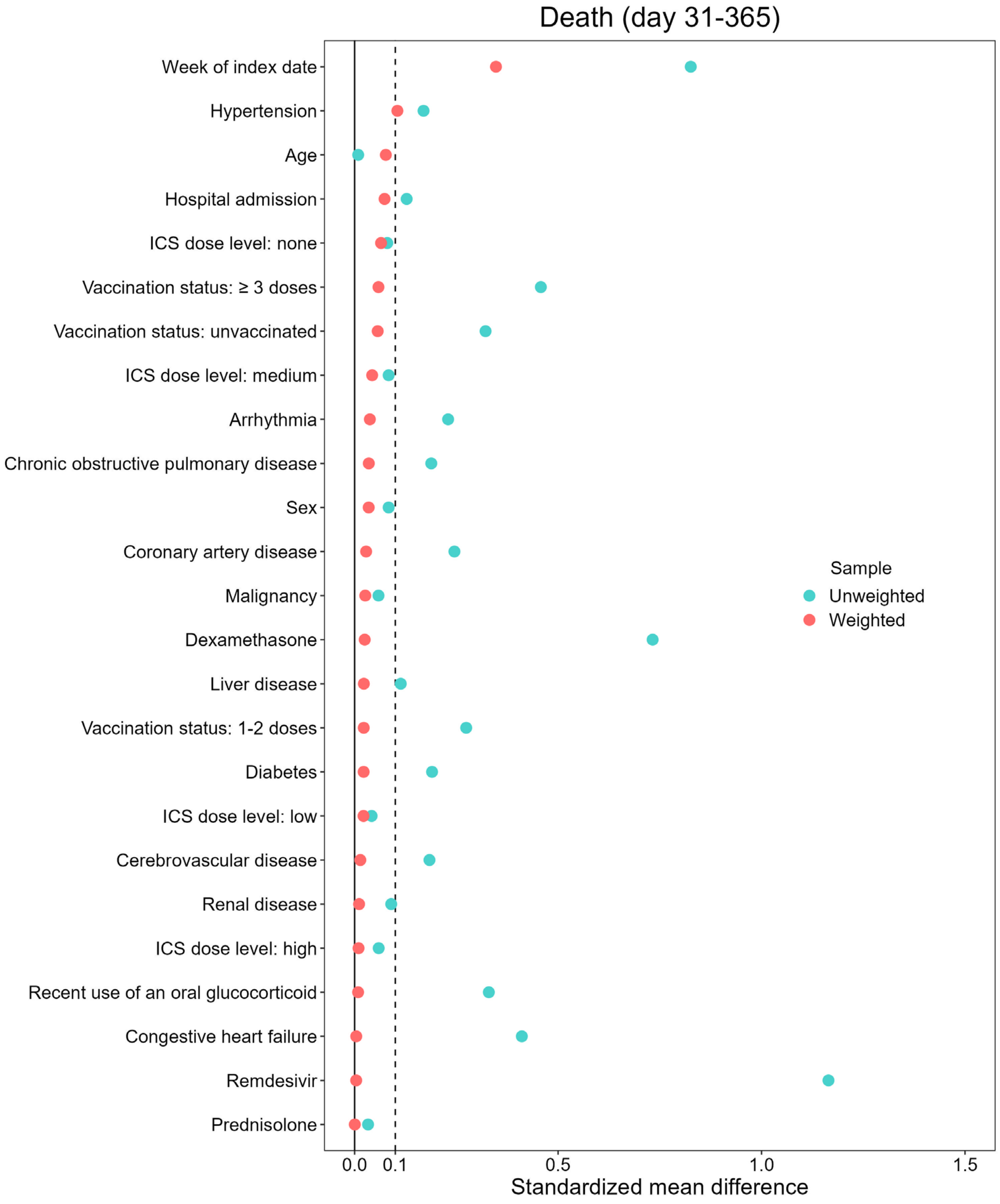

Supplement Figure 2B. Standardized mean difference for each covariate in the comparison between the molnupiravir group and control group for the outcome of death (day 31–365) before and after weighting

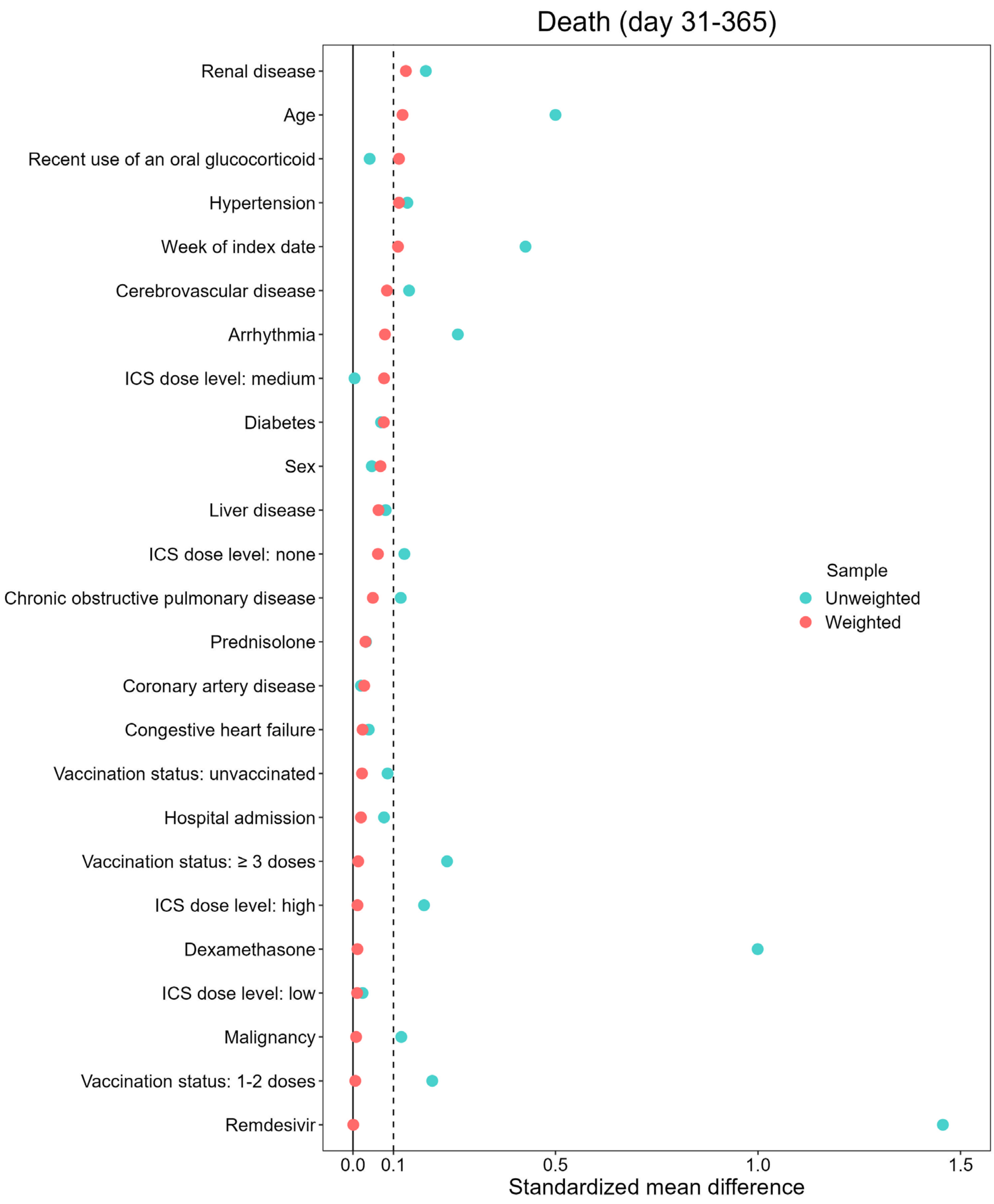

Supplement Figure 2C. Standardized mean difference for each covariate in the comparison between the nirmatrelvir/ritonavir group and molnupiravir group for the outcome of death (day 31–365) before and after weighting

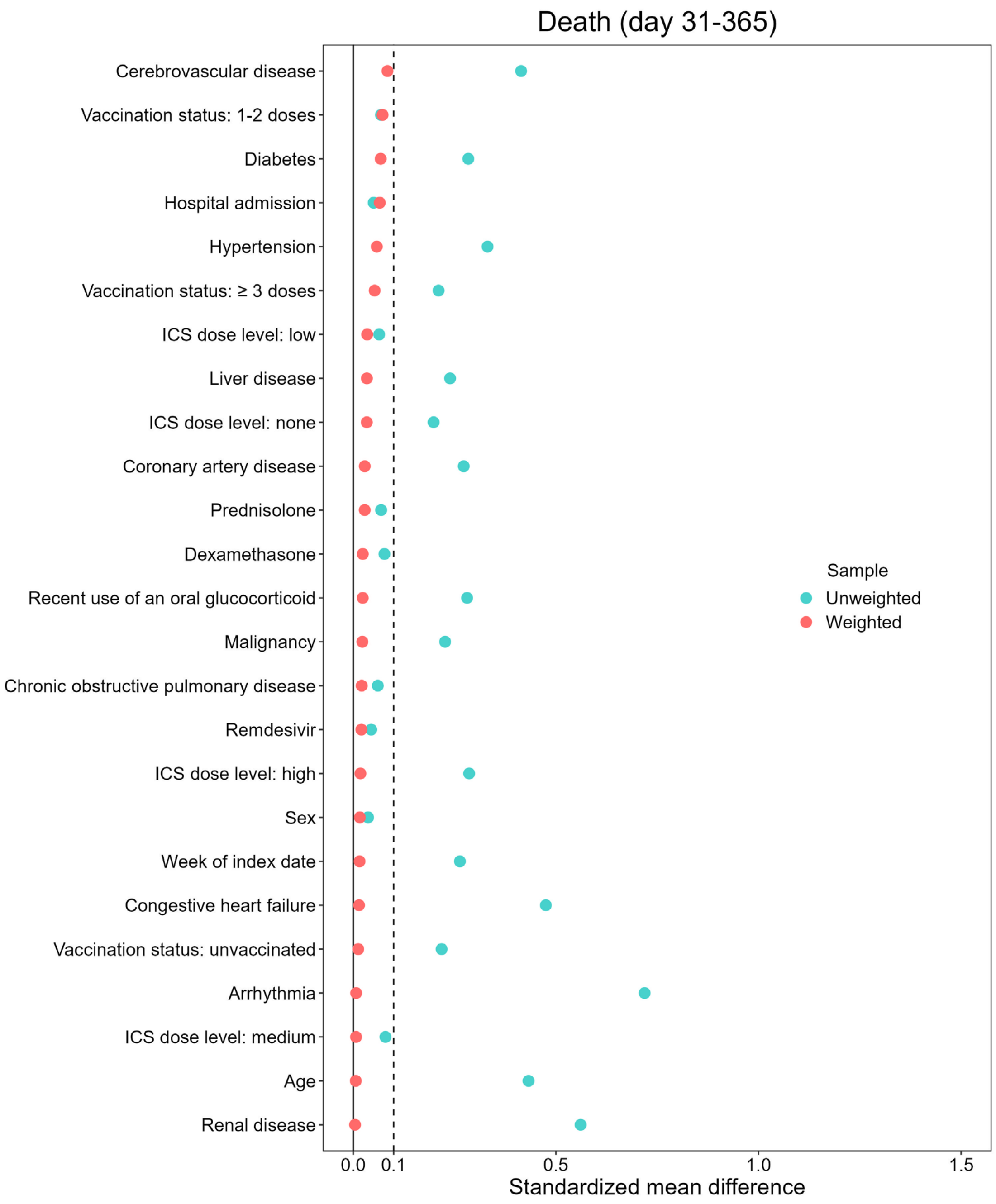

Supplement Figure 3A. Standardized mean difference for each covariate in the comparison between the nirmatrelvir/ritonavir group and control group for the outcome of ICU admission or respiratory support (day 0–30) before and after weighting

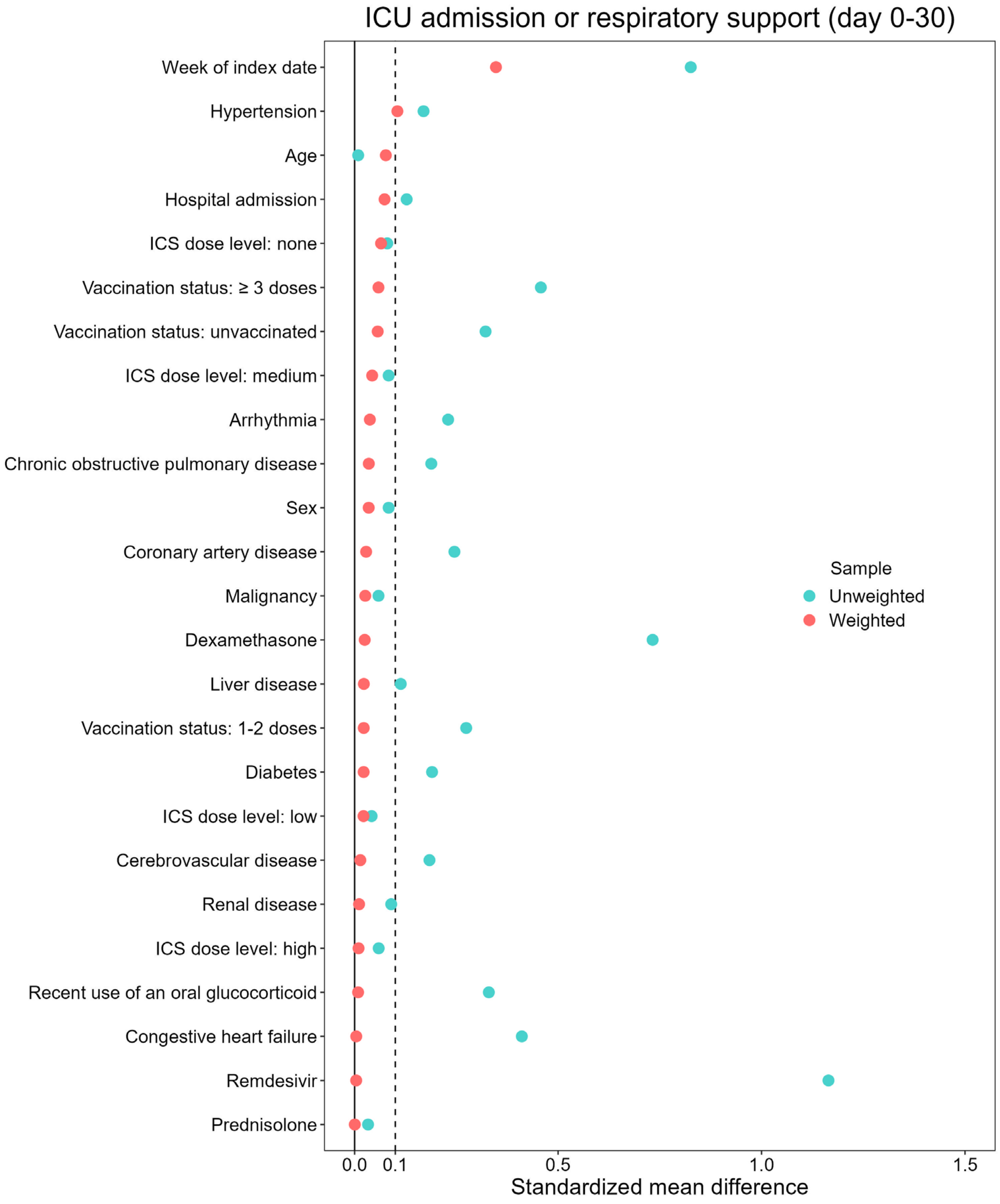

Supplement Figure 3B. Standardized mean difference for each covariate in the comparison between the molnupiravir group and control group for the outcome of ICU admission or respiratory support (day 0–30) before and after weighting

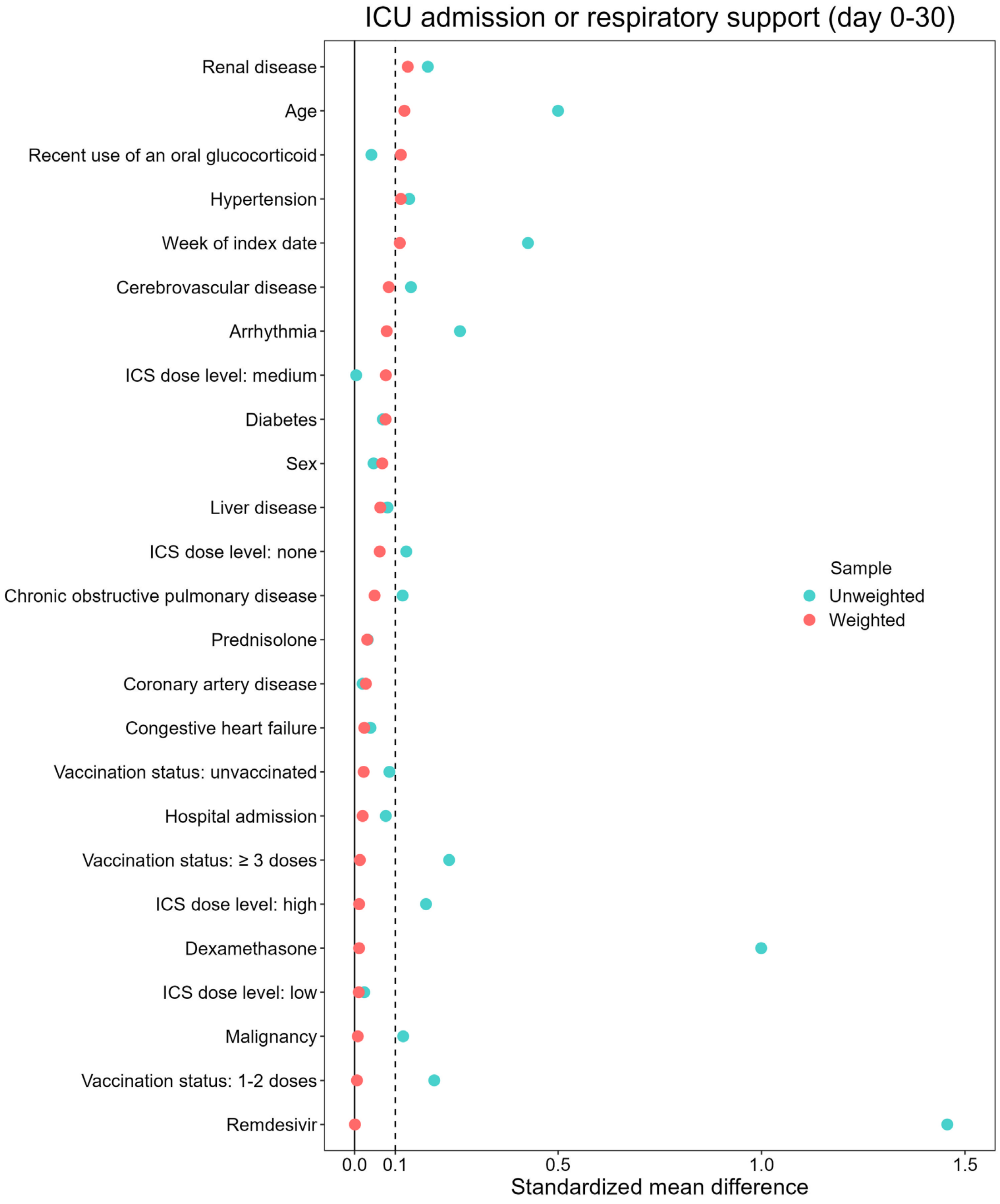

Supplement Figure 3C. Standardized mean difference for each covariate in the comparison between the nirmatrelvir/ritonavir group and molnupiravir group for the outcome of ICU admission or respiratory support (day 0–30) before and after weighting

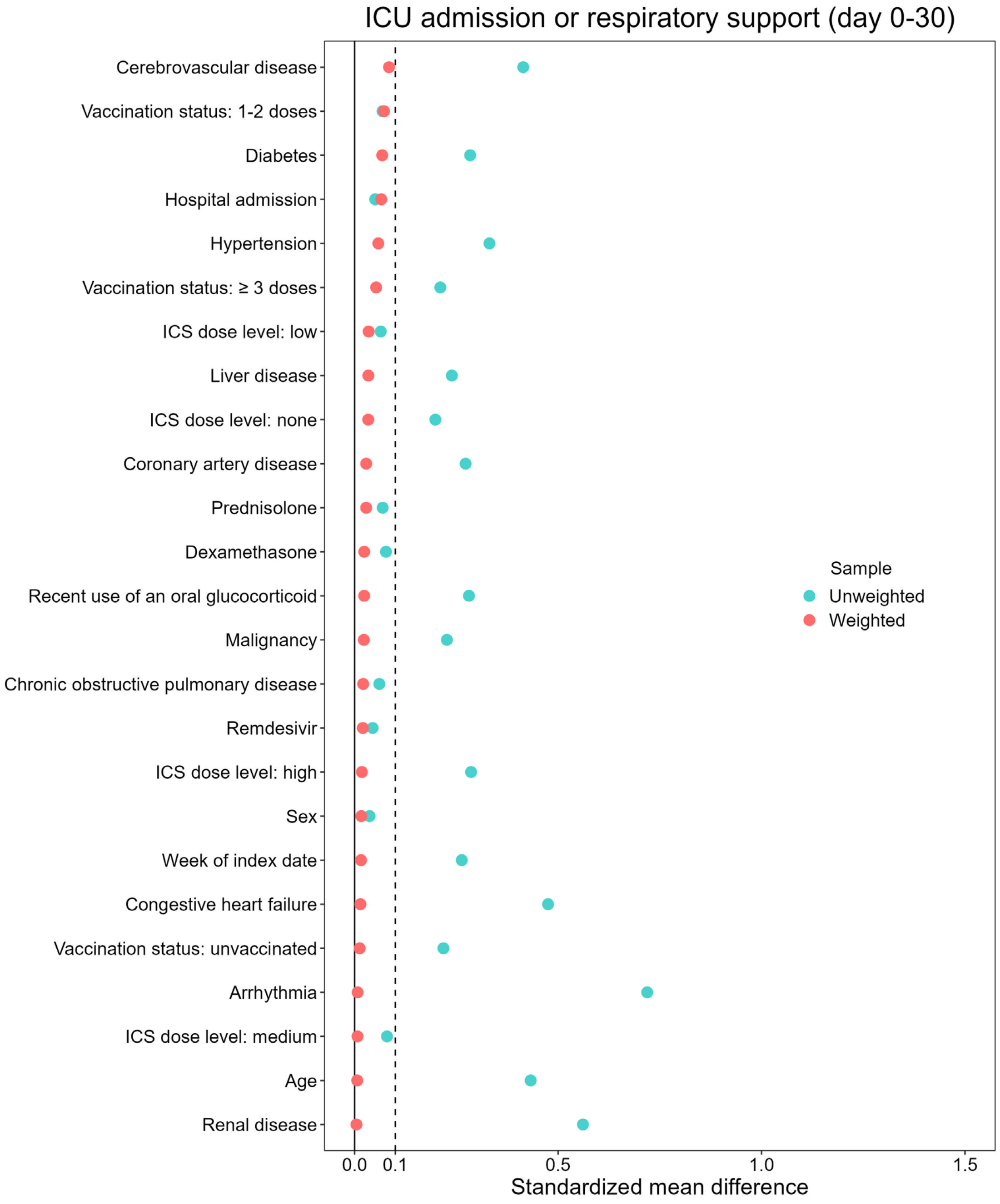

Supplement Figure 4A. Standardized mean difference for each covariate in the comparison between the nirmatrelvir/ritonavir group and control group for the outcome of ICU admission or respiratory support (day 31–365) before and after weighting

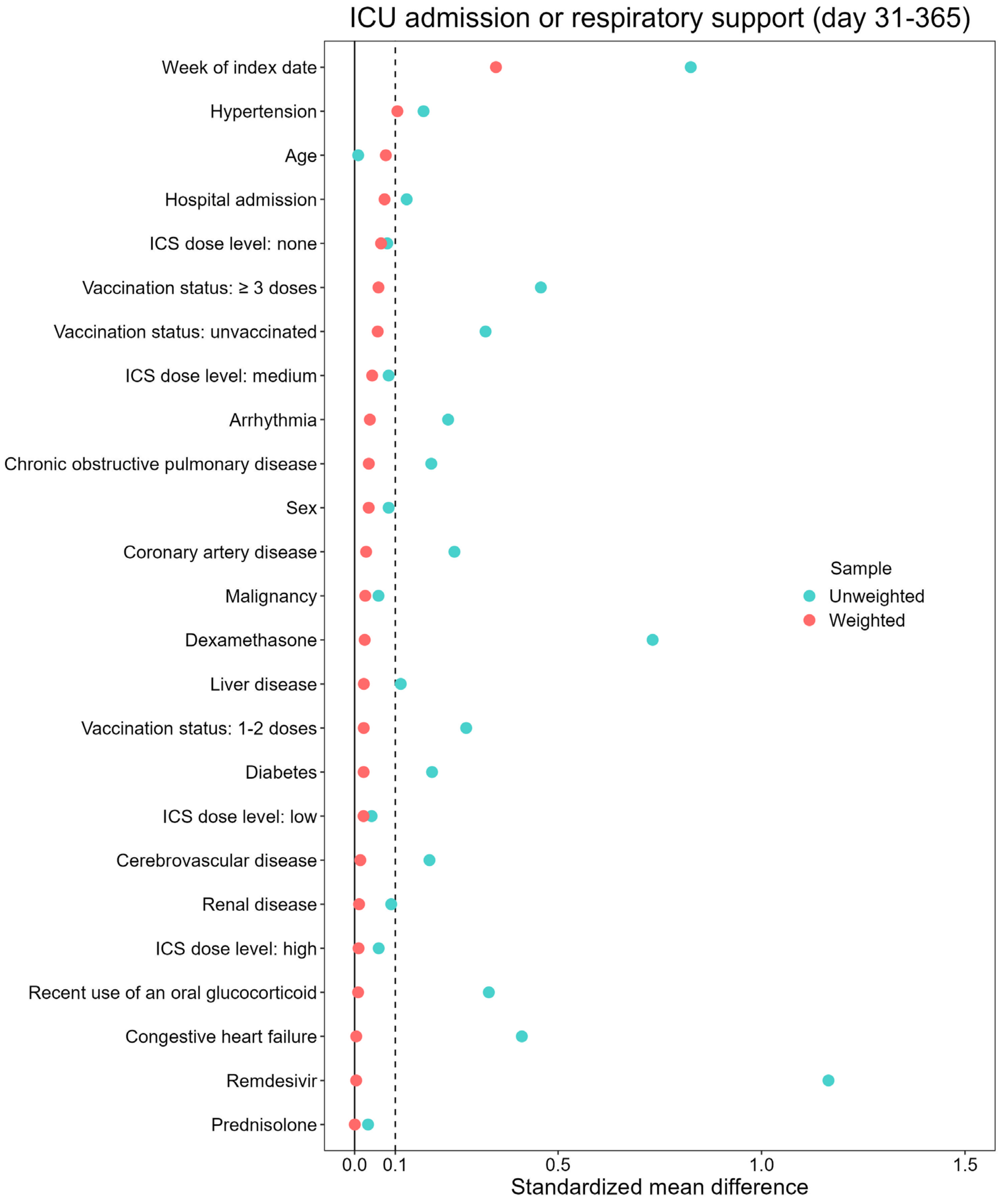

Supplement Figure 4B. Standardized mean difference for each covariate in the comparison between the molnupiravir group and control group for the outcome of ICU admission or respiratory support (day 31–365) before and after weighting

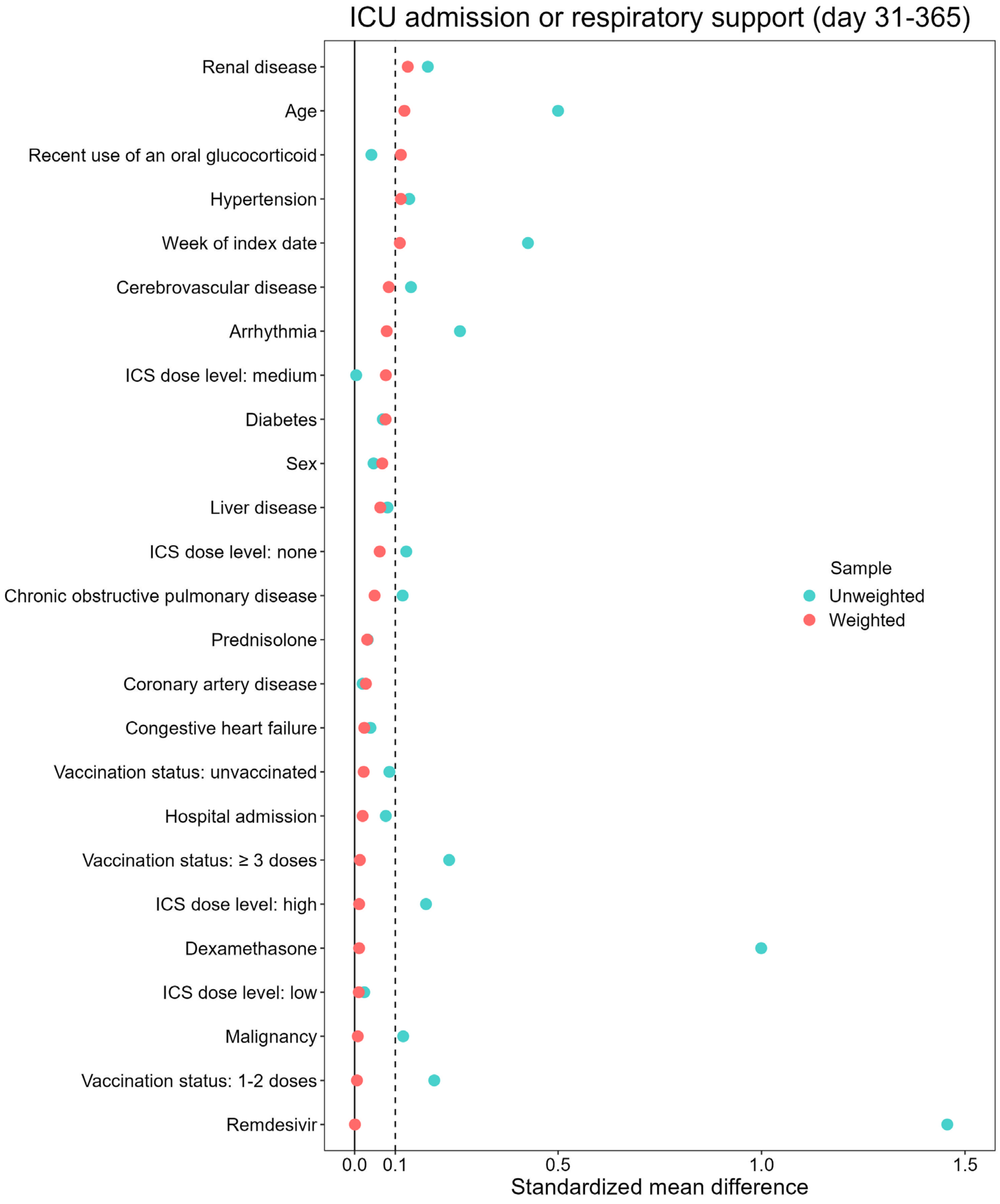

Supplement Figure 4C. Standardized mean difference for each covariate in the comparison between the nirmatrelvir/ritonavir group and molnupiravir group for the outcome of ICU admission or respiratory support (day 31–365) before and after weighting

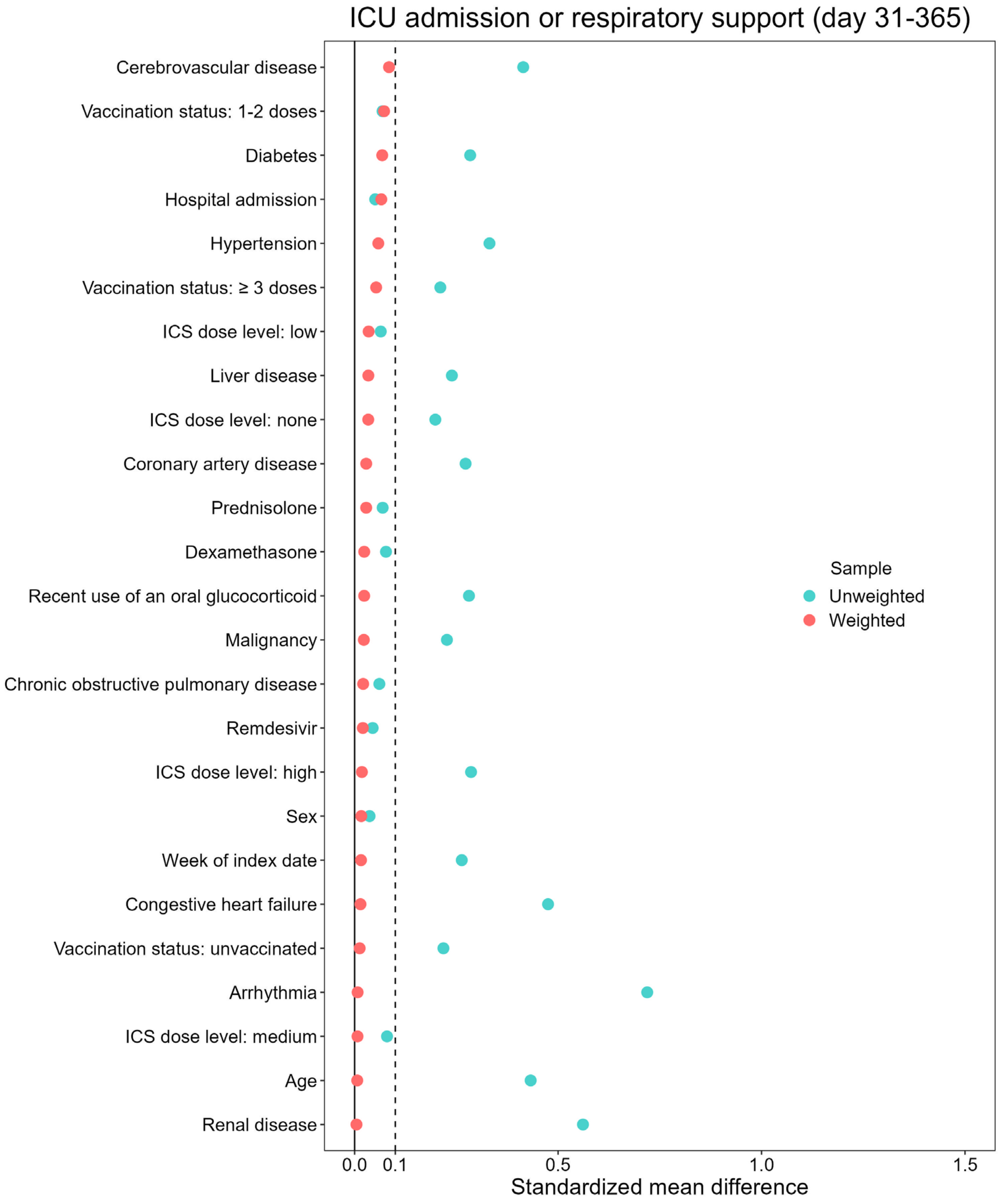

Supplement Figure 5A. Standardized mean difference for each covariate in the comparison between the nirmatrelvir/ritonavir group and control group for the outcome of all-cause hospitalization before and after weighting

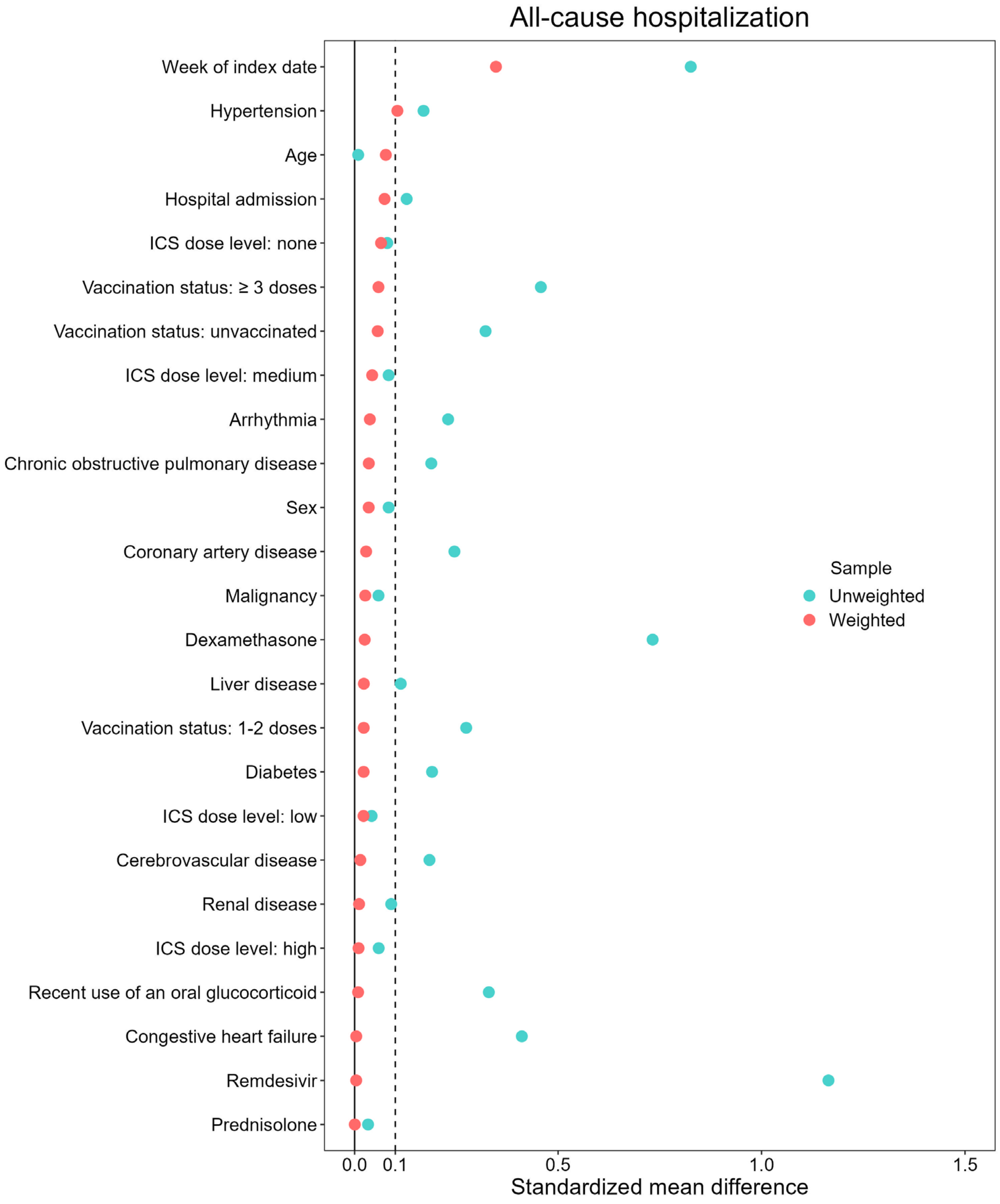

Supplement Figure 5B. Standardized mean difference for each covariate in the comparison between the molnupiravir group and control group for the outcome of all-cause hospitalization before and after weighting

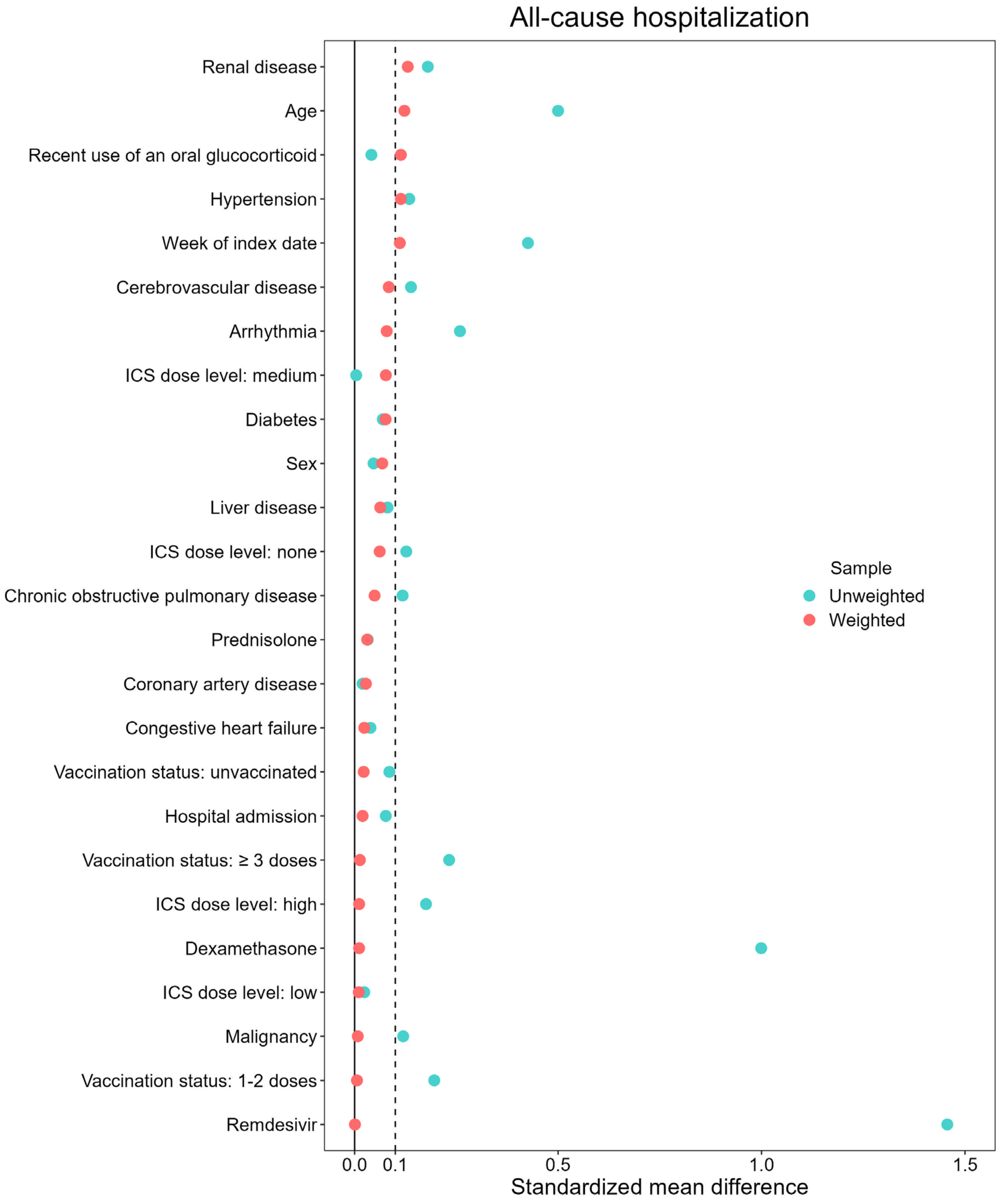

Supplement Figure 5C. Standardized mean difference for each covariate in the comparison between the nirmatrelvir/ritonavir group and molnupiravir group for the outcome of all-cause hospitalization before and after weighting

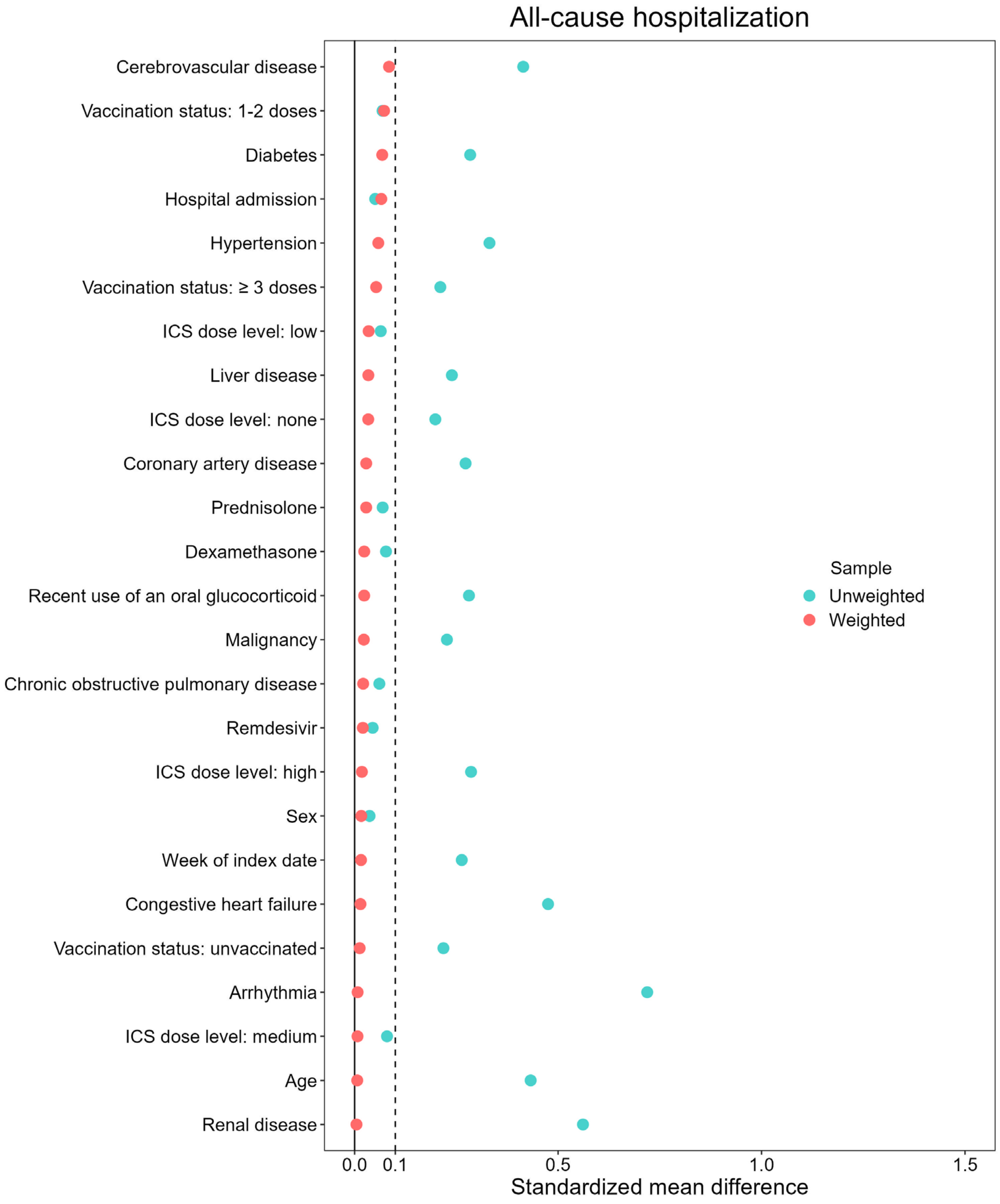

Supplement Figure 6A. Standardized mean difference for each covariate in the comparison between the nirmatrelvir/ritonavir group and control group for the outcome of chronic pulmonary disease excluding asthma before and after weighting

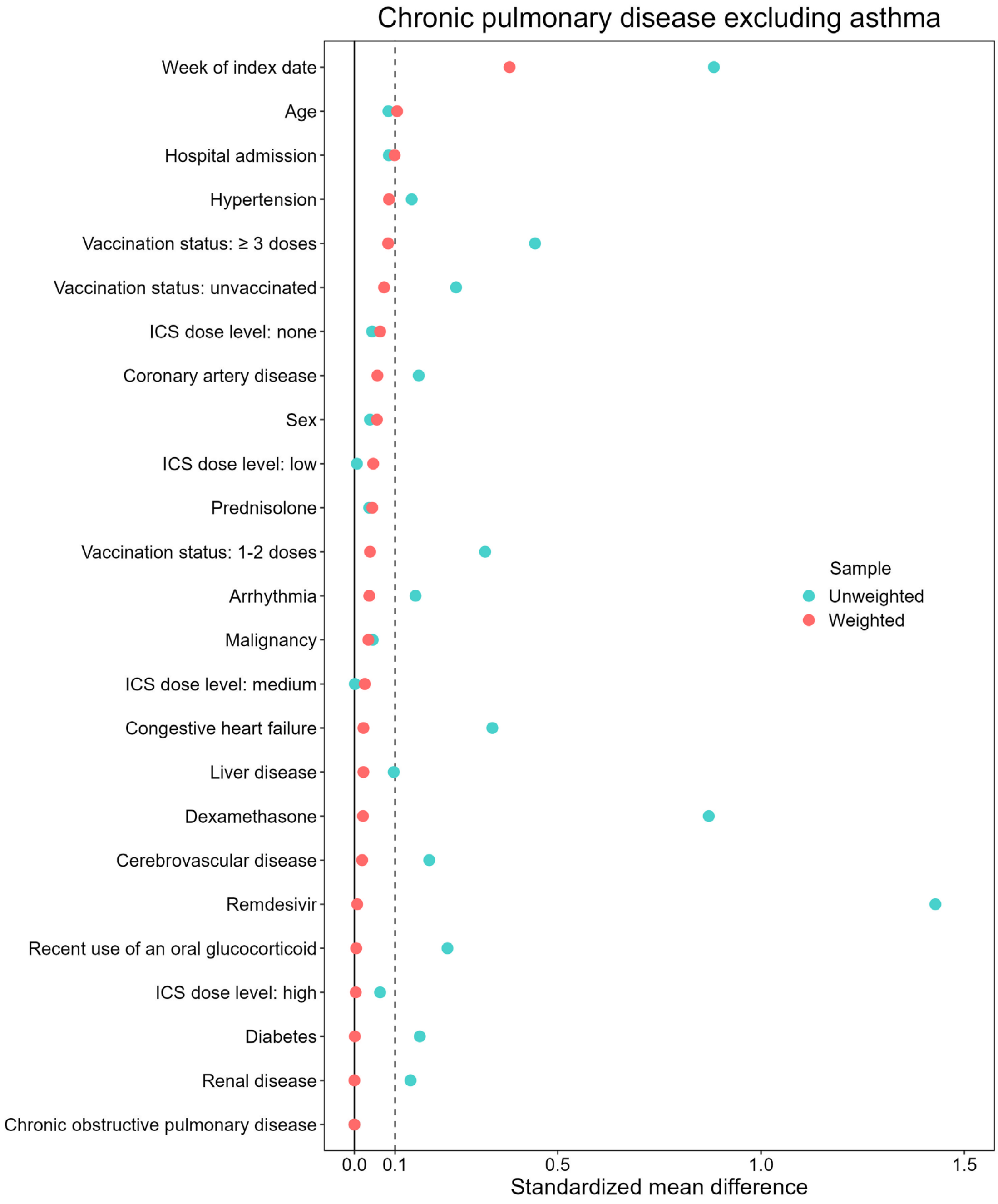

Supplement Figure 6B. Standardized mean difference for each covariate in the comparison between the molnupiravir group and control group for the outcome of chronic pulmonary disease excluding asthma before and after weighting

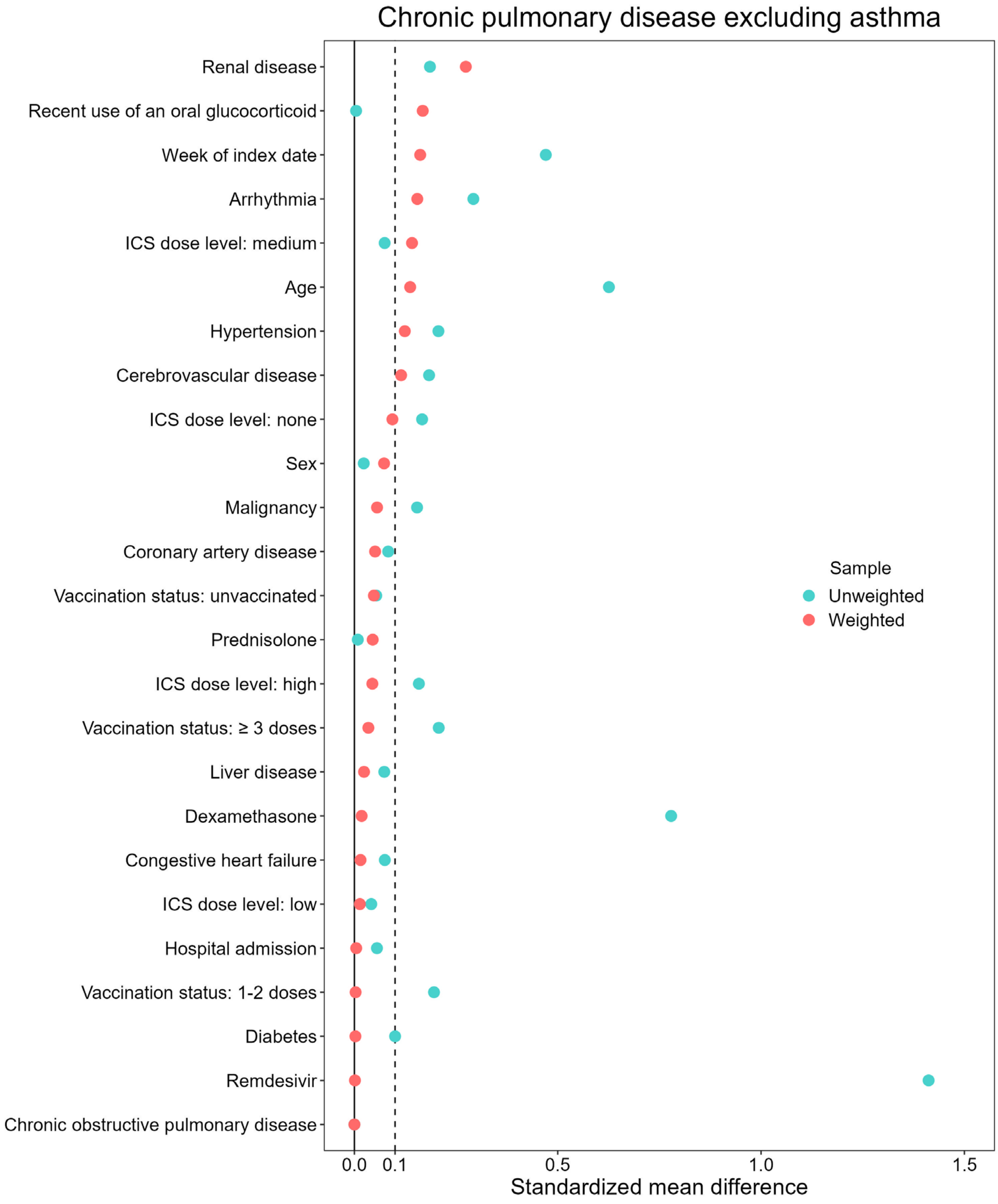

Supplement Figure 6C. Standardized mean difference for each covariate in the comparison between the nirmatrelvir/ritonavir group and molnupiravir group for the outcome of chronic pulmonary disease excluding asthma before and after weighting

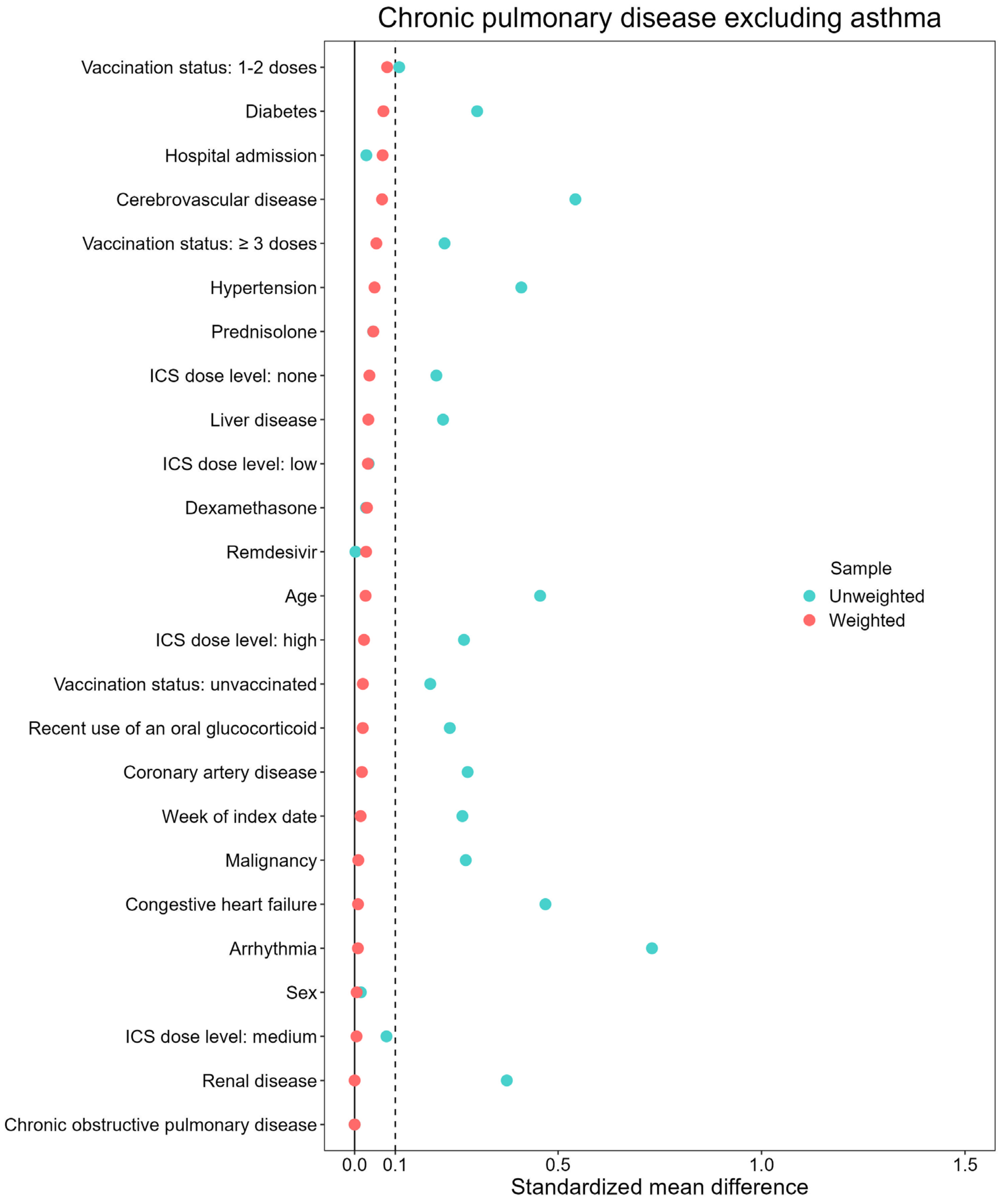

Supplement Figure 7A. Standardized mean difference for each covariate in the comparison between the nirmatrelvir/ritonavir group and control group for the outcome of acute respiratory distress syndrome before and after weighting

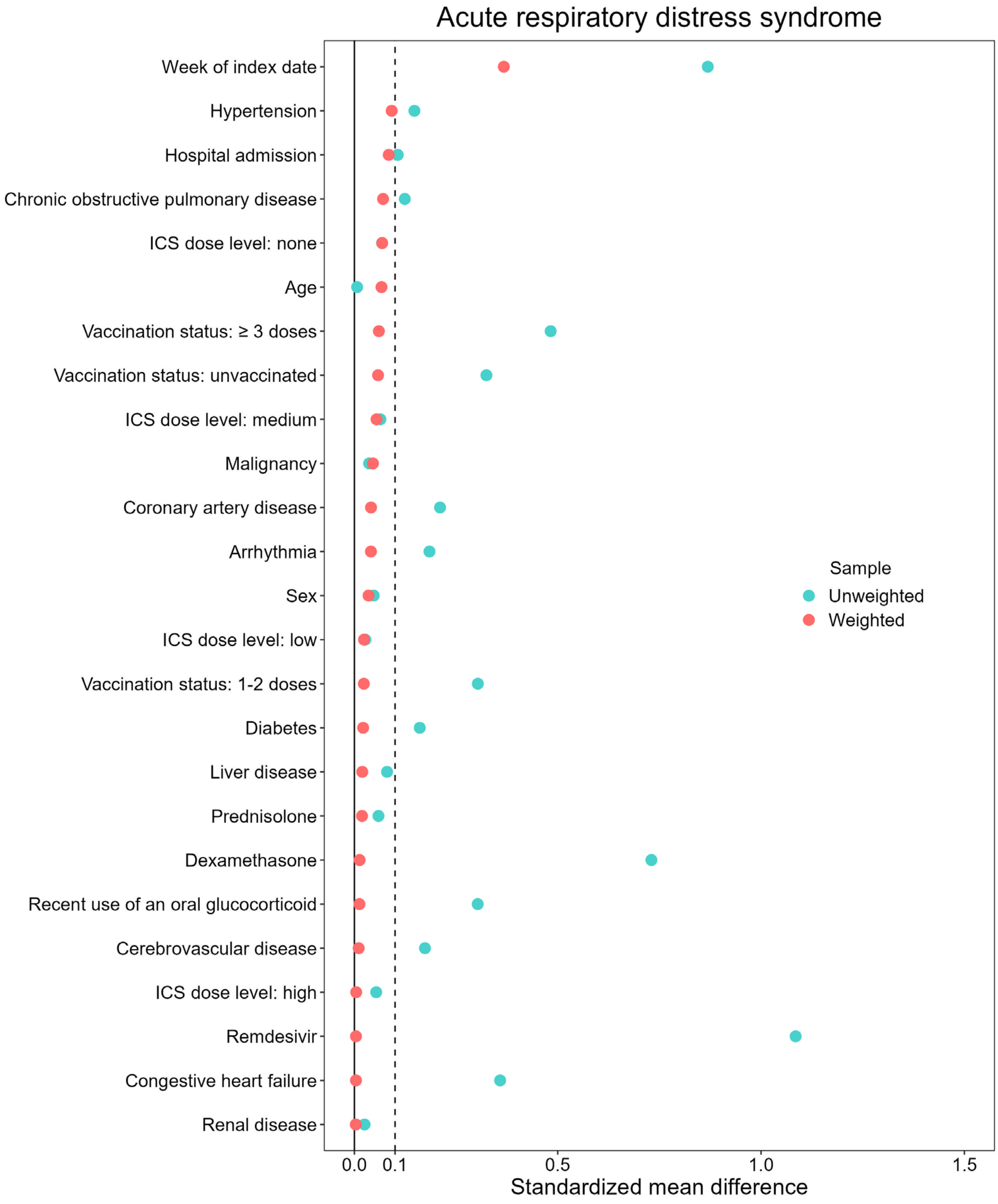

Supplement Figure 7B. Standardized mean difference for each covariate in the comparison between the molnupiravir group and control group for the outcome of acute respiratory distress syndrome before and after weighting

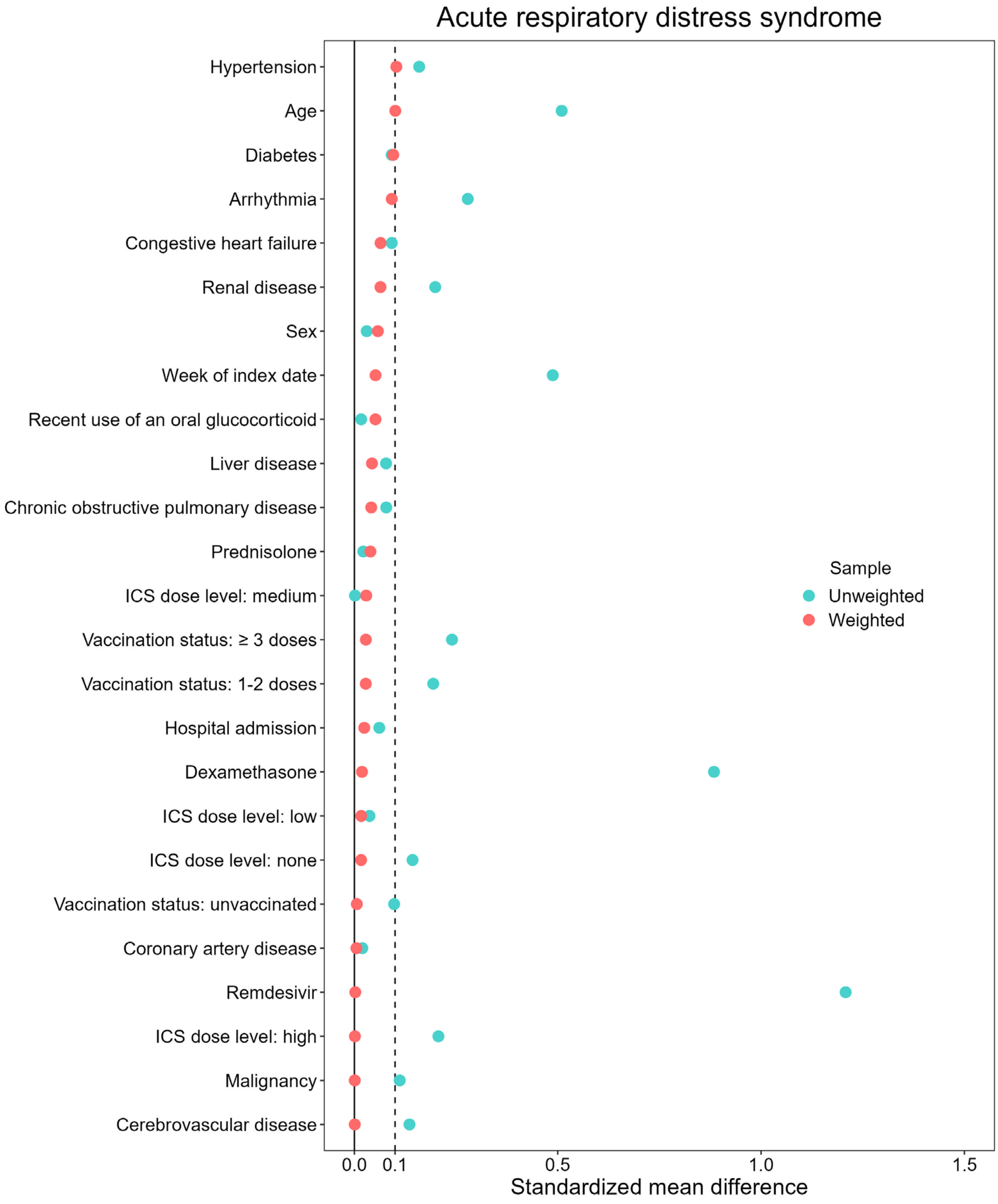

Supplement Figure 7C. Standardized mean difference for each covariate in the comparison between the nirmatrelvir/ritonavir group and molnupiravir group for the outcome of acute respiratory distress syndrome before and after weighting

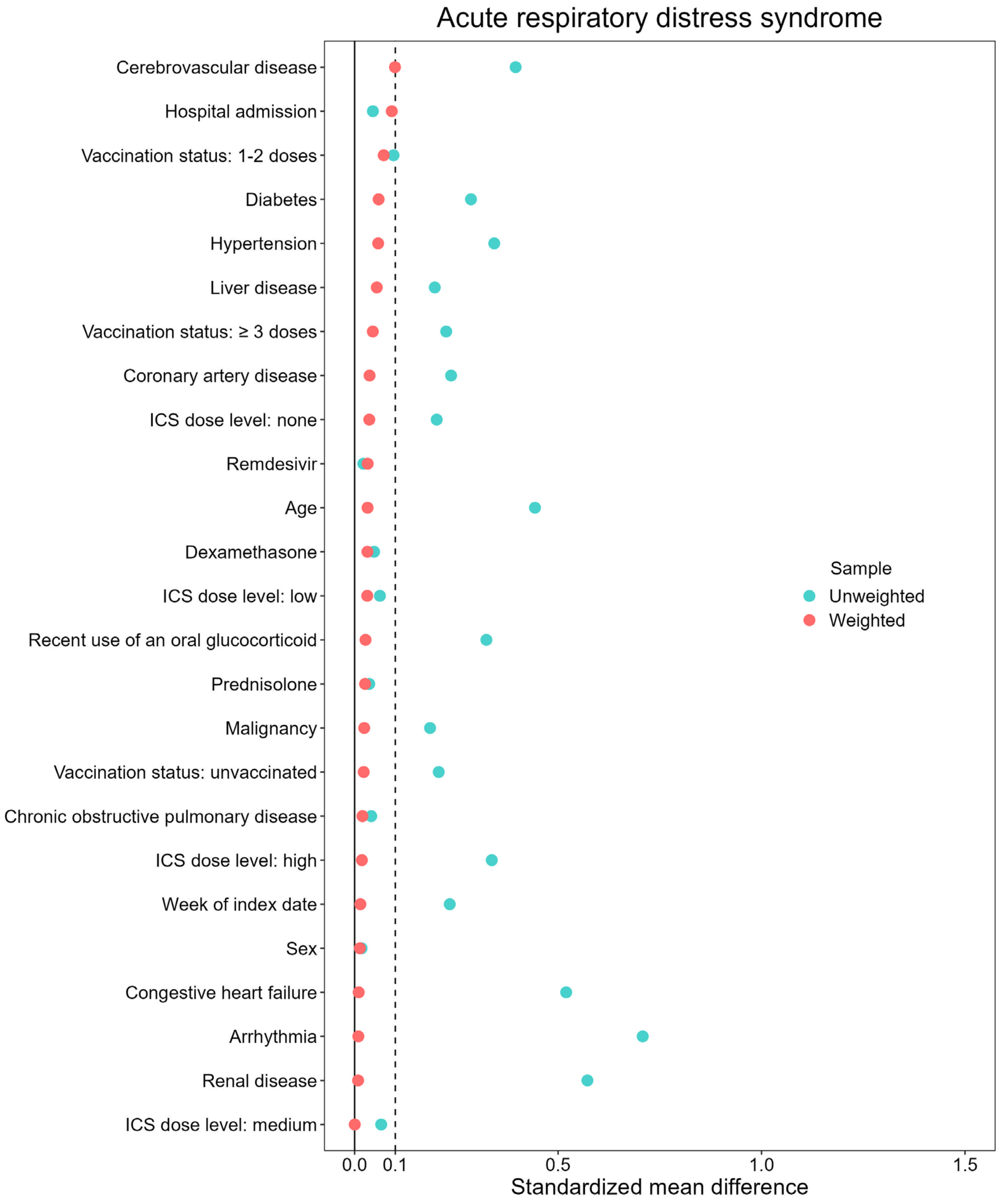

Supplement Figure 8A. Standardized mean difference for each covariate in the comparison between the nirmatrelvir/ritonavir group and control group for the outcome of myocardial infarction before and after weighting

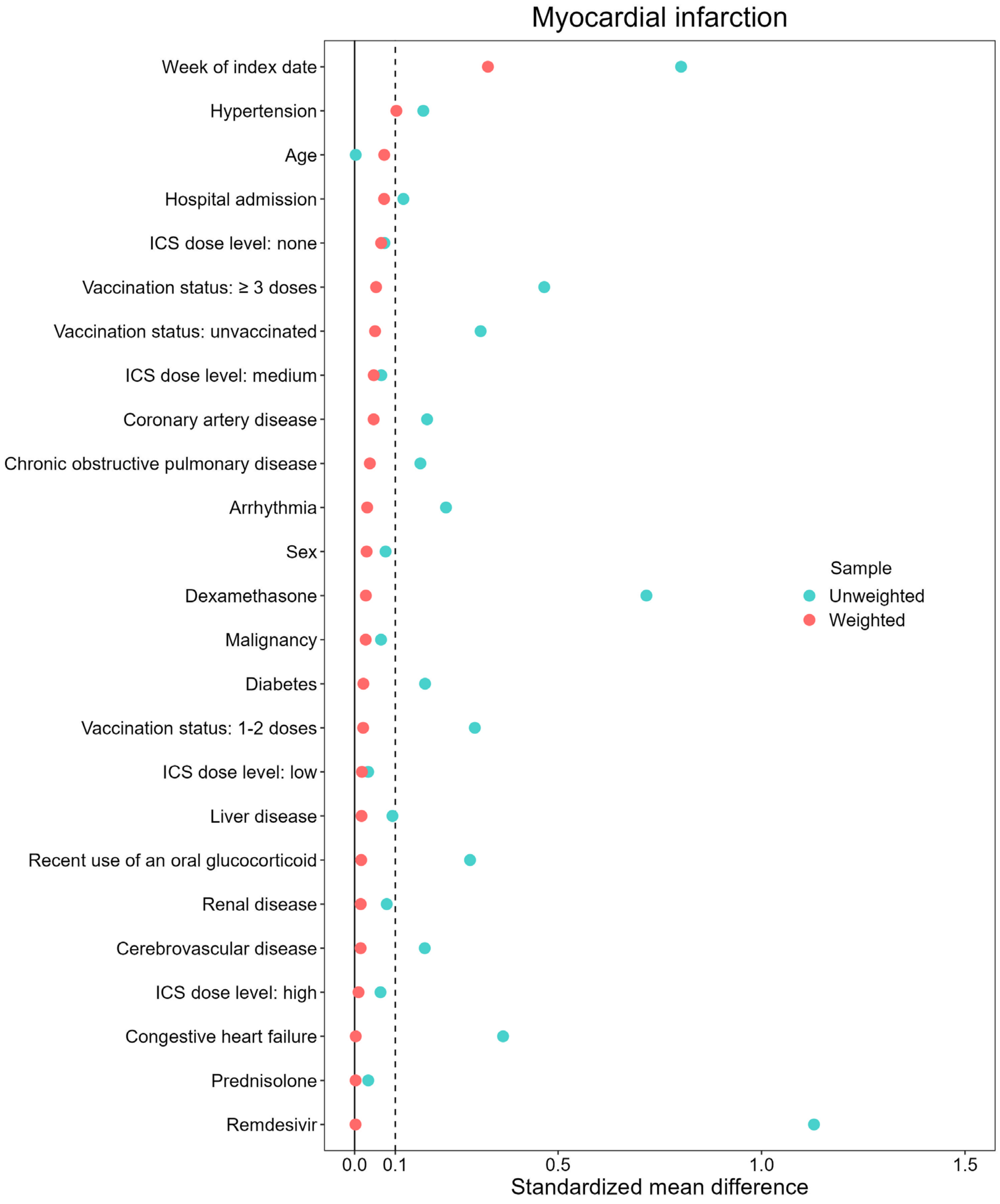

Supplement Figure 8B. Standardized mean difference for each covariate in the comparison between the molnupiravir group and control group for the outcome of myocardial infarction before and after weighting

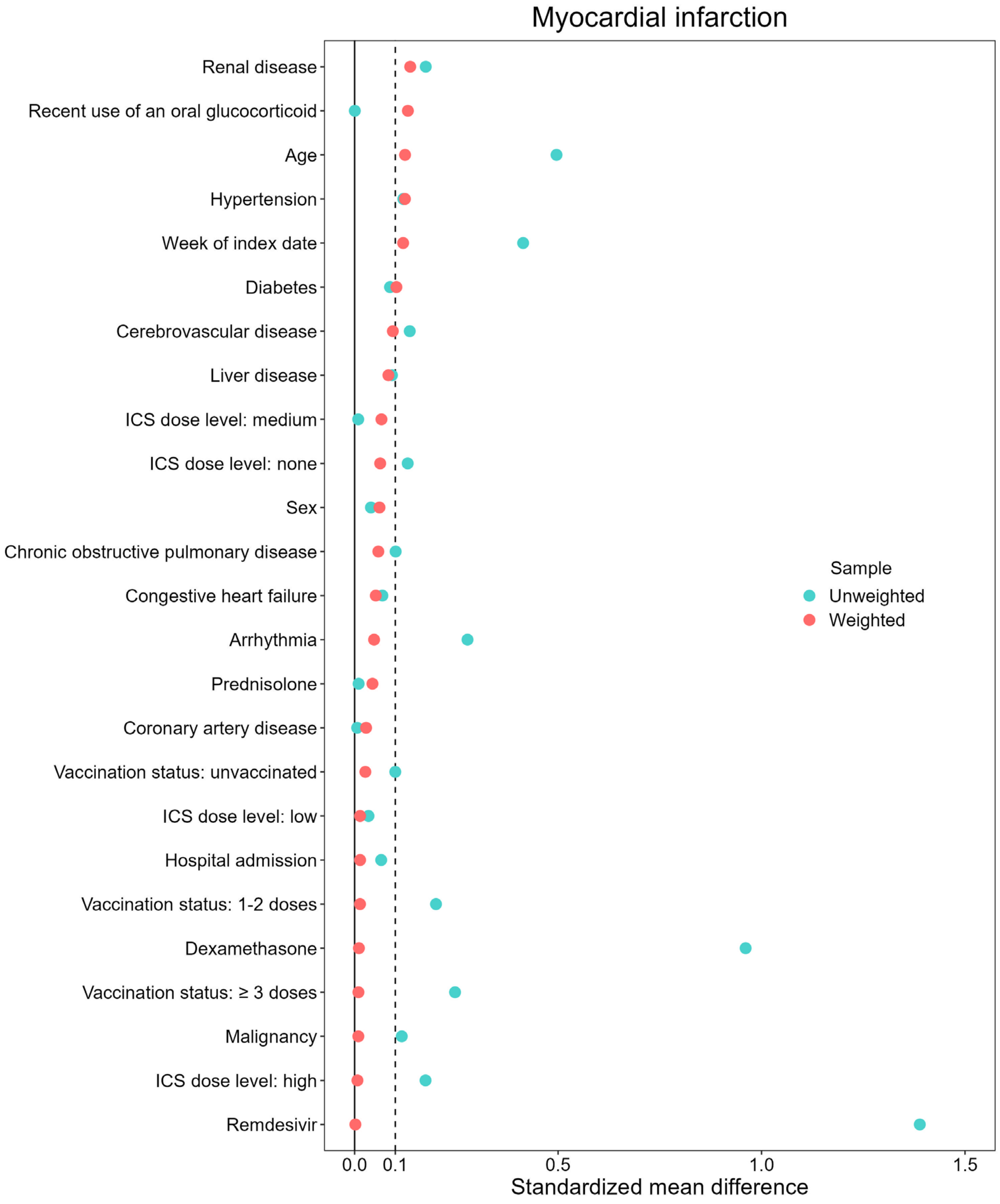

Supplement Figure 8C. Standardized mean difference for each covariate in the comparison between the nirmatrelvir/ritonavir group and molnupiravir group for the outcome of myocardial infarction before and after weighting

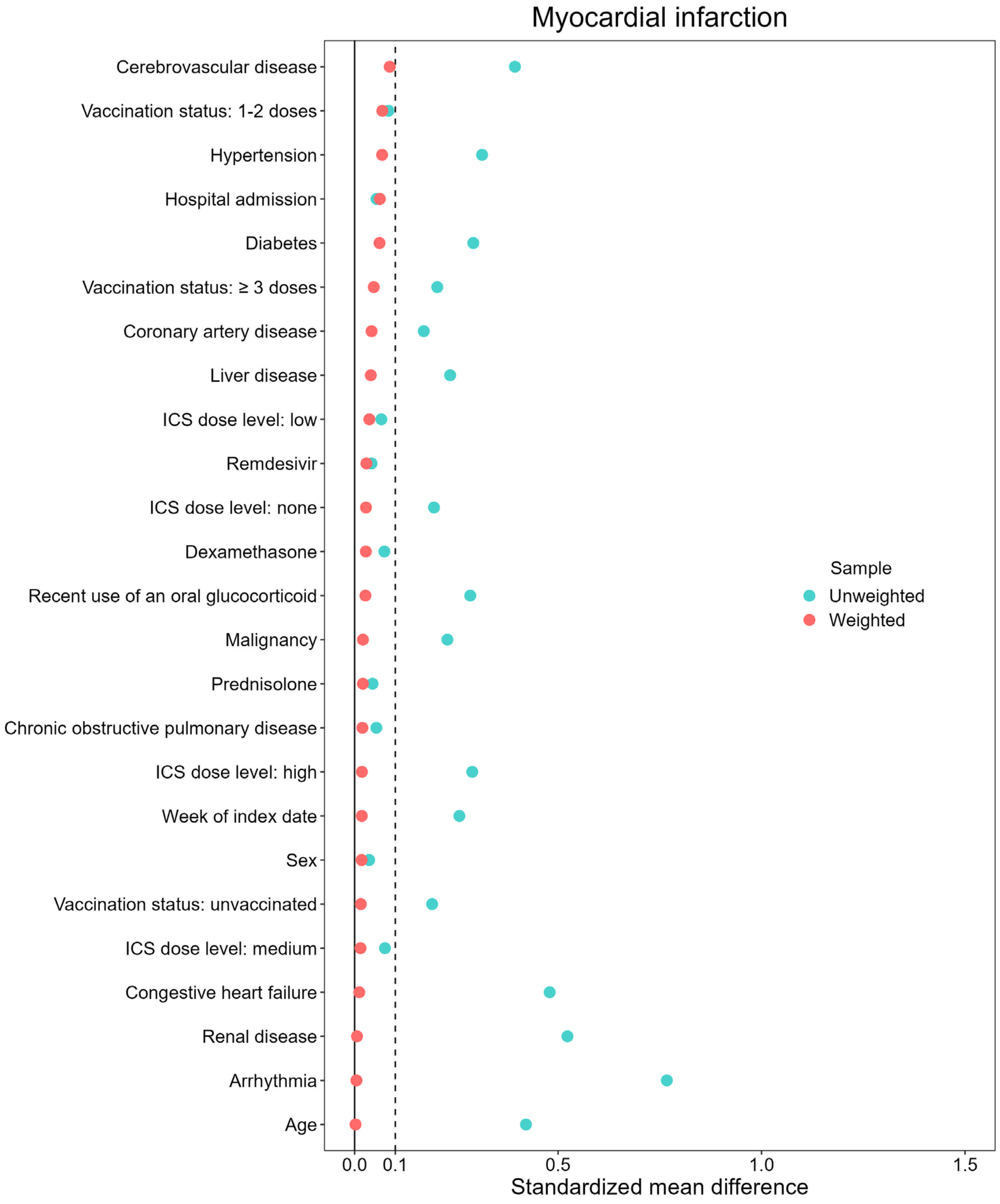

Supplement Figure 9A. Standardized mean difference for each covariate in the comparison between the nirmatrelvir/ritonavir group and control group for the outcome of stroke before and after weighting

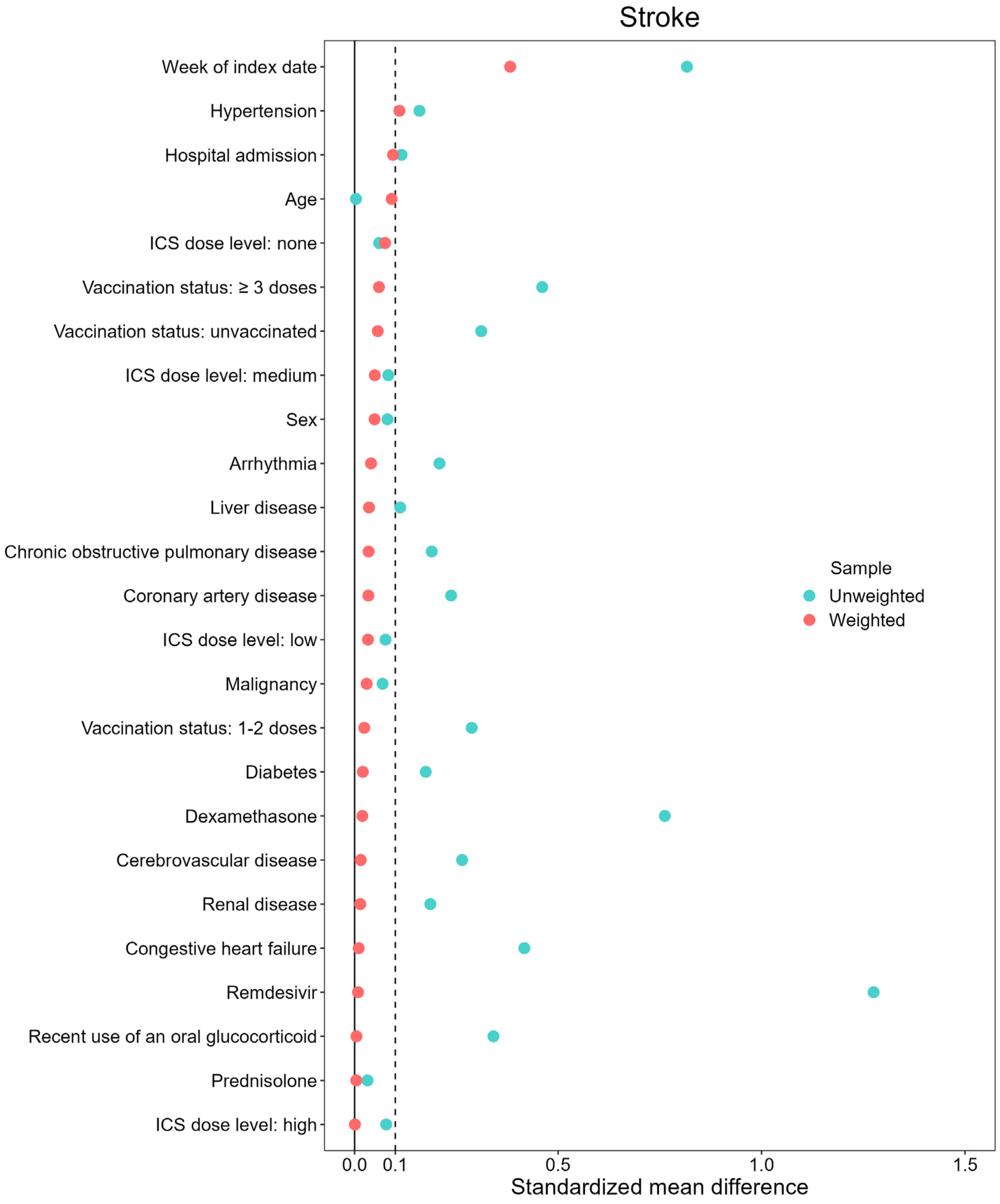

Supplement Figure 9B. Standardized mean difference for each covariate in the comparison between the molnupiravir group and control group for the outcome of stroke before and after weighting

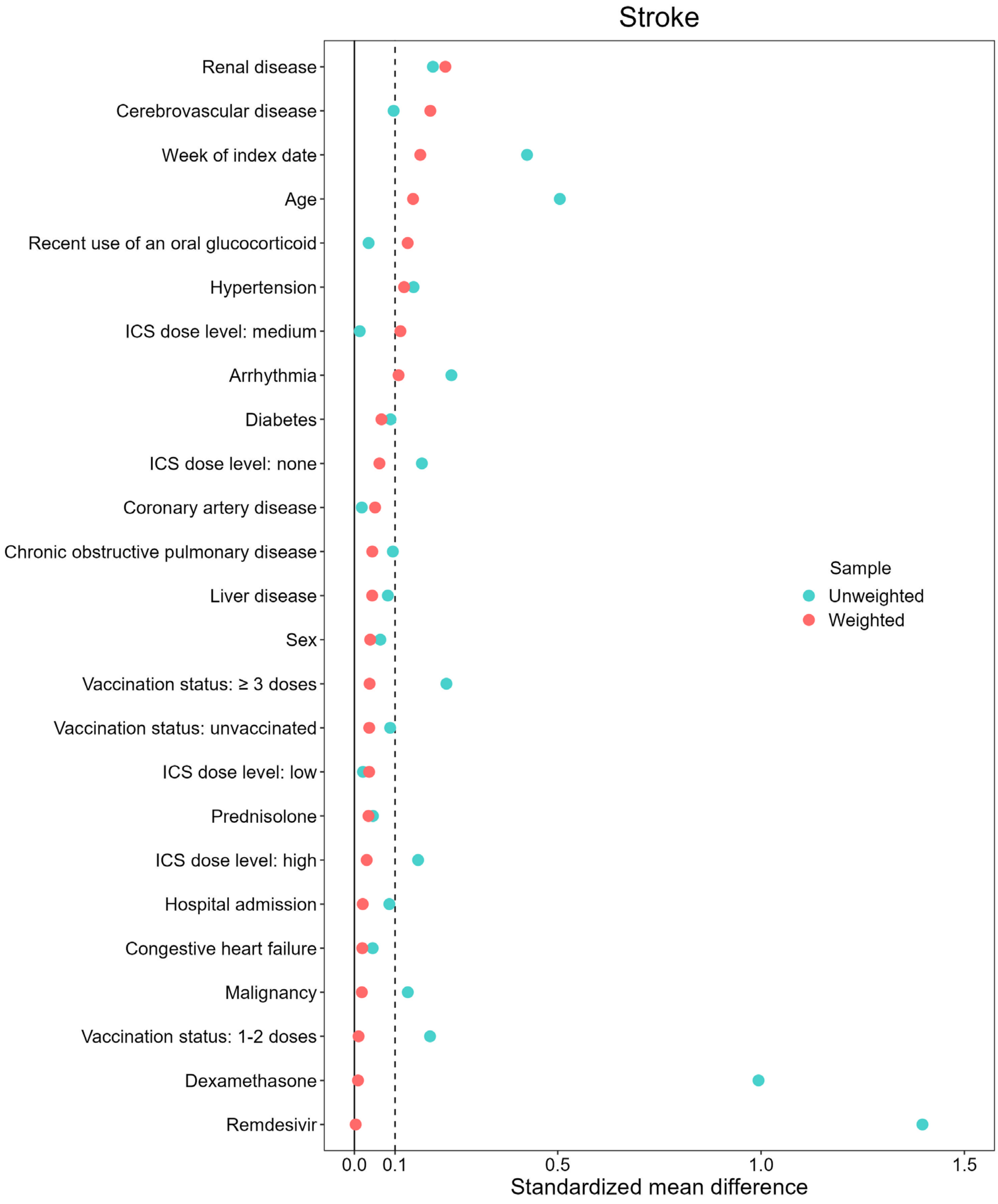

Supplement Figure 9C. Standardized mean difference for each covariate in the comparison between the nirmatrelvir/ritonavir group and molnupiravir group for the outcome of stroke before and after weighting

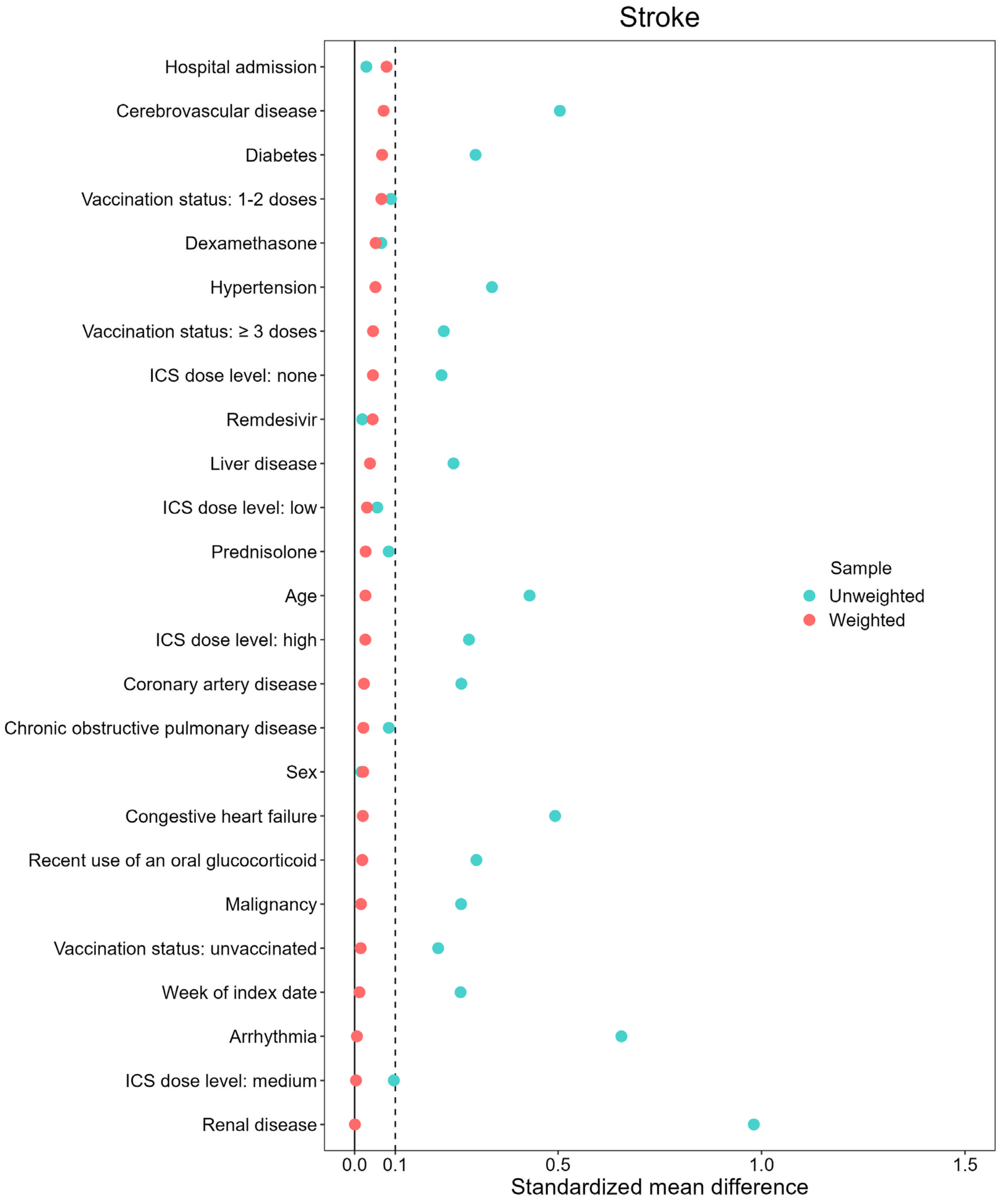

Supplement Figure 10A. Standardized mean difference for each covariate in the comparison between the nirmatrelvir/ritonavir group and control group for the outcome of asthma exacerbation before and after weighting

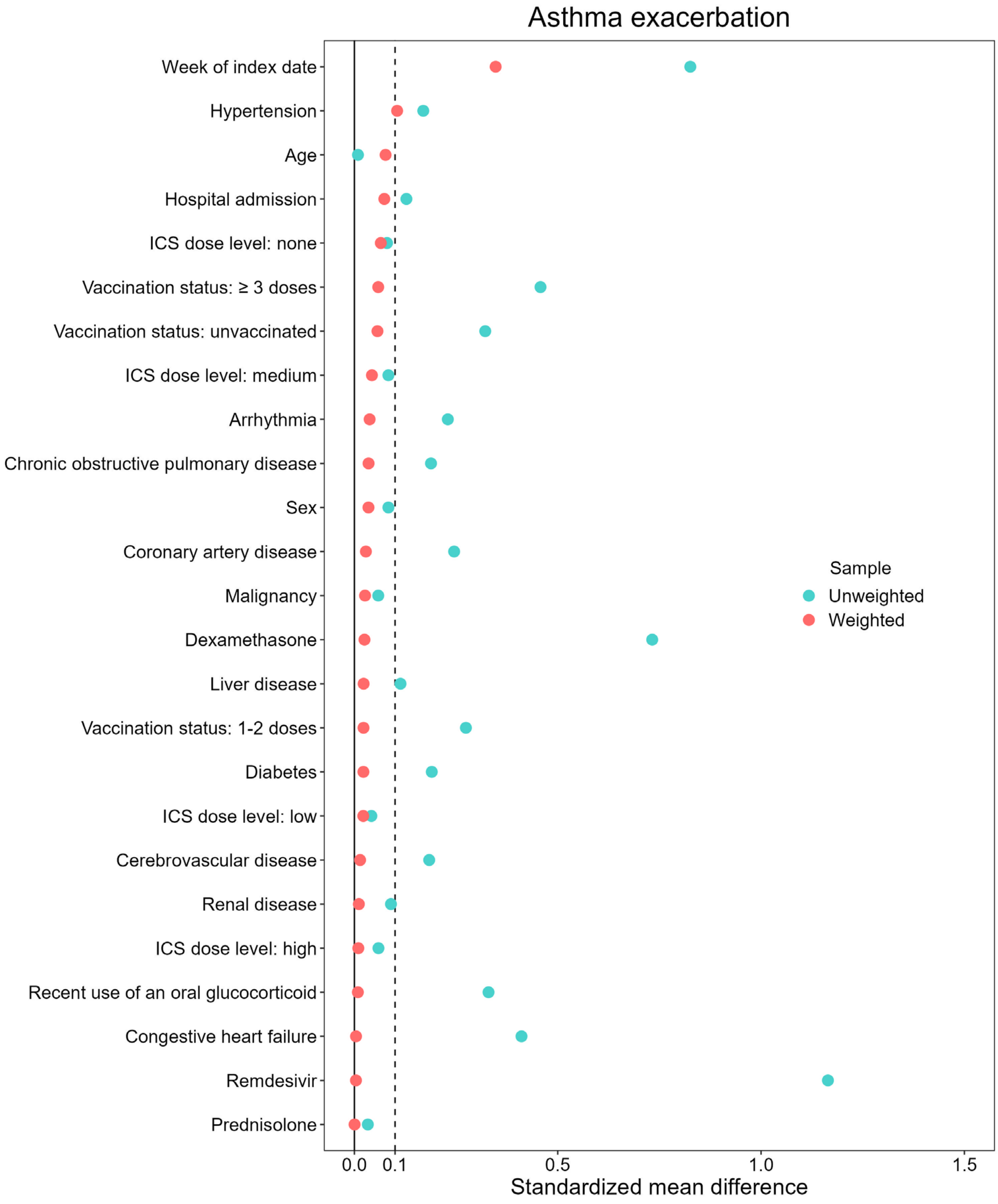

Supplement Figure 10B. Standardized mean difference for each covariate in the comparison between the molnupiravir group and control group for the outcome of asthma exacerbation before and after weighting

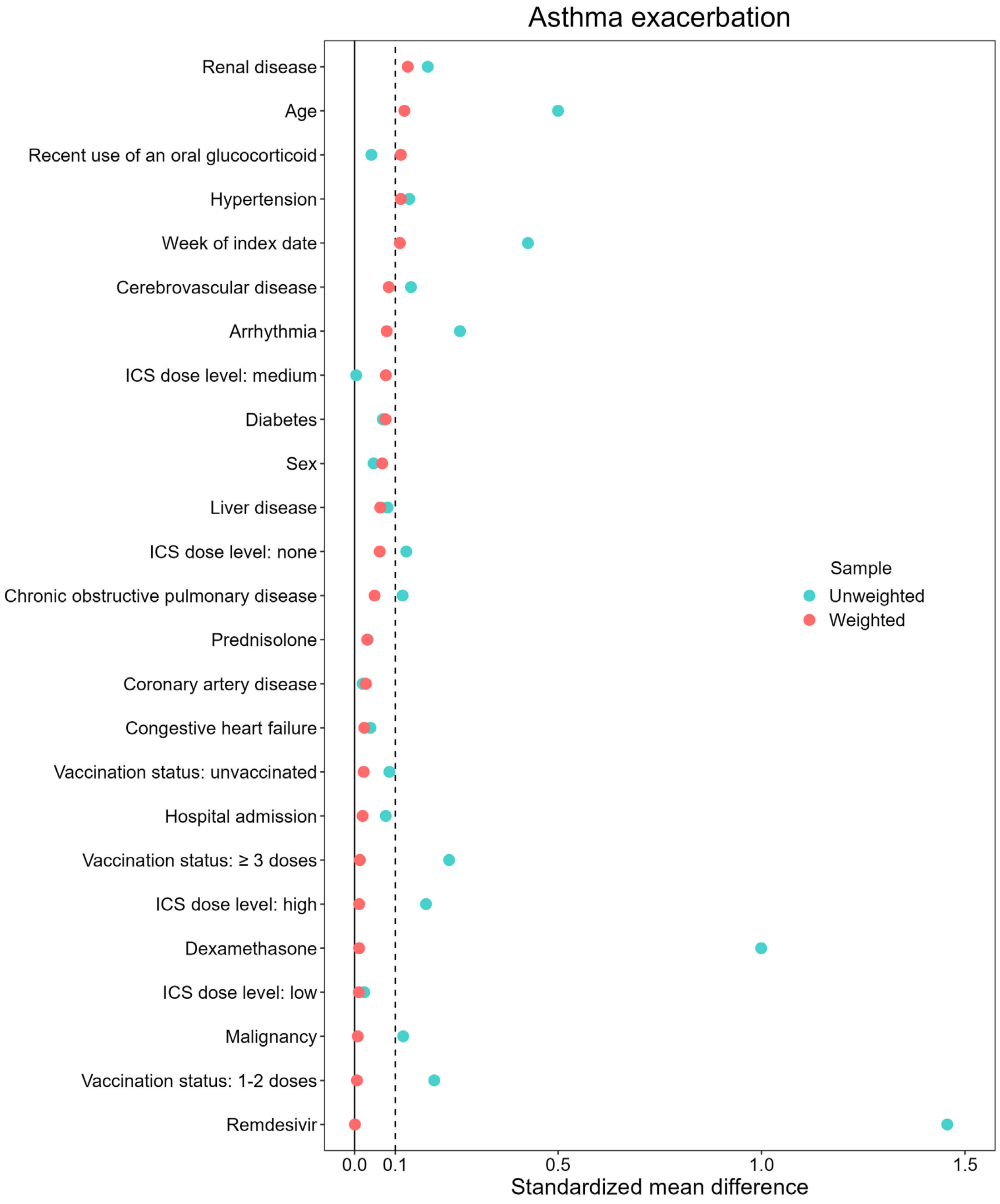

Supplement Figure 10C. Standardized mean difference for each covariate in the comparison between the nirmatrelvir/ritonavir group and molnupiravir group for the outcome of asthma exacerbation before and after weighting

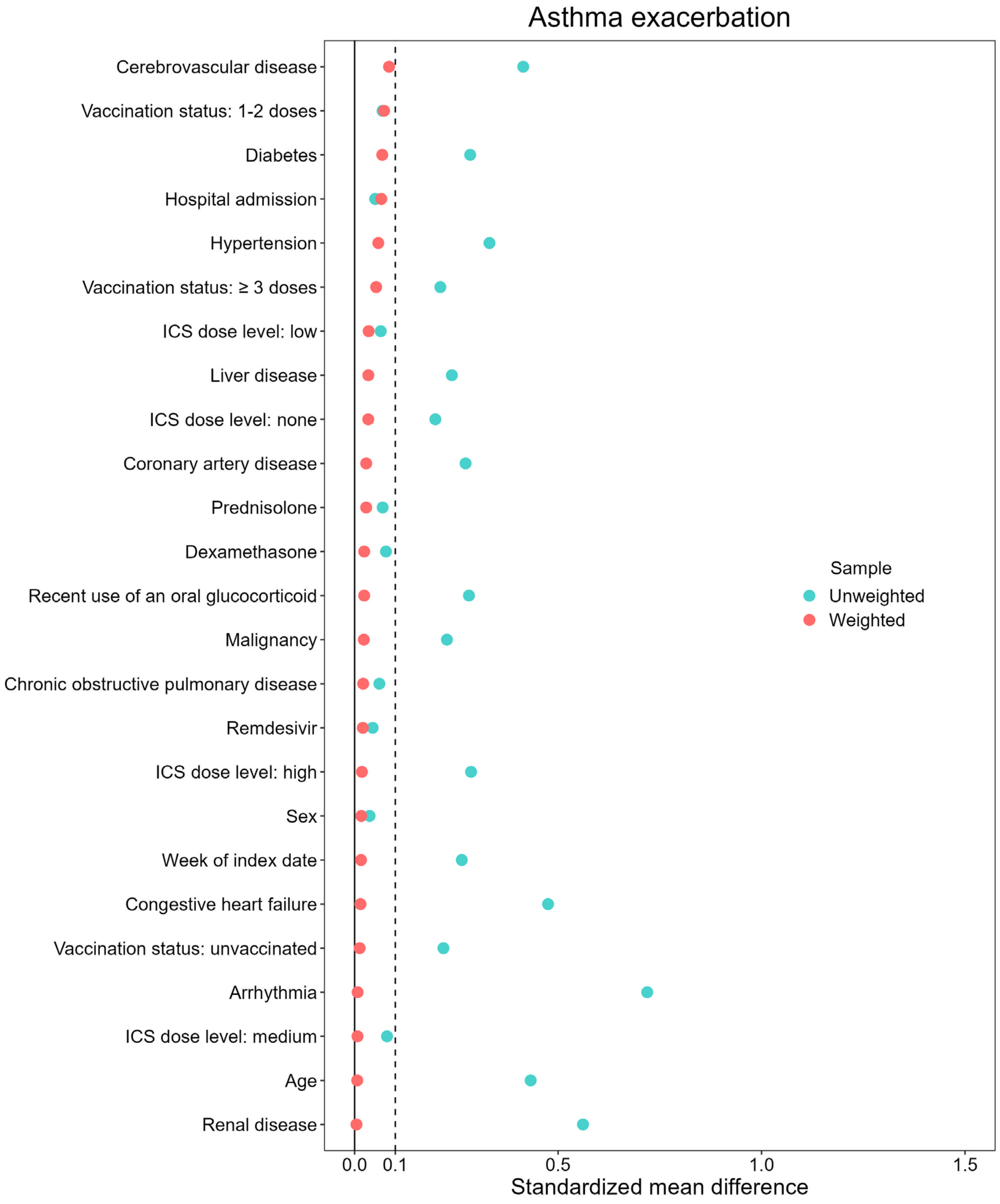

Supplement Figure 11A. Scaled Schoenfeld residual plots for the comparison between the nirmatrelvir/ritonavir group and control group

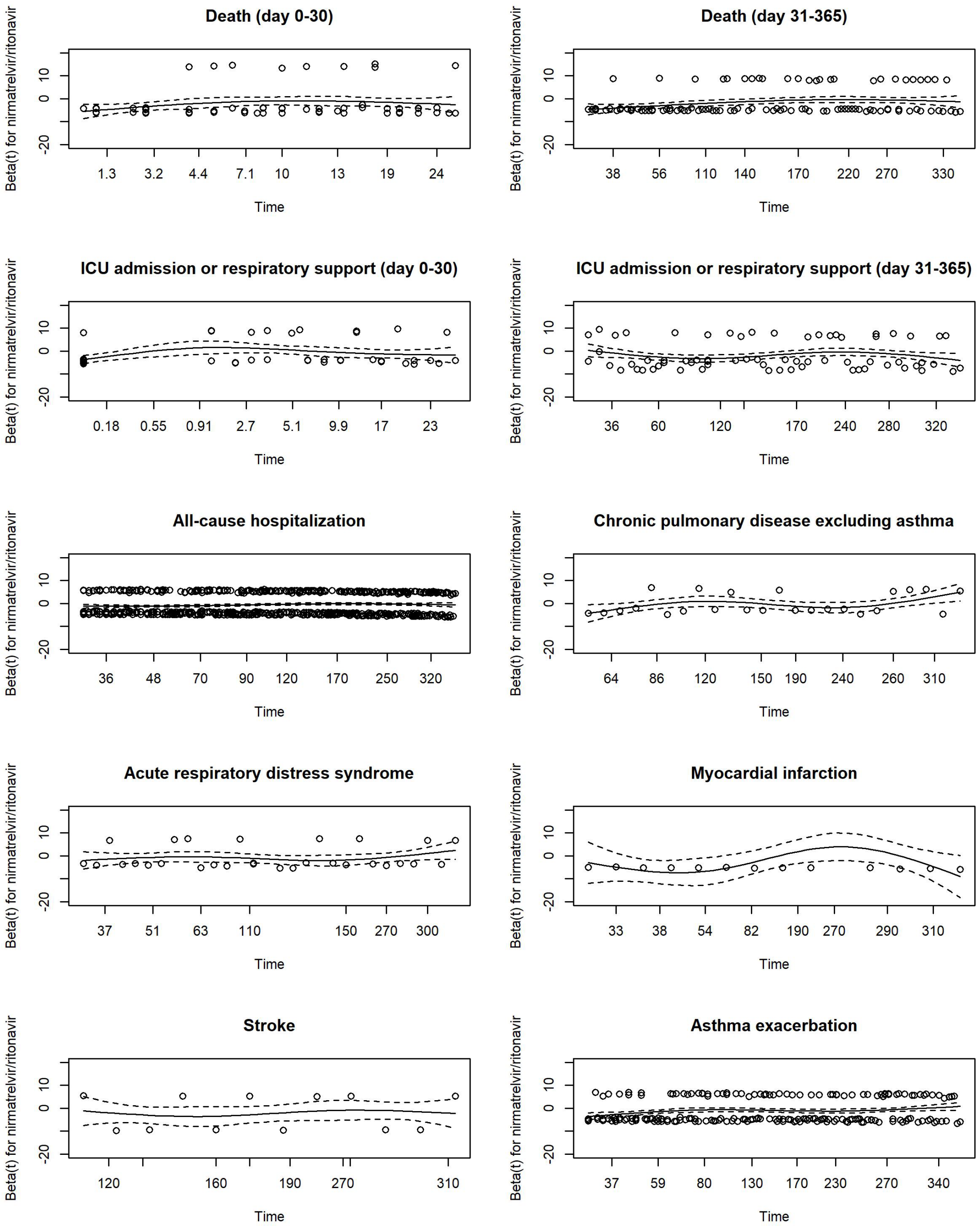

Supplement Figure 11B. Scaled Schoenfeld residual plots for the comparison between the molnupiravir group and control group

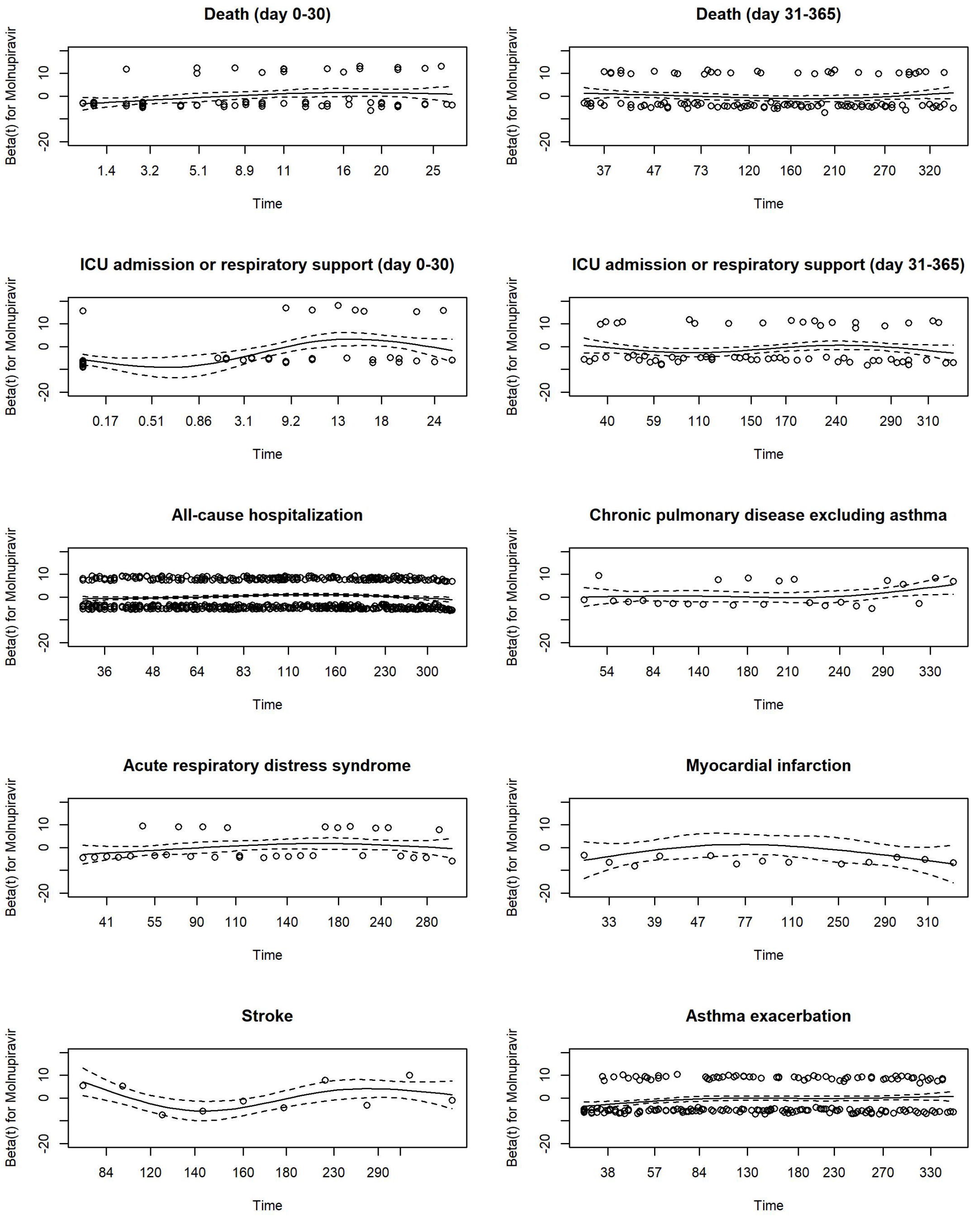

Supplement Figure 11C. Scaled Schoenfeld residual plots for the comparison between the nirmatrelvir/ritonavir group and molnupiravir group

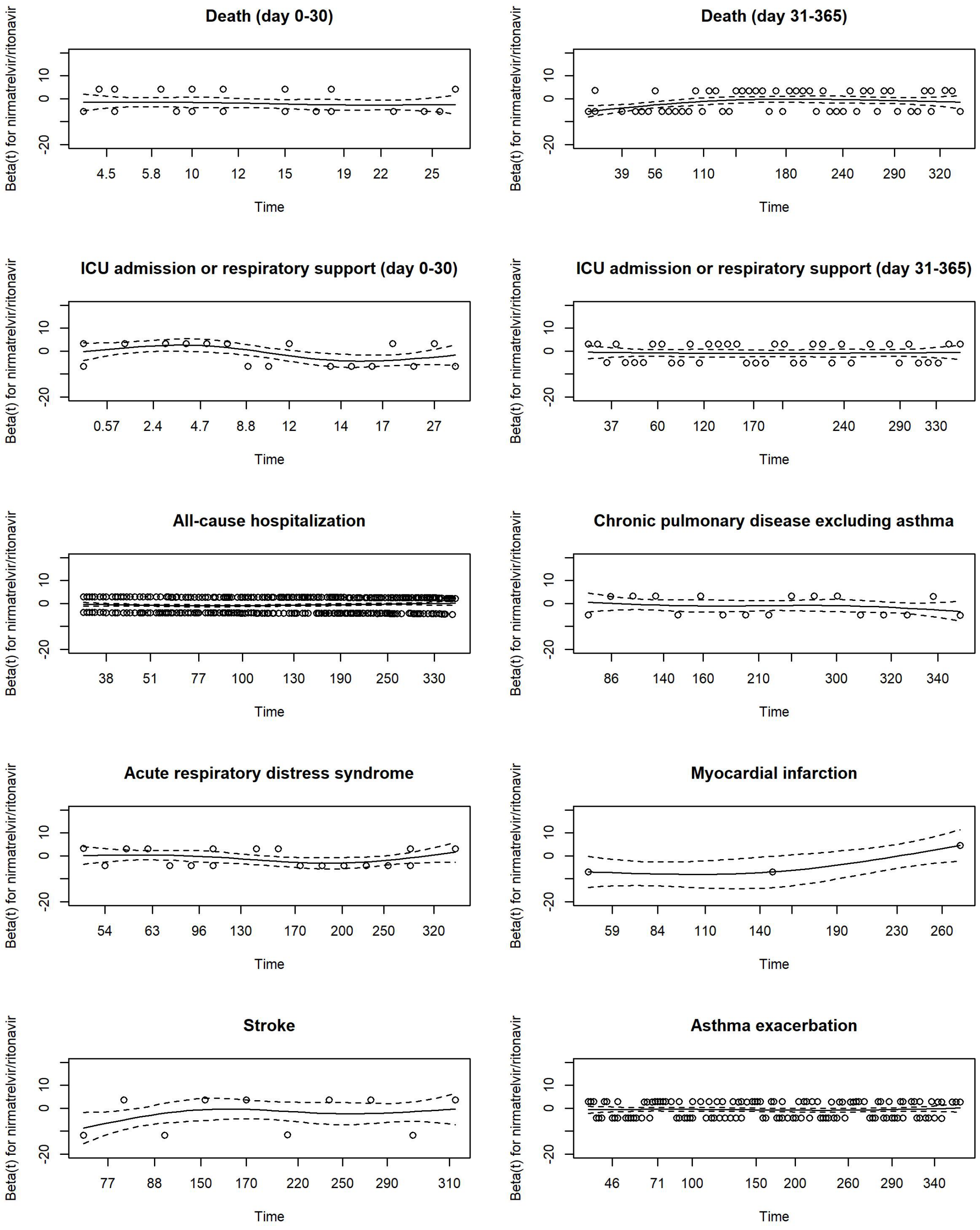

Supplement Figure 12A. Subgroup analysis: vaccination status in the comparison between the nirmatrelvir/ritonavir group and control group

Outcome	Control (Event/No. at risk [%])	N/R (Event/No. at risk [%])	HR (95% CI)	P value
Death				
Day 0-30				
< 3 doses	34/387 (8.8)	4/191 (2.1)	0.36 (0.12, 1.09)	0.071
≥ 3 doses	19/359 (5.3)	5/430 (1.2)	0.28 (0.09, 0.90)	0.032
Day 31-365				
< 3 doses	56/387 (14.5)	15/191 (7.9)	0.62 (0.31, 1.25)	0.18
≥ 3 doses	23/359 (6.4)	10/430 (2.3)	0.54 (0.22, 1.34)	0.18
ICU admission or respiratory support				
Day 0-30				
< 3 doses	19/387 (4.9)	5/191 (2.6)	0.60 (0.17, 2.12)	0.43
≥ 3 doses	17/359 (4.7)	6/430 (1.4)	0.35 (0.11, 1.11)	0.075
Day 31-365				
< 3 doses	26/387 (6.7)	12/191 (6.3)	1.24 (0.45, 3.41)	0.68
≥ 3 doses	20/359 (5.6)	9/430 (2.1)	0.51 (0.18, 1.42)	0.2
All-cause hospitalization				
< 3 doses	198/387 (51.2)	86/191 (45.0)	0.79 (0.57, 1.10)	0.17
≥ 3 doses	164/359 (45.7)	146/430 (34.0)	0.67 (0.51, 0.89)	0.0049
Asthma exacerbation				
< 3 doses	64/387 (16.5)	22/191 (11.5)	0.68 (0.37, 1.26)	0.22
≥ 3 doses	60/359 (16.7)	48/430 (11.2)	0.88 (0.55, 1.40)	0.59

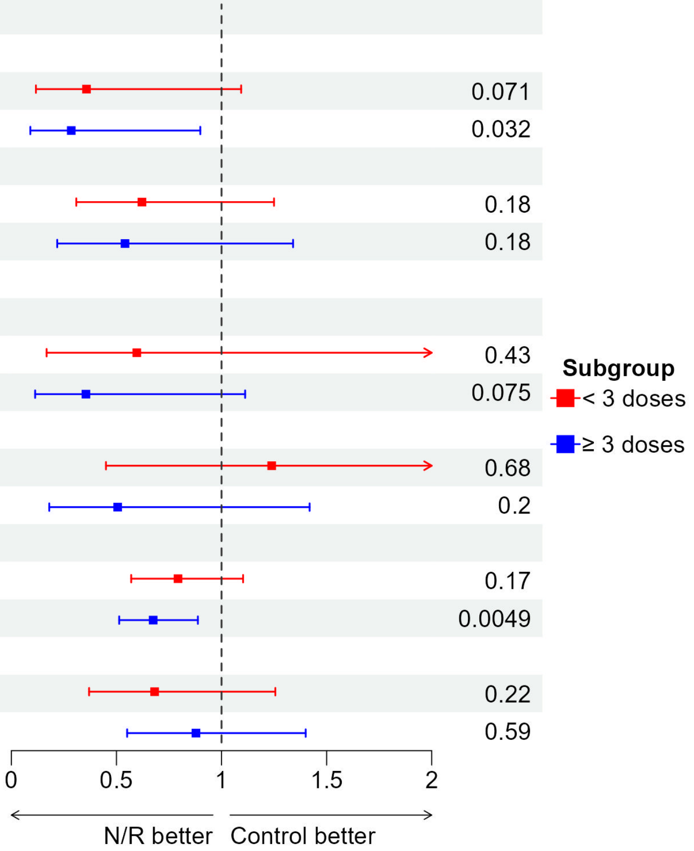

Supplement Figure 12B. Subgroup analysis: vaccination status in the comparison between the molnupiravir group and control group

Outcome	Control (Event/No. at risk [%])	Molnupiravir (Event/No. at risk [%])	HR (95% CI)	P value
Death				
Day 0-30				
< 3 doses	34/387 (8.8)	10/153 (6.5)	0.53 (0.23, 1.20)	0.13
≥ 3 doses	19/359 (5.3)	6/225 (2.7)	0.35 (0.10, 1.20)	0.094
Day 31-365				
< 3 doses	56/387 (14.5)	18/153 (11.8)	0.63 (0.35, 1.15)	0.13
≥ 3 doses	23/359 (6.4)	13/225 (5.8)	0.84 (0.34, 2.07)	0.71
ICU admission or respiratory support				
Day 0-30				
< 3 doses	19/387 (4.9)	5/153 (3.3)	1.50 (0.44, 5.14)	0.52
≥ 3 doses	17/359 (4.7)	3/225 (1.3)	0.36 (0.09, 1.38)	0.14
Day 31-365				
< 3 doses	26/387 (6.7)	8/153 (5.2)	0.89 (0.33, 2.35)	0.81
≥ 3 doses	20/359 (5.6)	11/225 (4.9)	0.90 (0.33, 2.48)	0.84
All-cause hospitalization				
< 3 doses	198/387 (51.2)	88/153 (57.5)	0.98 (0.73, 1.31)	0.89
≥ 3 doses	164/359 (45.7)	126/225 (56.0)	1.08 (0.79, 1.46)	0.64
Asthma exacerbation				
< 3 doses	64/387 (16.5)	29/153 (19.0)	1.16 (0.70, 1.94)	0.56
≥ 3 doses	60/359 (16.7)	36/225 (16.0)	1.08 (0.62, 1.86)	0.79

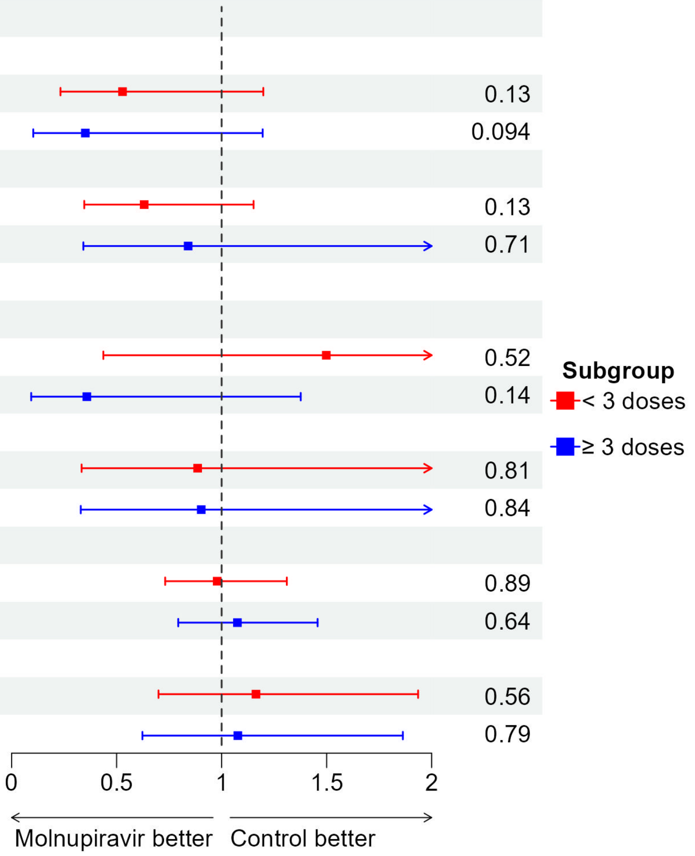

Supplement Figure 12C. Subgroup analysis: vaccination status in the comparison between the nirmatrelvir/ritonavir group and molnupiravir group

Outcome	Molnupiravir (Event/No. at risk [%])	N/R (Event/No. at risk [%])	HR (95% CI)	P value
Death				
Day 0-30				
< 3 doses	10/153 (6.5)	4/191 (2.1)	0.74 (0.20, 2.80)	0.66
≥ 3 doses	6/225 (2.7)	5/430 (1.2)	0.70 (0.17, 2.84)	0.62
Day 31-365				
< 3 doses	18/153 (11.8)	15/191 (7.9)	1.60 (0.74, 3.49)	0.23
≥ 3 doses	13/225 (5.8)	10/430 (2.3)	0.87 (0.34, 2.25)	0.77
ICU admission or respiratory support				
Day 0-30				
< 3 doses	5/153 (3.3)	5/191 (2.6)	1.32 (0.31, 5.70)	0.71
≥ 3 doses	3/225 (1.3)	6/430 (1.4)	2.69 (0.60, 12.08)	0.2
Day 31-365				
< 3 doses	8/153 (5.2)	12/191 (6.3)	1.46 (0.50, 4.24)	0.49
≥ 3 doses	11/225 (4.9)	9/430 (2.1)	0.69 (0.22, 2.16)	0.53
All-cause hospitalization				
< 3 doses	88/153 (57.5)	86/191 (45.0)	0.64 (0.45, 0.91)	0.013
≥ 3 doses	126/225 (56.0)	146/430 (34.0)	0.63 (0.47, 0.85)	0.0021
Asthma exacerbation				
< 3 doses	29/153 (19.0)	22/191 (11.5)	0.69 (0.36, 1.30)	0.25
≥ 3 doses	36/225 (16.0)	48/430 (11.2)	0.82 (0.49, 1.40)	0.47

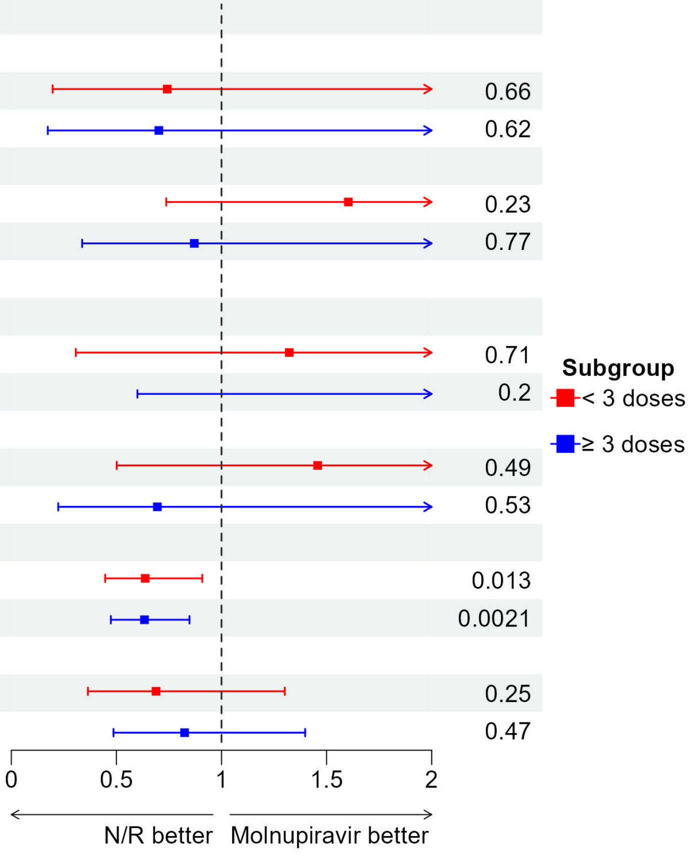

Supplement Figure 13A. Subgroup analysis: inhaled corticosteroid (ICS) dose level in the comparison between the nirmatrelvir/ritonavir group and control group

Outcome	Control (Event/No. at risk [%])	N/R (Event/No. at risk [%])	HR (95% CI)	P value
Death				
Day 0-30				
None or low	23/405 (5.7)	5/372 (1.3)	0.34 (0.11, 1.05)	0.061
Medium or high	30/341 (8.8)	4/249 (1.6)	0.19 (0.06, 0.60)	0.0046
Day 31-365				
None or low	25/405 (6.2)	9/372 (2.4)	0.33 (0.13, 0.81)	0.016
Medium or high	54/341 (15.8)	16/249 (6.4)	0.80 (0.40, 1.60)	0.52
ICU admission or respiratory support				
Day 0-30				
None or low	15/405 (3.7)	3/372 (0.8)	0.21 (0.05, 0.89)	0.034
Medium or high	21/341 (6.2)	8/249 (3.2)	0.72 (0.25, 2.05)	0.54
Day 31-365				
None or low	8/405 (2.0)	8/372 (2.2)	0.81 (0.25, 2.64)	0.73
Medium or high	38/341 (11.1)	13/249 (5.2)	0.72 (0.32, 1.62)	0.42
All-cause hospitalization				
None or low	160/405 (39.5)	111/372 (29.8)	0.60 (0.44, 0.81)	0.00083
Medium or high	202/341 (59.2)	121/249 (48.6)	0.96 (0.70, 1.33)	0.82
Asthma exacerbation				
None or low	36/405 (8.9)	31/372 (8.3)	0.91 (0.50, 1.64)	0.75
Medium or high	88/341 (25.8)	39/249 (15.7)	0.84 (0.52, 1.35)	0.46

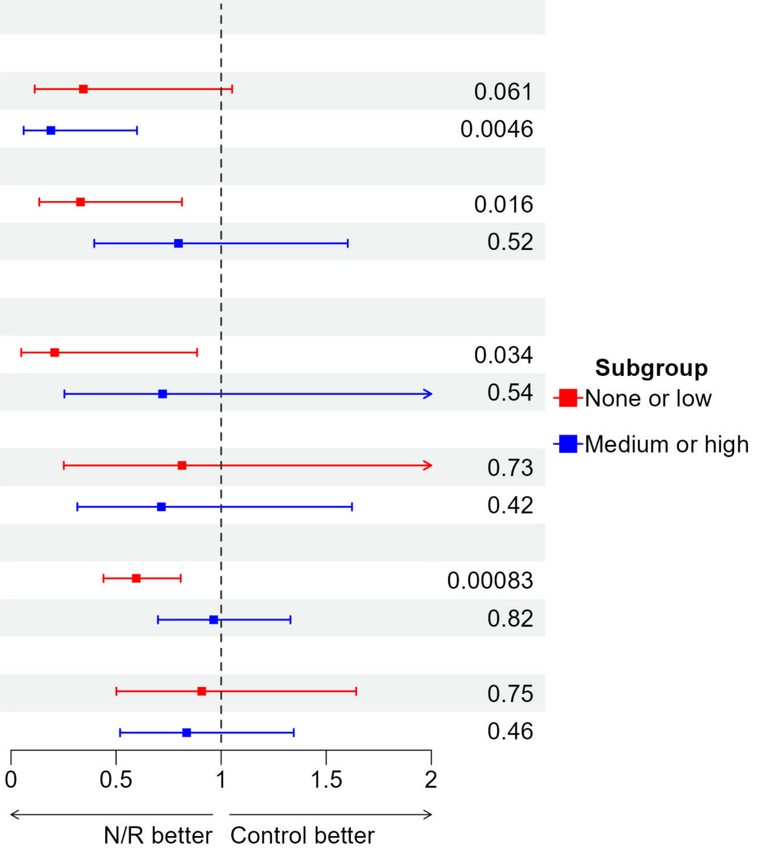

Supplement Figure 13B. Subgroup analysis: inhaled corticosteroid (ICS) dose level in the comparison between the molnupiravir group and control group

Outcome	Control (Event/No. at risk [%])	Molnupiravir (Event/No. at risk [%])	HR (95% CI)	P value
Death				
Day 0-30				
None or low	23/405 (5.7)	4/180 (2.2)	0.35 (0.11, 1.11)	0.074
Medium or high	30/341 (8.8)	12/198 (6.1)	0.52 (0.25, 1.07)	0.077
Day 31-365				
None or low	25/405 (6.2)	11/180 (6.1)	0.67 (0.29, 1.53)	0.34
Medium or high	54/341 (15.8)	20/198 (10.1)	0.90 (0.50, 1.62)	0.73
ICU admission or respiratory support				
Day 0-30				
None or low	15/405 (3.7)	2/180 (1.1)	0.38 (0.07, 2.01)	0.26
Medium or high	21/341 (6.2)	6/198 (3.0)	0.78 (0.26, 2.34)	0.66
Day 31-365				
None or low	8/405 (2.0)	5/180 (2.8)	1.58 (0.44, 5.66)	0.48
Medium or high	38/341 (11.1)	14/198 (7.1)	0.95 (0.41, 2.21)	0.9
All-cause hospitalization				
None or low	160/405 (39.5)	95/180 (52.8)	0.96 (0.69, 1.32)	0.8
Medium or high	202/341 (59.2)	119/198 (60.1)	1.10 (0.82, 1.45)	0.53
Asthma exacerbation				
None or low	36/405 (8.9)	18/180 (10.0)	0.93 (0.47, 1.86)	0.84
Medium or high	88/341 (25.8)	47/198 (23.7)	1.39 (0.89, 2.17)	0.15

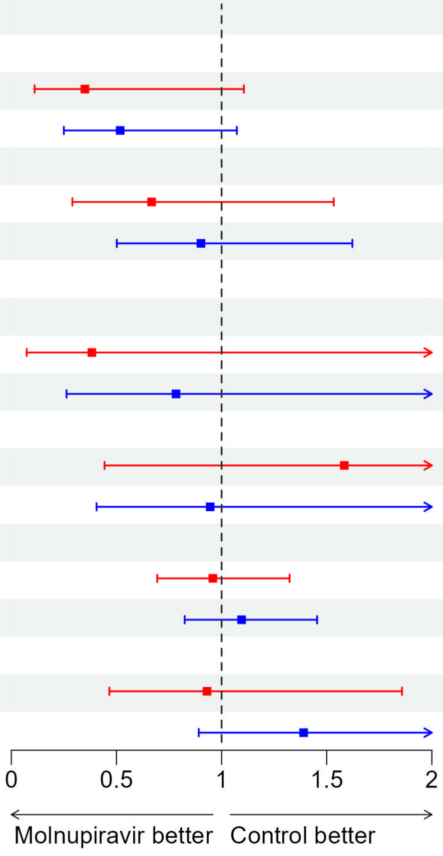

Subgroup
None or low
Medium or high

Supplement Figure 13C. Subgroup analysis: inhaled corticosteroid (ICS) dose level in the comparison between the nirmatrelvir/ritonavir group and molnupiravir group

Outcome	Molnupiravir (Event/No. at risk [%])	N/R (Event/No. at risk [%])	HR (95% CI)	P value
Death				
Day 0-30				
None or low	4/180 (2.2)	5/372 (1.3)	2.04 (0.37, 11.23)	0.41
Medium or high	12/198 (6.1)	4/249 (1.6)	0.44 (0.12, 1.59)	0.21
Day 31-365				
None or low	11/180 (6.1)	9/372 (2.4)	0.62 (0.18, 2.11)	0.44
Medium or high	20/198 (10.1)	16/249 (6.4)	1.13 (0.53, 2.43)	0.75
ICU admission or respiratory support				
Day 0-30				
None or low	2/180 (1.1)	3/372 (0.8)	1.39 (0.20, 9.84)	0.74
Medium or high	6/198 (3.0)	8/249 (3.2)	1.39 (0.41, 4.73)	0.60
Day 31-365				
None or low	5/180 (2.8)	8/372 (2.2)	1.01 (0.23, 4.41)	0.99
Medium or high	14/198 (7.1)	13/249 (5.2)	0.92 (0.39, 2.21)	0.86
All-cause hospitalization				
None or low	95/180 (52.8)	111/372 (29.8)	0.58 (0.42, 0.81)	0.0014
Medium or high	119/198 (60.1)	121/249 (48.6)	0.72 (0.53, 1.00)	0.047
Asthma exacerbation				
None or low	18/180 (10.0)	31/372 (8.3)	1.03 (0.49, 2.13)	0.94
Medium or high	47/198 (23.7)	39/249 (15.7)	0.58 (0.35, 0.95)	0.030

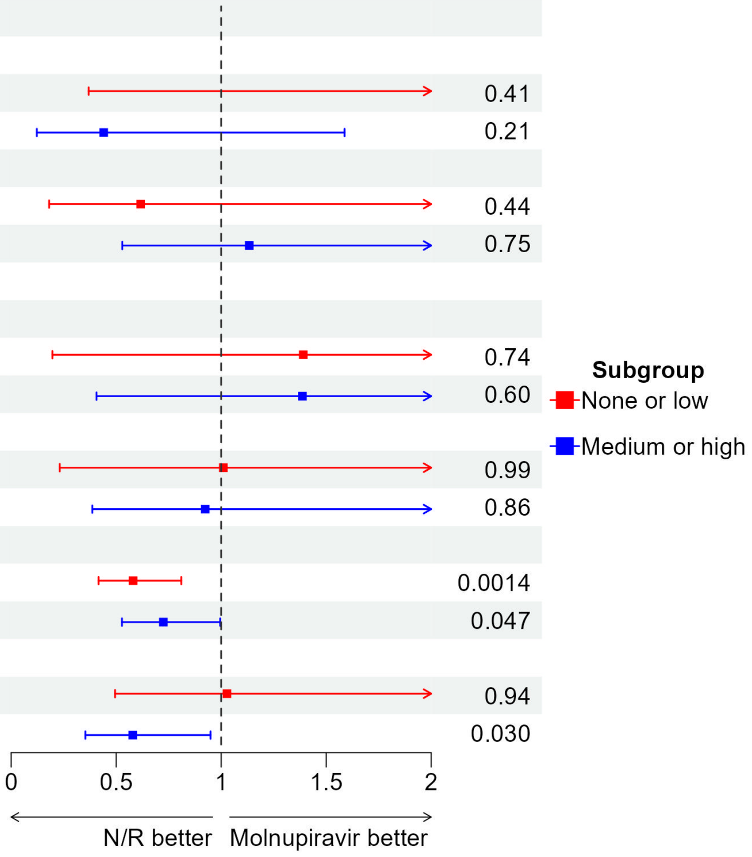

Supplement Figure 14A. Subgroup analysis: hospital admission on index date in the comparison between the nirmatrelvir/ritonavir group and control group

Outcome	Control (Event/No. at risk [%])	N/R (Event/No. at risk [%])	HR (95% CI)	P value
Death				
Day 0-30				
Non-hospital admission	18/410 (4.4)	5/380 (1.3)	0.68 (0.22, 2.13)	0.51
Hospital admission	35/336 (10.4)	4/241 (1.7)	0.18 (0.06, 0.54)	0.0021
Day 31-365				
Non-hospital admission	24/410 (5.9)	9/380 (2.4)	0.38 (0.15, 0.95)	0.038
Hospital admission	55/336 (16.4)	16/241 (6.6)	0.53 (0.25, 1.10)	0.089
ICU admission or respiratory support				
Day 0-30				
Non-hospital admission	8/410 (2.0)	4/380 (1.1)	0.44 (0.08, 2.43)	0.35
Hospital admission	28/336 (8.3)	7/241 (2.9)	0.64 (0.24, 1.73)	0.38
Day 31-365				
Non-hospital admission	13/410 (3.2)	9/380 (2.4)	0.67 (0.23, 1.93)	0.46
Hospital admission	33/336 (9.8)	12/241 (5.0)	0.76 (0.30, 1.92)	0.56
All-cause hospitalization				
Non-hospital admission	164/410 (40.0)	108/380 (28.4)	0.64 (0.48, 0.87)	0.0039
Hospital admission	198/336 (58.9)	124/241 (51.5)	0.78 (0.57, 1.06)	0.11
Asthma exacerbation				
Non-hospital admission	59/410 (14.4)	36/380 (9.5)	0.75 (0.45, 1.24)	0.26
Hospital admission	65/336 (19.3)	34/241 (14.1)	0.96 (0.55, 1.68)	0.89

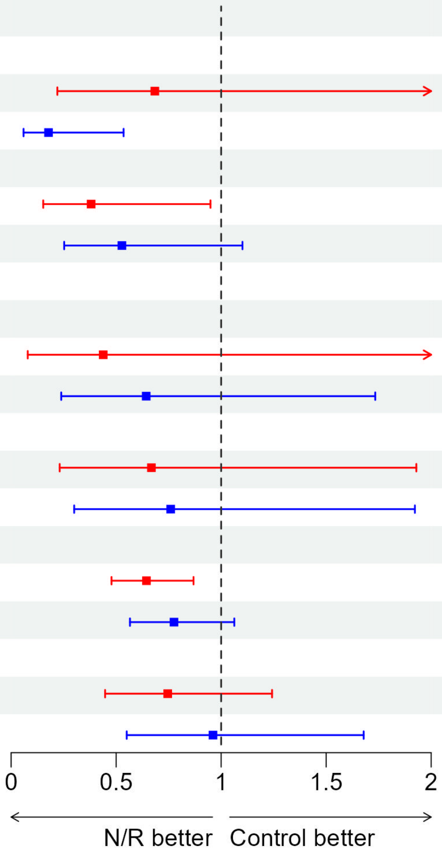

Supplement Figure 14B. Subgroup analysis: hospital admission on index date in the comparison between the molnupiravir group and control group

Outcome	Control (Event/No. at risk [%])	Molnupiravir (Event/No. at risk [%])	HR (95% CI)	P value
Death				
Day 0-30				
Non-hospital admission	18/410 (4.4)	5/222 (2.3)	0.33 (0.11, 0.98)	0.045
Hospital admission	35/336 (10.4)	11/156 (7.1)	0.46 (0.19, 1.09)	0.076
Day 31-365				
Non-hospital admission	24/410 (5.9)	15/222 (6.8)	0.61 (0.29, 1.25)	0.18
Hospital admission	55/336 (16.4)	16/156 (10.3)	0.77 (0.40, 1.48)	0.44
ICU admission or respiratory support				
Day 0-30				
Non-hospital admission	8/410 (2.0)	2/222 (0.9)	0.33 (0.06, 1.76)	0.2
Hospital admission	28/336 (8.3)	6/156 (3.8)	0.97 (0.35, 2.70)	0.96
Day 31-365				
Non-hospital admission	13/410 (3.2)	9/222 (4.1)	0.82 (0.31, 2.14)	0.68
Hospital admission	33/336 (9.8)	10/156 (6.4)	1.07 (0.37, 3.05)	0.9
All-cause hospitalization				
Non-hospital admission	164/410 (40.0)	118/222 (53.2)	1.04 (0.77, 1.39)	0.81
Hospital admission	198/336 (58.9)	96/156 (61.5)	0.95 (0.70, 1.27)	0.72
Asthma exacerbation				
Non-hospital admission	59/410 (14.4)	32/222 (14.4)	0.76 (0.45, 1.30)	0.32
Hospital admission	65/336 (19.3)	33/156 (21.2)	1.54 (0.90, 2.63)	0.12

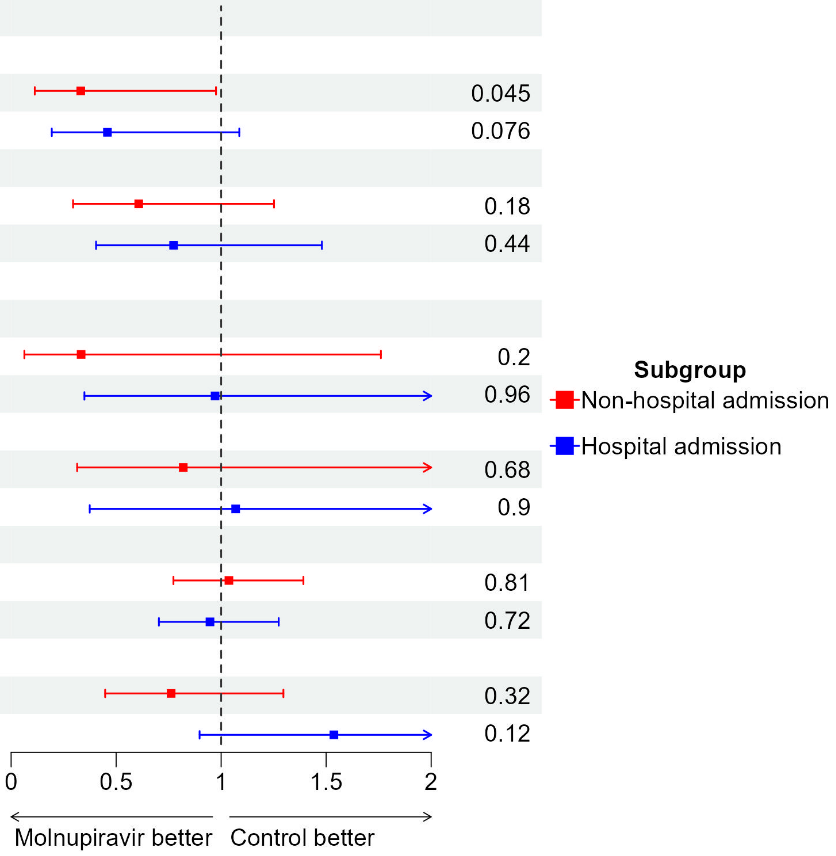

Supplement Figure 14C. Subgroup analysis: hospital admission on index date in the comparison between the nirmatrelvir/ritonavir group and molnupiravir group

Outcome	Molnupiravir (Event/No. at risk [%])	N/R (Event/No. at risk [%])	HR (95% CI)	P value
Death				
Day 0-30				
Non-hospital admission	5/222 (2.3)	5/380 (1.3)	3.16 (0.79, 12.58)	0.1
Hospital admission	11/156 (7.1)	4/241 (1.7)	0.26 (0.07, 1.05)	0.058
Day 31-365				
Non-hospital admission	15/222 (6.8)	9/380 (2.4)	1.15 (0.45, 2.93)	0.78
Hospital admission	16/156 (10.3)	16/241 (6.6)	0.78 (0.33, 1.84)	0.57
ICU admission or respiratory support				
Day 0-30				
Non-hospital admission	2/222 (0.9)	4/380 (1.1)	8.88 (1.30, 60.79)	0.026
Hospital admission	6/156 (3.8)	7/241 (2.9)	0.95 (0.27, 3.28)	0.93
Day 31-365				
Non-hospital admission	9/222 (4.1)	9/380 (2.4)	0.93 (0.27, 3.22)	0.91
Hospital admission	10/156 (6.4)	12/241 (5.0)	0.87 (0.31, 2.47)	0.8
All-cause hospitalization				
Non-hospital admission	118/222 (53.2)	108/380 (28.4)	0.59 (0.42, 0.82)	0.0016
Hospital admission	96/156 (61.5)	124/241 (51.5)	0.72 (0.52, 0.99)	0.042
Asthma exacerbation				
Non-hospital admission	32/222 (14.4)	36/380 (9.5)	0.77 (0.40, 1.47)	0.43
Hospital admission	33/156 (21.2)	34/241 (14.1)	0.49 (0.29, 0.84)	0.0092

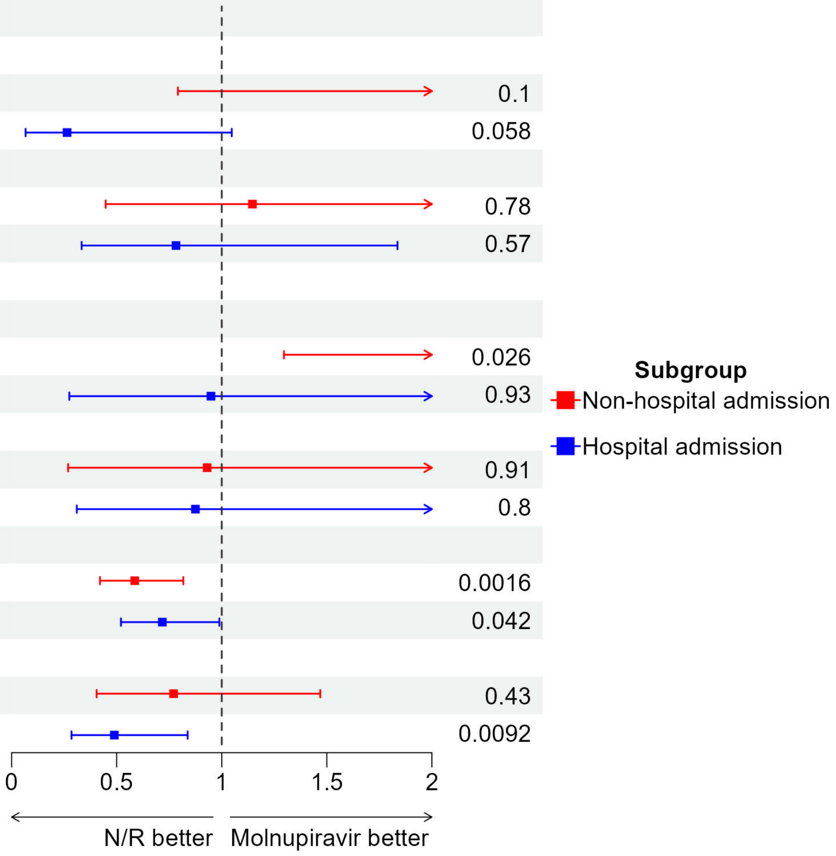

Supplement Figure 15. Sensitivity analysis 1: Comparison between the molnupiravir group and control group with the start of the study period as the date when molnupiravir became available (Feb. 26, 2022) and without excluding individuals with contraindications to nirmatrelvir/ritonavir

Outcome	Control (Event/No. at risk [%])	Molnupiravir (Event/No. at risk [%])	HR (95% CI)	P value
Death				
Day 0-30	144/1146 (12.6)	24/460 (5.2)	0.29 (0.18, 0.48)	<0.0001
Day 31-365	128/1146 (11.2)	46/460 (10.0)	0.76 (0.51, 1.13)	0.17
ICU admission or respiratory support				
Day 0-30	59/1146 (5.1)	10/460 (2.2)	0.48 (0.22, 1.04)	0.065
Day 31-365	73/1146 (6.4)	23/460 (5.0)	0.90 (0.50, 1.62)	0.72
All-cause hospitalization	545/1146 (47.6)	265/460 (57.6)	0.98 (0.82, 1.17)	0.83
Cause-specific hospitalization				
Pulmonary conditions				
Chronic pulmonary disease excluding asthma	21/895 (2.3)	11/377 (2.9)	0.50 (0.22, 1.11)	0.088
Acute respiratory distress syndrome	34/1040 (3.3)	11/426 (2.6)	0.66 (0.30, 1.45)	0.31
Cardiovascular conditions				
Myocardial infarction	17/1114 (1.5)	3/439 (0.7)	0.35 (0.09, 1.34)	0.13
Stroke	15/1087 (1.4)	9/424 (2.1)	1.40 (0.53, 3.74)	0.50
Asthma exacerbation	171/1146 (14.9)	79/460 (17.2)	1.23 (0.88, 1.72)	0.22

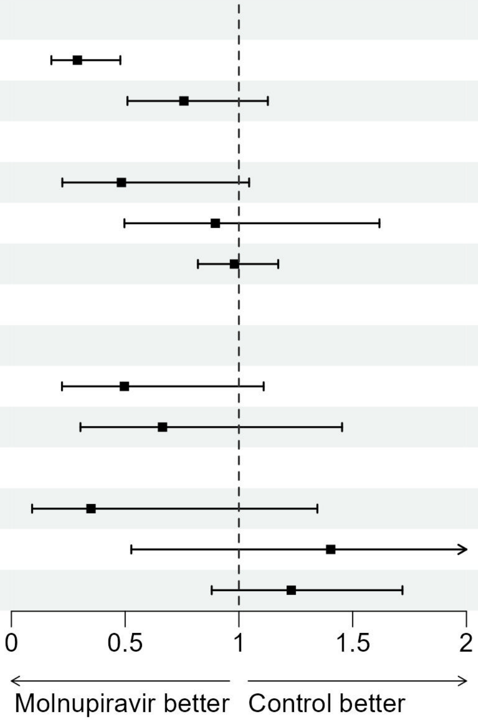

Supplement Figure 16. Sensitivity analysis 2: Comparison between the nirmatrelvir/ritonavir group and control group excluding patients who were prescribed salmeterol within 10 days of the index date

Outcome	Control (Event/No. at risk [%])	N/R (Event/No. at risk [%])	HR (95% CI)	P value
Death				
Day 0-30	50/707 (7.1)	9/608 (1.5)	0.27 (0.12, 0.60)	0.0014
Day 31-365	72/707 (10.2)	25/608 (4.1)	0.50 (0.29, 0.88)	0.016
ICU admission or respiratory support				
Day 0-30	35/707 (5.0)	11/608 (1.8)	0.44 (0.18, 1.04)	0.063
Day 31-365	39/707 (5.5)	20/608 (3.3)	0.93 (0.46, 1.92)	0.85
All-cause hospitalization	340/707 (48.1)	225/608 (37.0)	0.69 (0.56, 0.86)	0.00076
Cause-specific hospitalization				
Pulmonary conditions				
Chronic pulmonary disease excluding asthma	16/573 (2.8)	7/529 (1.3)	0.35 (0.13, 0.95)	0.040
Acute respiratory distress syndrome	20/650 (3.1)	8/581 (1.4)	0.48 (0.18, 1.28)	0.14
Cardiovascular conditions				
Myocardial infarction	10/687 (1.5)	1/602 (0.2)	0.10 (0.01, 0.93)	0.043
Stroke	5/675 (0.7)	6/590 (1.0)	3.02 (0.73, 12.52)	0.13
Asthma exacerbation	115/707 (16.3)	65/608 (10.7)	0.79 (0.54, 1.14)	0.21

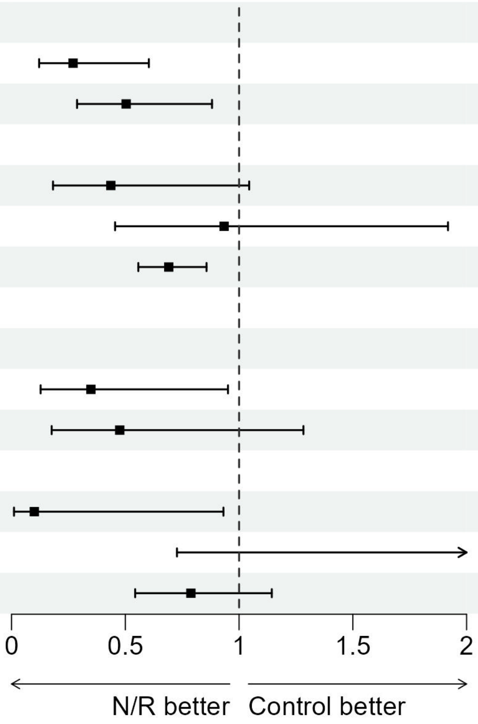

Supplement Figure 17. Sensitivity analysis 3: Comparison between the nirmatrelvir/ritonavir group and molnupiravir group excluding patients who were prescribed salmeterol within 10 days of the index date

Outcome	Molnupiravir (Event/No. at risk [%])	N/R (Event/No. at risk [%])	HR (95% CI)	P value
Death				
Day 0-30	13/333 (3.9)	9/608 (1.5)	0.66 (0.21, 2.04)	0.47
Day 31-365	26/333 (7.8)	25/608 (4.1)	0.87 (0.45, 1.69)	0.68
ICU admission or respiratory support				
Day 0-30	7/333 (2.1)	11/608 (1.8)	1.52 (0.47, 4.88)	0.49
Day 31-365	14/333 (4.2)	20/608 (3.3)	0.92 (0.41, 2.04)	0.84
All-cause hospitalization	185/333 (55.6)	225/608 (37.0)	0.66 (0.53, 0.84)	0.00053
Cause-specific hospitalization				
Pulmonary conditions				
Chronic pulmonary disease excluding asthma	9/278 (3.2)	7/529 (1.3)	0.62 (0.20, 1.93)	0.41
Acute respiratory distress syndrome	9/315 (2.9)	8/581 (1.4)	0.55 (0.20, 1.51)	0.25
Cardiovascular conditions				
Myocardial infarction	2/320 (0.6)	1/602 (0.2)	0.69 (0.06, 8.12)	0.77
Stroke	3/308 (1.0)	6/590 (1.0)	2.61 (0.43, 15.91)	0.3
Asthma exacerbation	48/333 (14.4)	65/608 (10.7)	0.81 (0.52, 1.25)	0.33

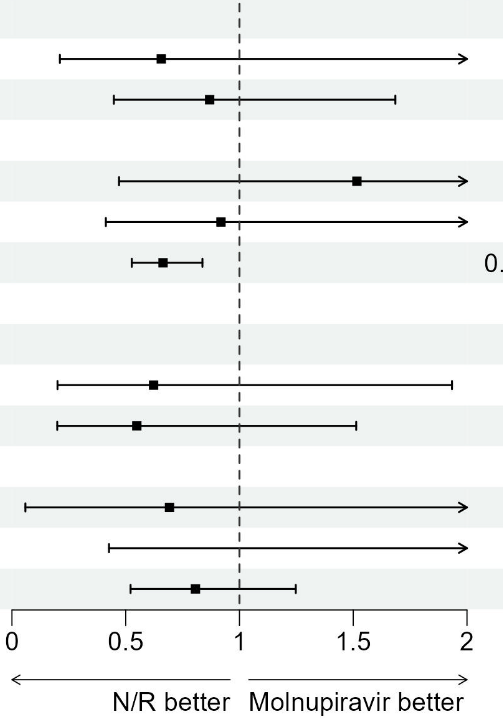

References

1. Global Initiative for Asthma. Global Strategy for Asthma Management and Prevention, 2024. Updated May 2024. Available from: www.ginasthma.org
2. Lam ICH, Wong CKH, Zhang R, Chui CSL, Lai FTT, Li X, Chan EWY, Luo H, Zhang Q, Man KKC, Cheung BMY, Tang SCW, Lau CS, Wan EYF, Wong ICK. Long-term post-acute sequelae of COVID-19 infection: a retrospective, multi-database cohort study in Hong Kong and the UK. EClinicalMedicine. 2023 Jun;60:102000.
3. Quan H, Sundararajan V, Halfon P, Fong A, Burnand B, Luthi JC, Saunders LD, Beck CA, Feasby TE, Ghali WA. Coding algorithms for defining comorbidities in ICD-9-CM and ICD-10 administrative data. Med Care. 2005 Nov;43(11):1130-9. doi: 10.1097/01.mlr.0000182534.19832.83. PMID: 16224307.
